# Validity of Diagnostic Codes for Acute Stroke in Administrative Databases: A Systematic Review

**DOI:** 10.1371/journal.pone.0135834

**Published:** 2015-08-20

**Authors:** Natalie McCormick, Vidula Bhole, Diane Lacaille, J. Antonio Avina-Zubieta

**Affiliations:** 1 Faculty of Pharmaceutical Sciences, University of British Columbia, Vancouver, British Columbia, Canada; 2 Arthritis Research Canada, Richmond, British Columbia, Canada; 3 Division of Rheumatology, Department of Medicine. University of British Columbia, Vancouver, British Columbia, Canada; 4 Cardiovascular Committee of the CANRAD Network, Richmond, British Columbia, Canada; University of Glasgow, UNITED KINGDOM

## Abstract

**Objective:**

To conduct a systematic review of studies reporting on the validity of International Classification of Diseases (ICD) codes for identifying stroke in administrative data.

**Methods:**

MEDLINE and EMBASE were searched (inception to February 2015) for studies: (a) Using administrative data to identify stroke; or (b) Evaluating the validity of stroke codes in administrative data; and (c) Reporting validation statistics (sensitivity, specificity, positive predictive value (PPV), negative predictive value (NPV), or Kappa scores) for stroke, or data sufficient for their calculation. Additional articles were located by hand search (up to February 2015) of original papers. Studies solely evaluating codes for transient ischaemic attack were excluded. Data were extracted by two independent reviewers; article quality was assessed using the Quality Assessment of Diagnostic Accuracy Studies tool.

**Results:**

Seventy-seven studies published from 1976–2015 were included. The sensitivity of ICD-9 430-438/ICD-10 I60-I69 for any cerebrovascular disease was ≥ 82% in most [≥ 50%] studies, and specificity and NPV were both ≥ 95%. The PPV of these codes for any cerebrovascular disease was ≥ 81% in most studies, while the PPV specifically for acute stroke was ≤ 68%. In at least 50% of studies, PPVs were ≥ 93% for subarachnoid haemorrhage (ICD-9 430/ICD-10 I60), 89% for intracerebral haemorrhage (ICD-9 431/ICD-10 I61), and 82% for ischaemic stroke (ICD-9 434/ICD-10 I63 or ICD-9 434&436). For in-hospital deaths, sensitivity was 55%. For cerebrovascular disease or acute stroke as a cause-of-death on death certificates, sensitivity was ≤ 71% in most studies while PPV was ≥ 87%.

**Conclusions:**

While most cases of prevalent cerebrovascular disease can be detected using 430-438/I60-I69 collectively, acute stroke must be defined using more specific codes. Most in-hospital deaths and death certificates with stroke as a cause-of-death correspond to true stroke deaths. Linking vital statistics and hospitalization data may improve the ascertainment of fatal stroke.

## Introduction

Stroke imparts a substantial burden on patients, healthcare systems, and society, with stroke accounting for more than 6.6 million deaths in 2012 (11.9% of all deaths globally) [1]. Those who survive an acute stroke are often permanently disabled, with reduced work and social activities [2], and quality of life [3]. The economic consequences are also substantial; the annual costs of stroke were recently estimated at $33.6 billion in the United States [4] and £8.9 billion in the United Kingdom [5], with direct medical costs accounting for half of these expenditures. Although the incidence of stroke has been decreasing in high-income countries, this decrease is being offset by increasing rates in low- and middle-income countries [6], such that the worldwide burden of stroke is continuing to grow.

Administrative databases are increasingly being used for stroke research. These data sources, which link longitudinal health resource utilization data for hospitalizations, outpatient care, and, in some jurisdictions, dispensed medications, to individual-level demographic and vital statistics data, allow for more efficient analyses, and more generalizable findings. Unfortunately, as administrative databases are usually established for billing, and not research, purposes, the diagnoses contained within tend to be coded by non-medical staff and may not reflect the final diagnosis of the treating physician. But if these databases are to be used for stroke research, the diagnostic codes used to identify stroke must be valid. This means they must be able to distinguish those who have actually experienced a stroke (according to an accepted ‘gold standard’ reference diagnosis) from those who have not. These diagnostic codes must also allow researchers to distinguish the major subtypes of acute stroke, which differ from one another with regards to their incidence rates, risk factors, and outcomes. For example, haemorrhagic stroke occurs far less frequently than ischaemic stroke [4], but is associated with higher re-hospitalization rates [7,8] and earlier mortality [7,9–11], and greater short-term [8,10,12–21] and long-term [22] treatment costs.

While several validation studies of stroke codes have been conducted [23–26], these have varied widely with regards to their study populations, clinical and geographic settings, and the reference standards used. For example, while some assessed the validity of codes for just one subtype [23–25], others assessed broader groups of codes pertaining to cerebrovascular disease as a whole (including acute stroke, transient ischaemic attack, and stroke sequelae). To synthesize the current evidence, we, as part of a Canadian Rheumatology Network for establishing best practices in the use of administrative data for health research and surveillance (CANRAD)[27–31], conducted a systematic review of studies reporting on the validity of diagnostic codes for identifying cardiovascular diseases. Data from these studies were used to compare the validity of these codes, and evaluate whether administrative health data can accurately identify cardiovascular diseases for the purpose of capturing these events as covariates, outcomes, or complications in future research. We recently reported our findings on the validity of codes for myocardial infarction [32] and heart failure [33]. In the current paper, we analyze studies reporting on the validity of stroke codes in administrative databases.

## Methods

### Literature Search

An experienced librarian (M-DW) undertook searches of the MEDLINE and EMBASE databases, from inception (1946 and 1974, respectively) for all available peer-reviewed literature. Two search strategies were used: (1) All studies where administrative data was used to identify cardiovascular diseases; (2) All studies reporting on the validity of administrative data for identifying cardiovascular diseases. Our MEDLINE and EMBASE search strategies are available as (**S1, S2, S3, and S4 Texts**). To identify additional studies, the authors hand-searched the reference lists of the key articles located. As well, the Cited-By tools in PubMed and Google Scholar were used to find relevant articles that had cited the articles located through the database search. The databases were originally searched from inception to November 2010, with the handsearch conducted up to February 2011. These searches were updated in February 2015.

Two reviewers independently screened the titles and abstracts of the located records for relevance to the study objectives. In the next step, full text publications were evaluated against the inclusion criteria. Any discrepancies were discussed until consensus was reached. When the conflict persisted a third reviewer (JAA-Z) was consulted. No protocol for this systematic review has been published, though more information is available in the following publication [27]. Our review was conducted in accordance with the Preferred Reporting Items for Systematic Reviews and Meta-Analyses (PRISMA) [34] statements, and our completed PRISMA checklist is provided as (**S1 Checklist**).

### Inclusion Criteria

We considered full-length, English-language, peer-reviewed articles that used administrative data and either reported validation statistics for the International Classification of Diseases (ICD) codes of interest, or provided sufficient data for their calculation. We first included studies that evaluated at least one code pertaining to a subtype of acute stroke, being ICD-8/9 430 or ICD-10 I60 for subarachnoid haemorrhage (SAH), and ICD-8/9 431 or ICD-10 I61 for intracerebral haemorrhage (ICH). For ischaemic stroke, the main codes are ICD-8 433/434 and ICD-9 434 (occlusion of the cerebral arteries), and ICD-10 I63 (cerebral infarction).

Stroke is a heterogeneous disease that is not defined consistently by clinicians or researchers [35]. It has traditionally been distinguished from transient ischaemic attack (TIA) by way of duration (more or less than 24 hours) and the presence/absence of permanent brain infarction. Although advances in neuroimaging have resulted in many events that would previously have been labelled as TIA now being considered as minor strokes, this is an area of ongoing controversy [35]. As such, we took a conservative approach by not considering episodes of TIA as acute stroke, and so excluded studies that solely evaluated codes for TIA (ICD-9 435 or ICD-10 G45).

Although our focus was on the validity of codes for acute stroke-specifically (defined as SAH, ICH, or ischaemic stroke), we also included studies that evaluated a range of codes (ICD-8/9 430–438 or ICD-10 I60-I69) pertaining to a broader group of cerebrovascular diseases. Included in these ranges were the codes for acute stroke listed above, along with codes for acute but ill-defined stroke (ICD-9 436 and ICD-10 I64), other types of ill-defined stroke (ICD-9 437) and other cerebrovascular diseases (ICD-10 I67/68), other types of intracranial haemorrhage than ICH (ICD-9 432 and ICD-10 I62), TIA (ICD-9 435), and late effects of stroke or stroke sequelae (ICD-9 438 and ICD-10 I69). It was important to include these studies because, while reviewing the literature, we observed that this broad range of codes for cerebrovascular disease is frequently used to identify cases of acute stroke.

### Data Extraction

Two independent reviewers (NM and VB) examined the full text of each selected record and abstracted data using a standardized collection form (a copy is provided in **S5 Text**). Information was gathered on the study population, administrative data source, stroke codes and algorithm, validation process, and gold standard. Validation statistics comparing the codes to definite, probable, or possible cases of acute stroke, or the specific diagnoses of SAH, ICH, or ischaemic stroke in particular, were abstracted. These statistics included sensitivity, specificity, positive predictive value (PPV), negative predictive value (NPV), and kappa. Wherever possible, we abstracted statistics for definite and probable cases of acute stroke. However, the number of categories available depended on the choice of gold standard. For example, under the World Health Organisation criteria, potential cases are categorized as either definite stroke or not stroke [36], while the National Survey of Stroke criteria [37] use the categories of definite, highly probable, and probable stroke. Statistics for each sex, for fatal versus non-fatal cases, and for each hospital discharge position (i.e. primary/principal and secondary diagnosis) were abstracted where reported. Data were independently abstracted by each reviewer who subsequently compared their forms to correct errors and resolve discrepancies, if any.

### Quality Scores

The design and methods employed in each study, including the rigour of the reference standard, and generalizability of the study population, could influence the resultant validity statistics. Hence, all studies were evaluated for quality, with the validation statistics stratified by level of study quality. An adaptation of the QUADAS tool (Quality Assessment of Diagnostic Accuracy Studies) [38] was used to evaluate study quality. Our group previously used the QUADAS in assessing the validity of codes for diabetes mellitus [30], myocardial infarction [32], heart failure [33] and osteoporosis and fractures [31].

### Statistical Analysis

All validation statistics were abstracted as reported. Where sufficient data were available we calculated 95% confidence intervals (95% CI) and validation statistics not directly reported in the original publication. Kappa values (a measure of agreement beyond that expected by chance) greater than 0.60 indicated substantial/perfect agreement, 0.21–0.60 were considered as fair/moderate agreement and those 0.20 or lower as light/poor agreement [39].

## Results

### Literature Search

**Fig 1:**
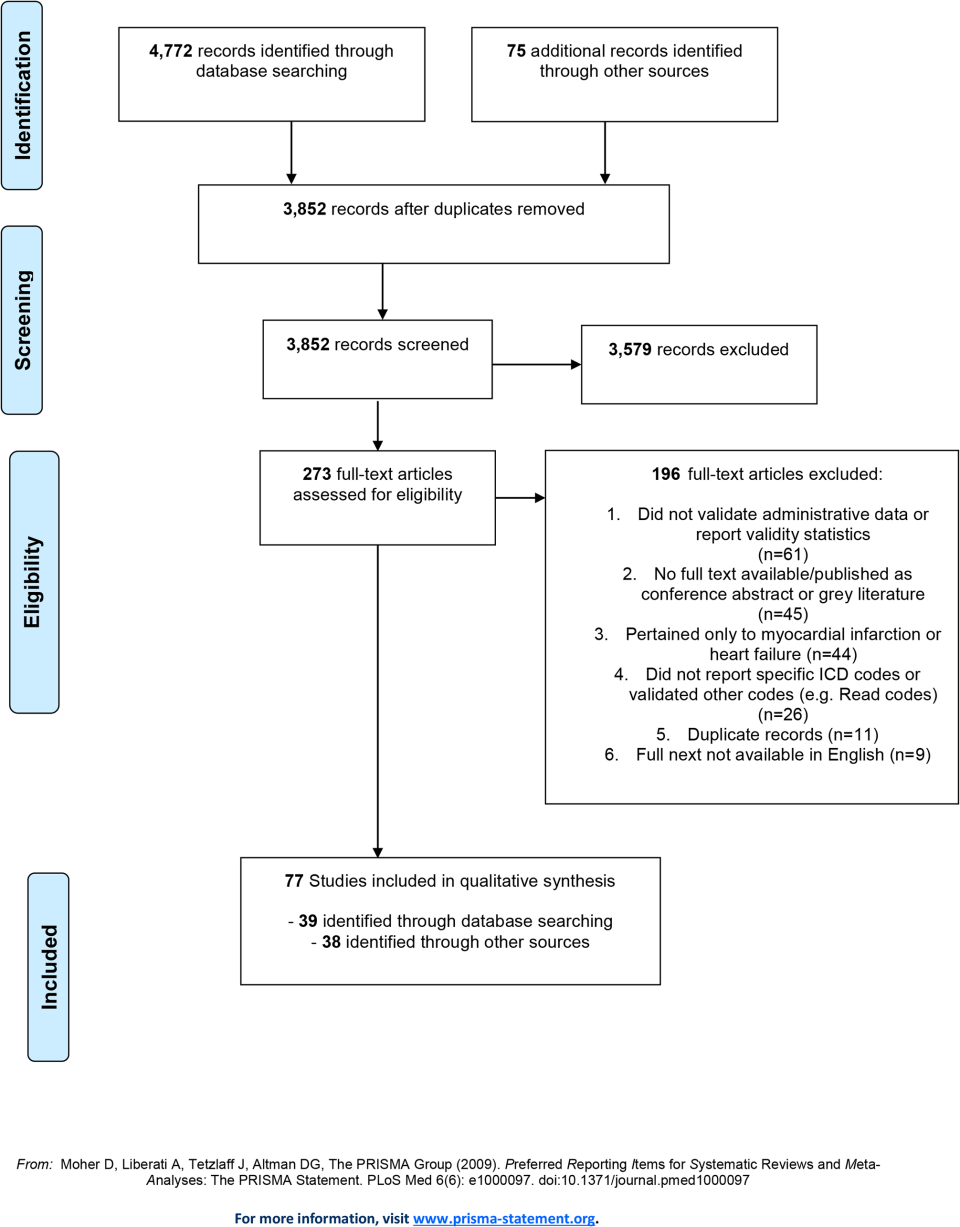
Preferred Reporting Items for Systematic Reviews and Meta-Analyses (PRISMA)-style Flowchart of Study Selection and Review. ICD = International Classification of Diseases.

We identified 1,587 citations through our original searches (inception to November 2010) of the MEDLINE and EMBASE databases, and an additional 2,160 citations in our updated searches of these databases (January 2010 to February 2015). All citations were screened for relevance to our study objectives, with 198 full-text articles assessed for eligibility (**Fig 1**), and 39 of these selected for inclusion. We also assessed 75 full-text articles for eligibility that were identified from hand searches, and selected 38 additional articles therein. Thus, a total of 273 articles were assessed for eligibility, from which 196 were excluded, mainly because they reported on the validity of other cardiovascular diseases (n = 44), or did not actually validate stroke diagnoses in administrative data (n = 61). Nine articles were excluded because they were not published in English; their languages of publication were Danish, German, Italian, Japanese, Portuguese, Spanish (two articles), French, and Chinese. Ultimately 77 articles were included for the systematic review of acute stroke.

### Study Characteristics

Of the 77 articles evaluating stroke diagnoses that were included in the final review, 31 (40%) were from the United States (USA), 26 (34%) were from Europe, 13 (17%) were from Canada, four were from Asia (5%), two (3%) were from Australia, and one (1%) was from Sri Lanka. Characteristics of these studies are presented in **Table 1**. Validation was the primary objective in all but ten [40–49] of these studies. Two articles [40,50] reported on the validity of stroke diagnoses exclusively in a paediatric population. Most studies evaluated the diagnostic codes in hospitalization databases though 16 studies [47,51–65] evaluated stroke as a cause-of-death on death certificates and one study [66] reported on outpatient data exclusively.

**Table 1:** Characteristics of Included Studies.

First Author, Year of Publication	Year(s) of Data Collection	Primary Validation Study?	Country	Records Evaluated (N)	Source Population	Type of Administrative Data	Gold Standard
**Aboa-Eboule** [67], 2013	2004–2008	yes	France	903	residents of one community hospitalized for stroke at one teaching hospital	ICD-10 inpatient records	disease registry, using WHO criteria
**Agrawal** [40], 2009	1993–2003	no	USA (California)	1,307	children aged 0–19 years enrolled in the Kaiser Permanente Medical Care Program and participating in the Kaiser Paediatric Stroke Study	ICD-9 inpatient and outpatient records	CRMD
**Appelros** [65], 2011	1999–2000	yes	Sweden	377	residents of one community	ICD-10 inpatient and vital statistics records	disease registry, using WHO criteria
**Arnason** [68], 2006	1999–2000	yes	Canada (Ontario)	616	all patient discharged from one tertiary hospital with a bleeding-related or thromboembolic diagnosis	ICD-9 inpatient records	CRDC
**Benesch** [69], 1997	1992	yes	USA (Louisiana, Massachusetts, California, Iowa, Pennsylvania)	649	patients hospitalized at one of five academic medical centres and eligible for a telephone survey of persons at increased risk for major stroke	ICD-9 inpatient records	CRDC—WHO criteria
**Birman-Deych** [70], 2005	1998–1999	yes	USA (national)	23,657	Medicare beneficiaries (aged 20–105 years) on the National Registry of Atrial Fibrillation hospitalized for atrial fibrillation	ICD-9 inpatient records	chart review
**Borzecki** [66], 2004	1998–1999	yes	USA (national)	1,176	individuals regularly receiving care from one of 10 Veterans Affairs sites across the USA, random selection of 100 users from each site with hypertension and 20 without	ICD-9 outpatient records	chart review
**Broderick** [41], 1998	1993–1995	no	USA (Ohio, Kentucky)	733	all residents of one of five counties	ICD-9 inpatient and vital statistics records	CRDC–Rochester, Minnesota and National Institute of Neurological Disorders and Stroke
**Brown** [54], 2006	2000–2001	yes	USA (Texas)	186	participants aged 44 years and older enrolled in the Brain Attack Surveillance in Corpus Christi (BASIC) Project	ICD-10 vital statistics records	CRMD
**Chen** [71], 2009	2003	yes	Canada (Alberta)	4,008	general hospitalized population	ICD-10 inpatient records	chart review
**Cheng** [72], 2011	1999	yes	Taiwan	372	hospitalized patients aged 55 years and older	ICD-9 inpatient records	CRMD
**Davenport** [73], 1996	n/a	yes	Scotland	97,515	hospitalized patients at one university teaching hospital	ICD-9 inpatient records	disease registry: Lothian Stroke Register
**de Faire** [63], 1976	1961–73	yes	Sweden	1,156	10,000 pairs of twins enrolled in the Swedish Twin Registry and born during 1901–1925	ICD (1965 edition) vital statistics records	CRMD
**Derby** [42], 2000	1980–1991	no	USA (Rhode Island, Massachusetts)	3,811	residents of two communities aged 35–74 years	ICD-9 inpatient records	CRMD
**Ellekjaer** [74], 1999	1994–1996	yes	Norway	759	hospitalized patients aged 15 years and older	ICD-9 inpatient records	disease registry, using WHO criteria
**Gaist** [43], 2000	1977–1995	no	Denmark	191	patients hospitalized at one university hospital or two other hospitals within one county	ICD-8 and ICD-10 inpatient records	CRMD
**Ghia** [75], 2010	2003–2007	yes	Australia	570	hospitalized patients admitted through the emergency department and diagnosed upon admission with TIA	ICD-10 inpatient records	chart review
**Goldstein** [25], 1998	1995–1997	yes	USA (North Carolina)	175	hospitalized patients at one Veterans Affairs Medical Center	ICD-9 inpatient records	CRDC—TOAST criteria
**Golomb** [50], 2006	1999–2004	yes	USA (Indiana)	663	all inpatients and outpatients seen at one Children's Hospital	ICD-9 inpatient and outpatient records	CRMD
**Haesebaert** [76], 2013	2006–2007	yes	France	329	patients ≥ 18 years of age admitted to one of four university hospitals	ICD-10 inpatient records	disease registry: AVC69 cohort
**Hasan** [77], 1995	1993	yes	Wales	166	patients admitted to the Department of the Care of the Elderly at one of four hospitals within one health unit	ICD-9 inpatient records	CRMD
**Heckbert** [78], 2004	1994–2000	yes	USA (national)	34,016	women participating in the Women's Health Initiative clinical and observational studies	ICD-9 inpatient records	CRDC—Women's Health Initiative criteria
**Henderson** [79], 2006	1998–99, 2000–01	yes	Australia	14,635	all hospitalized patients (excluding same-day chemotherapy and dialysis)	ICD-10 inpatient records	chart review: charts were re-coded by professional coders
**Hennessy** [80], 2010	2002–2007	yes	Canada	1,292	patients hospitalized at one of four hospitals	ICD-10 inpatient records	chart review: charts were re-coded by nurses with coding experience
**Holick** [44], 2009	2003–2007	no	USA	132	new users of atomoxetine or stimulant ADHD medications, and general population controls, identified from a health insurance database for a study assessing the association between atomoxetine and stroke in adults	ICD-9 inpatient records	CRMD
**Hsieh** [81], 2013	2006–2008	yes	Taiwan	1,736	patients hospitalized at one tertiary referral centre	ICD-9 inpatient records	disease registry (Taiwan Stroke Registry) and CRMD
**Humphries** [82], 2000	1994–1995	yes	Canada (British Columbia)	817	patients hospitalized for percutaneous coronary intervention	ICD-9 inpatient records	chart review
**Iso** [57], 1990	1970 & 1980	yes	USA (Minnesota)	214	residents of the study area aged 30–74 years who died in hospital, identified as part of the Minnesota Heart Survey	ICD-8 and ICD-9 vital statistics records	CRDC–National Survey of Stroke
**Ives** [62], 1995	1989–1992	yes	USA (California, Maryland, North Carolina, Pennsylvania)	5,201	participants in the population-based Cardiovascular Health Study aged 65 years or older	ICD-9 inpatient and vital statistics records	CRMD
**Johnsen** [83], 2002	1993–1999	yes	Denmark	565	participants in a population-based cohort study on diet and cancer development aged 50–64 years at enrollment	ICD-10 inpatient records	CRDC—WHO criteria
**Jones** [84], 2014	1987–2010	yes	USA (Maryland, Minnesota, Mississippi, North Carolina)	4,260	members of the population-based Atherosclerosis Risk in Community (ARIC) Study cohort, aged 45–64 years at the time of study enrollment	ICD-9 inpatient records	CRDC–National Survey of Stroke, AHA/ASA
**Kirkman** [24], 2009	2002–2007	yes	United Kingdom	2,147	all hospitalized patients residing in the study area	ICD-10 inpatient records	chart review: mentioned in records
**Klatsky** [45], 2005	1978–1996	no	USA (California)	3,441	members of a prepaid healthcare program who supplied data on voluntary health examinations	ICD-9 inpatient records	CRMD
**Kokotalio** [85], 2005	2000–2003	yes	Canada (Alberta)	717	hospitalized patients at three centres	ICD-9 and ICD-10 inpatient records	chart review
**Koster** [60], 2013	2004	yes	Sweden	3,534	residents aged 20 years and older of two Swedish counties covered by the MONICA register	ICD-10 inpatient, outpatient, and vital statistics records	disease registry—MONICA
**Krarup** [86], 2007	1998–1999	yes	Denmark	236	enrollees in the population-based Copenhagen City Heart Study	ICD-10 inpatient records	CRDC—WHO criteria
**Kumamaru** [87], 2014	2003–2009	yes	USA (national)	15,089	participants in the REasons for Geographic And RacialDifferences in Stroke (REGARDS) study, aged ≥ 65 years with at least one month of Medicare eligibility	ICD-9 inpatient records	CRDC–WHO criteria
**Lakshminarayan** [46], 2009	1980, 1985, 1990, 1995, 2000	no	USA (Minnesota)	6,032	general population aged 30–74 years	ICD-9 inpatient records	CRDC–WHO and Minnesota Stroke Survey criteria, and neuroimaging
**Lakshminarayan** [88], 2014	1993–2007	yes	USA (national)	48,877	participants enrolled in the observational Women’s Health Initiative studies aged 50–79 years at enrollment with Medicare fee-for-service coverage	ICD-9 inpatient records	CRDC–Women's Health Initiative criteria
**Lambert** [89], 2012	2002–2006	yes	Canada (Quebec)	1,982	patients hospitalized with MI as a principal diagnosis, or who underwent PCI or CABG, at one of 13 primary, secondary, or tertiary hospitals	ICD-9 inpatient records	chart review; mentioned in records
**Lee** [90], 2005	1997–1999	yes	Canada (Ontario)	1,592	hospitalized individuals <105 years of age coded with a primary/most responsible diagnosis of heart failure	ICD-9 inpatient records	CRDC—Charlson comorbidity index
**Leibson** [91], 1999	1970, 1980, 1984, 1989	yes	USA (Minnesota)	377	all hospitalized patients residing in the study area	ICD-A and ICD-9 inpatient hospitalizations	disease registry—Rochester Stroke Registry
**Lentine** [92], 2009	1991–2002	yes	USA (Missouri)	571	kidney transplant recipients aged ≥ 18 years who had Medicare as their primary insurer at transplant and the time of each clinical event	ICD-9 inpatient records	clinical database
**Leone** [93], 2004	1998	yes	Italy	1,126	hospitalized patients from the Neurology, Neurosurgery, General Medicine, Cardiac Surgery, and Intensive Care departments at one centre	ICD-9 inpatient records	CRDC—WHO criteria
**Leppala** [51], 1999	1985–1989, 1992	yes	Finland	593	male smokers enrolled in a population-based, randomized controlled trial of alpha-tocopherol and beta-carotene supplementation, aged 50–69 years at registration	ICD-8 and ICD-9 inpatient and vital statistics records	CRDC—National Survey of Stroke and MONICA criteria
**Levy** [94], 1999	1994	yes	Canada (Quebec)	224	individuals aged ≥ 65 years discharged alive with a primary diagnosis of myocardial infarction	ICD-9 inpatient records	chart review: mentioned in records
**Lindblad** [53], 1993	1977–1987	yes	Sweden	413	participants in a hypertension registry (hypertensive cases, normotensive participants, randomly-selected controls), recruited from a geographical half of one Swedish county aged 40–70 years at registration	ICD-9 inpatient and vital statistics records	CRMD
**Liu** [26], 1999	1990–1991	yes	Canada (Saskatchewan)	1,494	patients hospitalized at one of three tertiary-care, or three community, hospitals	ICD-9 inpatient records	CRDC—National Survey of Stroke criteria
**Mayo** [95], 1993	n/a	yes	Canada (Quebec)	96	patients hospitalized at five teaching, affiliated, and community hospitals	ICD-9 inpatient records	CRMD
**Olson** [96], 2014	2001–2009	yes	USA (Colorado)	4,689	enrollees in a managed care organization aged 18 years and older	ICD-9 inpatient and outpatient records	CRDC–Rochester, Minnesota Stroke Study criteria
**Newton** [97], 1999	1992–1995	yes	USA (Washington)	471	enrollees of a Health Maintenance Organization with diabetes aged 18 years and older	ICD-9 inpatient and outpatient records	chart review
**Palmieri** [47], 2007	1997–1999	no	Italy	8,000	general population aged 35–74 years residing in one of eight regions of Italy	ICD-9 inpatient and vital statistics records	CRDC—MONICA criteria
**Phillips** [55], 1993	1988–1989	yes	Canada	301	patients hospitalized at one teaching hospital	ICD-9 inpatient and vital statistics records	CRDC–WHO criteria
**Piriyawat** [98], 2002	2000	yes	USA (Texas)	815	as part of the Brain Attack Surveillance in Corpus Christi (BASIC) Project, patients ≥ 45 years admitted to one of six area hospitals	ICD-9 inpatient records	CRDC–MONICA criteria
**Ramalle-Gomara** [99], 2013	2009	yes	Spain	400	patients hospitalized at one of two public hospitals	ICD-9 inpatient records	CRMD
**Rampatage** [61], 2013	2006–2008	yes	Sri Lanka	648	deaths occurring at three large hospitals in/near the capital city	ICD-10 vital statistics records	CRMD
**Rao** [56], 2007	2002	yes	China	2,917	deaths occurring in health facilities located in one of six large cities	ICD-10 vital statistics records	CRMD
**Reker** [100], 2001	1998–1999	yes	USA (national)	671	patients hospitalized at 11 Veterans Affairs Medical Centres	ICD-9 inpatient records	CRDC—WHO criteria
**Reggio** [64], 1995	1985–1992	yes	Italy	193	deaths occurring among residents of one municipality	ICD-9 vital statistics records	CRDC–WHO criteria
**Rinaldi** [23], 2003	1999	yes	Italy	233	hospitalized patients at one centre	ICD-9 inpatient records	prospective clinical examination and retrospective CRDC—WHO criteria
**Rosamond** [49], 1999	1987–1995	no	USA (Maryland, Minnesota, Mississippi, North Carolina)	1,185	members of the population-based Atherosclerosis Risk in Community (ARIC) Study cohort, aged 45–64 years at the time of study enrollment	ICD-9 inpatient records	CRDC–National Survey of Stroke
**Roumie** [101], 2008	1999–2003	yes	USA (Tennessee)	231	Medicaid enrollees aged 50–84 years, identified as part of a larger retrospective cohort study on the relationship between NSAID use and stroke	ICD-9 inpatient records	CRMD
**Shahar** [48], 1995	1980,1985, 1990	no	USA (Minnesota)	2,939	general population aged 30–74 years	ICD-9 inpatient records	CRDC—WHO, Minnesota Stroke Survey
**Singh** [102], 2012	2007–2009	yes	USA (Minnesota)	240	retrospective cohorts of patients ≥ 18 years of age admitted to the intensive care unit	ICD-9 inpatient and outpatient records	CRMD
**Sinha** [103], 2008	1993–2003	yes	United Kingdom	250	residents in one community aged 40–79 years and enrolled in a population-based study of the determinants of chronic disease	ICD-10 inpatient records	CRDC—WHO criteria
**So** [104], 2006	2003	yes	Canada (Alberta)	193	patients hospitalized for myocardial infarction	ICD-9 and -10 inpatient records	chart review
**Soo** [105], 2014	2003	yes	Scotland	3,219	participants in a population-based cohort study of chronic kidney disease	ICD-10 inpatient records	CRMD
**Spolaore** [106], 2005	1999	yes	Italy	4,015	general hospitalized population	ICD-9 inpatient records	CRDC—MONICA criteria
**Stegmayr** [58], 1992	1985–1989	yes	Sweden	6,000	residents of the two provinces included in the Northern Sweden MONICA study aged 25–74 years	ICD-9 inpatient and vital statistics records	disease registry—MONICA
**Szczesniewska** [59], 1990	1984–1986	yes	Poland	213	residents of two city districts covered by the POL-MONICA Warsaw Project aged 25–64 years	ICD-9 vital statistics records	disease registry—MONICA
**Thigpen** [107], 2015	2006–2010	yes	USA (Alabama, Massachusetts, Pennsylvania)	1,812	patients with atrial fibrillation hospitalized at one of three medical centres	ICD-9 inpatient records	CRDC–WHO, AHA
**Tirschwell** [108], 2002	1990–1996	yes	USA (Washington)	206	general hospitalized population ≥ 20 years of age	ICD-9 inpatient records	CRMD
**Tolonen** [52], 2007	1993–1998	yes	Finland	3,633	general population aged 25 years and older	ICD-9 and -10 inpatient and vital statistics records	disease registry—FINMONICA/FINSTROKE register
**Tu** [109], 2013	2011	yes	Canada	5,000	individuals aged ≥ 20 years seen by a family practice physician using the EMRALD EMR system	ICD-10 inpatient and outpatient records	CRMD
**Wahl** [110], 2010	2002–2004	yes	USA (national)	200	commercially-insured individuals in a large health claims database, identified as part of a larger retrospective observational cohort study on the risk of serious adverse events among users of selective coxibs and non-over-the-counter NSAIDs	ICD-9 inpatient records	CRMD
**Wildenschild** [111], 2014	2009–2010	yes	Denmark	228	Part 1: individuals ≥ 18 years admitted to hospital; Part 2: patients discharged from one of four neurologic wards	ICD-10 inpatient records	CRDC–WHO criteria
**Wu** [112], 2014	2004–2005	yes	Taiwan	15,574	individuals aged ≥ 12 years whose households were randomly selected for participation in the 2005 Taiwan National Health Interview Survey	ICD-9 inpatient and outpatient records	patient self-report

CRDC = Chart Review, Diagnostic Criteria–the charts of potential cases were reviewed, and a formal set of diagnostic criteria were applied when evaluating cases; CRMD = Chart Review, Medical Doctor–the charts of potential cases were reviewed by a physician, who evaluated cases using their clinical judgment or an otherwise unspecified set of criteria; AHA = American Heart Association; ASA = American Stroke Association; CABG = coronary artery bypass graft; EMRALD = Electronic Medical Record Administrative Data Linked Database; ICD = International Classification of Diseases; MONICA = MONItoring Trends and Determinants in CArdiovascular Disease; NSAID = non-steroid anti-inflammatory drug; PCI = percutaneous coronary intervention; TOAST = Trial of ORG 10172 in Acute Stroke Treatment; TIA = transient ischaemic attack; WHO = World Health Organization

### Gold standard

Chart reviews, sometimes in conjunction with unspecified diagnostic criteria, formed the basis of the gold standard in 35 studies, patient self-report was used in one [112], and national and regional stroke registries or clinical databases served as the gold standard in 12 [52,58–60,65,67,73,74,76,81,91,92]. One study [23] utilized two gold standards, with the reference diagnosis for some cases established upon prospective clinical examination by a neurologist, and for other cases, established after retrospective chart review by a different neurologist. The 28 remaining studies used a specific set of diagnostic criteria, most often the WHO criteria, to evaluate the stroke diagnosis.

Study quality was evaluated based on the QUADAS tool [38], with 54 of 77 studies (70%) categorized as high quality, and the remaining 23 studies as medium quality. A detailed breakdown of the quality assessment for each study is provided in **S1 Table**. Seven of the medium-quality studies [47,57,59,64,71,73,104] did not adequately describe the validation process or other key methodological aspects, while nine employed a selected study population [25,40,50,77,89,92,105,107,111] (e.g. atrial fibrillation cohort, kidney transplant recipients), and seven used a less-reliable gold standard [24,66,70,82,85,94,112], typically chart review by an individual other than a clinician or trained hospital coder.

### Validity of Stroke Codes on Aggregate

The validation statistics reported by each of the included studies are provided in **S2 and S3 Tables**. We located 36 papers examining the validity of the codes for cerebrovascular disease as an aggregate (ICD-9 430–438 or ICD-10 I60-I69); these codes were compared to diagnoses of any type of cerebrovascular disease (usually as a comorbidity) in 16 studies [56,61,63,66,71,77,79,80,82,89,90,93,94,102,104,105], and to a diagnosis of acute stroke in particular in 21 [26,41,45,47,49,52,54,55,57,62,64,74,83,84,86,91,93,99,103,111,112]. The sensitivity of these codes for any type of cerebrovascular disease was ≥ 82% in seven of the 14 studies (range 32% to 100%). The PPV was ≥ 81% in seven of the 14 studies reporting this statistic (range 43% to 97%). Specificity, reported by ten studies [63,66,71,79,82,89,90,102,104,105], was ≥ 95% in nine of the ten (range 90% to 100%), while NPV was ≥ 95% in eight of the ten studies [63,71,79,82,89,90,94,102,104,105] where this statistic was reported (range 84% to 100%). Kappa values, as reported by seven studies, ranged from 0.52 [82] to 0.76 [89] to 0.91 [79].

Eight of the 16 studies [56,61,63,79,80,90,93,102] were rated as high quality and the other eight [66,71,77,82,89,94,104,105] were rated as medium quality. There was little difference in the sensitivity values between the medium- and high-quality studies: sensitivity ranged from 43% to 100% among the medium-quality studies, and from 32% to 96% among the high-quality studies. There was, however, more of a difference in the PPVs, which ranged from 43% to 83% among the medium-quality studies, and from 52% to 97% among the high-quality studies (with PPV ≥ 88% in five of the seven high-quality studies reporting on PPV).

The sensitivity of these codes for the narrower diagnosis of acute stroke (SAH, ICH, or ischaemic stroke), which was reported by ten studies [52,54,57,62,64,74,93,99,111,112], was ≥ 66% in seven of the ten (range 42% to 96%). The PPV for the reference diagnosis of acute stroke was generally lower than that for the broader category of any cerebrovascular disease, being ≤ 68% in 12 of the 21 studies reporting this statistic (range 28% to 98%). Indeed, in one study that evaluated the PPV against both acute stroke and ‘any cerebrovascular disease’, the PPV for ‘any cerebrovascular disease’ (93%) was higher than that for acute stroke (66%) [93].

### Subarachnoid Haemorrhage

Twenty-seven papers reported on the validity of codes for SAH (ICD-9 430 or ICD-10 I60), and the PPV was ≥ 86% in 16 of the 26 studies where this was reported (**S2 Table**). Fifteen papers compared the SAH code to any type of acute stroke (meaning a case coded for SAH was considered a true-positive if the diagnosis assigned upon validation was SAH, ICH, or ischaemic stroke), and the PPV was ≥ 86% in eight of these studies (range 33% to 100%). Thirteen studies compared the SAH code to the diagnosis of SAH in particular, and the PPV was ≥ 93% in seven of the 13 (range 46% to 100%). The sensitivity of these codes for SAH, reported by only four studies [52,56,84,93], ranged from 35% [93] to 95% [52]. Kappa, reported by only one study [108], was 0.88.

### Intracerebral Haemorrhage

Thirty-four studies evaluated the validity of codes for ICH (ICD-9 431/432 or ICD-10 I61/62) (**S2 Table**). Twenty-six evaluated the main ICH codes 431/I61, and the PPV was ≥ 87% in 16 of the 25 studies reporting on PPV. The PPV, when compared to any type of acute stroke, was ≥ 87% in ten of 15 studies (range 78% to 100%), and when compared to the diagnosis of ICH in particular, the PPV was ≥ 89% in six of the 12 studies (range 63% to 100%). The sensitivity of these codes for ICH, as reported by three studies, ranged from 57% [93] to 69% [56] to 95% [52]. The kappa value for ICD-9 431, as reported by one study [108], was 0.82, while that for ICD-9 430 and 431 combined, was 0.84 [88]. The lesser-used ICH codes (ICD-9 432 or ICD-10 I62) were evaluated in 15 studies [26,41,47,49,55,62,74,78,84,91,93,95,96,100,106], in which the PPV was ≤ 67% in all but two [62,95]. The PPV of ICD-9 431 and 432 combined, available from 13 studies [26,41,42,47,49,53,55,57,74,84,91,93,95], ranged from 33% to 99%. In sum, the PPV of the main codes for ICH (ICD-9 431/ICD-10 I61) was ≥ 87% in most studies.

### Ischaemic Stroke

We located 39 papers that examined the validity of codes for ischaemic stroke (**S2 Table**). From these, 28 evaluated the main code for ischaemic stroke (ICD-9 434 or ICD-10 I63) and the PPV was ≥ 82% in 20 of the 27 studies reporting this statistics (range 62% to 100%). The PPV was ≥ 83% in 13 of the 19 papers [26,41,42,47,49,50,53,55,62,69,74,78,84,91,93,95,96,100,106] where the gold standard diagnosis was any type of acute stroke (SAH, ICH, or ischaemic stroke) (range 62% to 100%), and ≥ 82% in 10 of the 13 papers [23,25,49,50,52,53,76,83,84,86,93,96,107] where the gold standard diagnosis was iscahemic stroke in particular (range 52% to 100%). The sensitivity of these codes for ischaemic stroke, available from six papers [23,52,56,76,84,93], ranged from 2% [23] to 80% [52]. ICD-9 433 was evaluated in 19 papers [25,26,40,41,47,49,50,53,55,69,74,84,91,93,95,96,100,106,107], with the PPV being ≤ 71% in 14 of the 19. Eight papers [25,49,50,53,84,93,96,107] reported on the PPV of ICD-9 433 for ischaemic stroke in particular, and it was ≤ 79% in six of the eight. Just two studies reported on the sensitivity of ICD-9 433 for ischaemic stroke, which was 2% [93] in one and 9% [84] in the other.

The combination of ICD-9 433 and 434 (occlusion of the precerebral or cerebral arteries) was reported on by 20 studies [26,40,41,47,49,51–53,55,57,69,72,74,81,84,93,95,99,107,110], in which the PPV for any stroke ranged from 23% to 100%, and that for ischaemic stroke ranged from 40% to 100%. The sensitivity of these codes for ischaemic stroke, as reported by five studies [52,81,84,93,99], was ≥ 76% in four of these. The code for acute but ill-defined stroke (ICD-9 436 or ICD-10 I64) was evaluated in 23 studies [23,25,26,40,41,47,49,50,53,55,62,69,74,78,83,84,91,93,95,96,100,106,107], with a PPV for any stroke of ≥ 75% in 12 of 19 studies (range 48% to 95%) and a PPV for ischaemic stroke of ≥ 75% in five of seven studies [23,25,49,50,84,96,107] where this statistic was reported (range 50% to 87%).

Nineteen studies [25,26,40,41,47,49,52,55,69,74,84,85,87,88,93,95,101,107,108] employed a broader case definition (ICD-9 433/434/436 or ICD-10 I63/64); the PPV was ≥ 77% in 12 of the 19 (range 46% to 94%). Two studies reported on kappa, which, for ICD-9 433, 434, and 436 together, was 0.82 [108] in one study and 0.85 [88] in the other. Sixteen studies [23,26,40,41,47,49,53,55,62,69,74,78,84,93,95,107] examined the validity of ICD-9 434 and 436 as a pair, and the PPV was ≥ 82% in ten of the 16 (range 66% to 94%). Of interest, Leone *et al* [93] compared the sensitivity and PPV of different codes for ischaemic stroke, and found that ICD-9 434 and 436 combined had a higher sensitivity (43% versus 35%) for ischaemic stroke, but similar PPV (87% versus 90%), than did ICD-9 434 alone. In sum, the PPV of the main codes for ischaemic stroke (ICD-9 434/ICD-10 I63) was ≥ 82% in most studies, and the PPV of codes for acute but ill-defined stroke (ICD-9 436 or ICD-10 I64) was ≥ 75%.

### Validity of Sets of Stroke-Specific Codes

Thirty-six papers examined the validity of a set of stroke-specific codes for identifying any type of stroke (**S3 Table**) though in two [70,92], the reference diagnosis was stroke/TIA and not just acute stroke. There was some variability in the codes included in each set, but they generally excluded codes pertaining to occlusion of the precerebral arteries, intracranial haemorrhage other than ICH, late effects of stroke/stroke sequalae, and cerebrovascular disorders stemming from other conditions. The code for acute but ill-defined stroke was included in some of these sets. One study, by Reker *et al* [100], used both a high-sensitivity algorithm for detecting stroke (sensitivity = 91%, PPV = 52%) and high-specificity algorithm (sensitivity = 54%, PPV = 75%). Lakshminarayan *et al* [46] compared two diagnostic criteria, those from the WHO and Minnesota Stroke Survey (MSS), and found the PPV against the WHO criteria was 98%, while that against the stricter MSS criteria was 68% [46].

The sensitivity of these sets of codes for stroke was ≥ 82% in 13 of 22 studies [40,41,59,62,65,67,70,73–75,78,87,88,92,93,97–101,109,111] where this was reported (range 34% to 97%), and the PPV was ≥ 86% in 18 of the 34 studies [40,41,44,46–48,51,53,58–60,62,65,67,68,70,73–75,78,85,87,88,91,93,95,97–101,107,109,111] that reported this statistic (range 32% to 98%). Amongst these 34 studies, we observed a tendency towards lower PPV when codes for intracranial haemorrhage (ICD-9 432) or ill-defined stroke (ICD-9 437) were included in the search (**S3 Table**). Kappa ranged from 0.79 [108] to 0.81 [78] to 0.87 [88] in the three studies where this was reported.

### Validity by Subgroups

The 77 studies included in this review were published over a 40-year period (1976–2015), though 81% of these (n = 62) were published from 1999-onwards. Few studies reported on any longitudinal changes in sensitivity, and few longitudinal trends in PPV were observed after the 77 studies were stratified by period of publication. For instance, amongst the 27 studies reporting on the PPV of ICD-9 434/ICD-10 I63, the PPV ranged from 64% to 100% in the eight-earliest studies (published from 1993 to 1998), from 72% to 100% in the ten middle studies (published from 1999 to 2004), and from 62% to 100% in the nine most-recent studies (published from 2005 to 2015). And among the 26 studies reporting on ICD-9 431/ICD-10 I61, the PPV ranged from 66% to 100% in the 12 earlier studies (published from 1993–2002) and from 65% to 100% in the 13 more-recent studies (published from 2004–2014). Still, several investigators collected data over ten or more years, and some improvements were observed in the PPV for stroke over time. In one study [91], the PPV of ICD 430–438 increased from 48% to 58% between 1970 and 1980, though by 1989 it had decreased slightly, to 54%. Moreover, Derby *et al* [42] found that the PPV of ICD-9 431, 432, 434, 435, 436, or 437 for stroke increased by 20% between 1980 and 1990, while Lakshminarayan *et al* [46] found that the PPV of ICD-9 431, 432, 434, 436, and 437 for stroke increased in this same period by 27% (from 55% in 1980 to 70% in 1990). These studies provide evidence for the accuracy of codes for acute stroke having improved over time.

The accuracy of fatal and non-fatal stroke diagnoses were compared in nine studies [47,51–53,58,60,62,65,91], and amongst these, the accuracy of fatal diagnoses was similar, and often slightly higher, than non-fatal diagnoses (**S4 Table**). Fifteen studies examined cerebrovascular disease or acute stroke as a cause-of-death on death certificates, and the PPV was ≥ 87% in ten of these (range 50% to 100%). However, the sensitivity of vital statistics data for deaths from stroke was ≤ 71% in six of the 10 studies reporting on this (range 32% to 96%), and the sensitivity of hospitalization data for fatal strokes—in the single study where this statistic was reported—was 55% (compared to 68% for non-fatal hospitalizations for stroke) [91].

Most studies examined codes from the ICD 8^th^ and 9^th^ revisions, though 22 studies examined codes from the 10^th^ revision. Just one of these studies [54] was conducted in the United States. Separate validation statistics for ICD-9 and ICD-10 codes were provided by three articles [52,85,104], and, overall, there were few differences in the accuracy of codes from the two revisions. One study was conducted exclusively on males [51], and two were conducted exclusively on females [78,88], and their findings were consistent with those of most studies where both sexes were represented. Sex-stratified statistics were provided by six studies [47,52,84,87,93,105], from which only minor differences in the accuracy of codes for males and females were observed.

## Discussion

In performing what is (to our knowledge) the broadest systematic review ever conducted on the validity of stroke diagnoses in administrative data, we observed high PPVs for codes pertaining to the different subtypes of acute stroke. The PPV of SAH codes for an SAH diagnosis was ≥ 93% in most studies, that for the main ischaemic stroke codes was ≥ 82%, and the PPV of the main ICH codes for an ICH diagnosis was ≥ 89%. For diagnoses of fatal stroke, the PPV was ≥ 87% in most studies. The validity of the group of ICD codes corresponding to cerebrovascular disease in general (ICD-9 430–438 and ICD-10 I60-69) was also generally good; sensitivity was ≥ 82% in half the studies where this was reported, specificity and NPV were ≥ 95%, and the PPV of these codes against the broader reference standard of ‘any cerebrovascular disease’ was ≥ 81% in most studies. However, the PPV was lower (≤ 68% in 12 of 21 studies) when the reference standard was restricted to acute stroke (defined as SAH, ICH, or ischaemic stroke). Given these findings, we conclude that most diagnoses of fatal stroke in administrative data correspond to true stroke deaths, and that the presence of any code from 430–438 or I60-I69 can be used to rule-in the diagnosis of prevalent cerebrovascular disease. We also conclude that administrative data can be used to identify cases of acute stroke, as long as extraneous codes (i.e. ICD-9 432, 435, 437, and 438; ICD-10 I62, I67, I68, and I69) are excluded.

Only a few studies evaluated the sensitivity of individual codes for stroke but from these, it appears the sensitivity of the main ICD-9 code for ischaemic stroke (434) is suboptimal. However, findings from some studies included in this review suggest that adding ICD-9 code 433 (occlusion of precerebral arteries), and/or 436 (acute but ill-defined stroke) to the search algorithm can help capture more cases of ischaemic stroke at little cost to the PPV. Further support is provided by the fact that the PPV of ICD-9 436 for ischaemic stroke in most applicable studies was ≥ 75%. Cases of ischaemic stroke appear to be coded as “acute but ill-defined stroke” much more often than haemorrhagic strokes are. For example, of all strokes that were coded initially as ill-defined, Krarup *et al* [86] re-classified 57% of these as ischaemic, and just 6% as haemorrhagic. We believe this is because it is harder to make a conclusive diagnosis of ischaemic stroke: neuroimaging can identify bleeds and haemorrhagic lesions more easily than brain infarction, especially within the first twelve hours of onset [35]. Despite the WHO [36] and other criteria calling for cases that fulfill the clinical criteria of stroke, but whose CT is negative for recent brain lesions, to be classified as ischaemic stroke (ICD-9 433 or 434), conservative clinicians and coders may still be inclined to classify these as acute, but ill-defined stroke (ICD-9 436).

The findings of our review are consistent with those of a systematic review of algorithms for identifying acute stroke or TIA in administrative data that was published in 2012 [113]. That review, conducted as part of the US Food and Drug Administration’s Mini-Sentinel Program, had a limited scope compared with ours, as it was restricted to evaluations of US and Canadian databases published from 1990-onwards. Still, consistent with our findings, that review found that when individual ICD-9 stroke codes were examined, the PPV’s were highest amongst 430, 431, and 434, and much lower when any other code from 430–438 was included in the search algorithm. Further, the authors recommend that codes 433.x1, 434 (excluding 434.x0), and 436 be used when searching for cases of acute ischaemic stroke [113]. They also suggest that when using administrative data to evaluate drug safety, the outcome of interest should be a definite subtype of stroke rather than the broader endpoint of ‘any cerebrovascular disease’ as defined by ICD-9 430–438 [113].

### Regional and Temporal Trends

The PPV of diagnostic codes for stroke appears to have improved over the decades, increasing by 20% in one study (where about 51% of stroke discharges identified in 1980, and about 61% of stroke discharges identified in 1990, were confirmed as stroke) [42], and by 27% in another [46] (from 55% in 1980 to 70% in 1990). Many papers included in this review [42,46,53,93,103] attribute these improvements to advances in neuroimaging technology, and increased use and availability of CT and MRI scanners in medical facilities. For example, the 20% increase in PPV reported by Derby *et al* [42] (in the northeastern US) was accompanied by a 120% increase in the proportion of potential cases who underwent CT or MRI, and a 60% increase in the proportion seen by a neurologist. CT rates have increased in other countries as well, in Finland from 18% in 1983 to 60% in 1989 [114], and in Sweden from 88% in 1995 to 98% in 2010 [115]. In fact, the introduction of CT scanning in the 1970’s is thought to have improved not only the detection of acute strokes, but also the ability of clinicians to make a more precise diagnosis [53]. For instance, when Lindblad *et al* [53] retrospectively reviewed the stroke codes assigned to cases over 1977–1987, the subtype was reclassified, often on the basis of CT findings, in 27% of the confirmed stroke cases [53]. More strokes were classified as thromboembolic, and fewer as ill-defined, over time in the Derby *et al* study [42], and similar trends have been reported in Denmark, where the incidence of unspecified stroke decreased from 1997 to 2009, while the incidence of ischaemic stroke increased [116].

Regional differences in access-to and use-of neuroimaging may explain other disparities that were observed in the validity of strokes diagnoses, even amongst studies conducted more recently (i.e. 1990 or later). Tolonen *et al* [52] observed that those aged 75 years and older were coded more often with non-specific stroke codes. They attributed this to older individuals being less likely to undergo CT and MRI, which could otherwise aide in making a more precise diagnosis. They also found that the sensitivity and PPV were lower at district hospitals than university hospitals [52], and attributed this to the limited neurological expertise and neuroimaging available at the district hospitals. Liu *et al* [26] also observed higher PPV’s amongst the tertiary hospitals than community hospitals, and attributed this to the greater level of testing performed in the tertiary hospitals. As neuroimaging services continue to become more widespread, and more technologies like CT angiography and perfusion imaging [117] emerge, we expect the validity of stroke codes to increase alongside.

It is possible that changes in billing and reimbursement practices, including the introduction of Diagnosis-Related Groups (DRGs) in the US Medicare program in the 1980’s, may also have contributed to the assignment of more precise stroke codes over time. To investigate this, Derby *et al* [118] compared how strokes were classified before and after DRGs were introduced, but their findings (increased use of ICD-9 434 and decreased use of ICD-9 436) did not directly correspond with any financial incentives. In addition, while the Medicare DRG system was implemented only in the US, this temporal trend was observed in other countries [115,116] as well.

In our systematic review of the validity of myocardial infarction (MI) diagnoses in administrative data [32], we observed that the accuracy of MI as a cause-of-death on death certificates was generally lower than hospital discharge diagnoses. In contrast, findings from this review suggest that diagnoses of fatal stroke are as accurate, if not more accurate, than diagnoses of non-fatal stroke. One article included in this review, by Tolonen *et al* [52], suggests this is because autopsies can provide additional information that improves the accuracy of the diagnosis. In that study, the sensitivity and PPV for SAH and ICH were markedly high (sensitivity 100% and 90%, and PPV 100% and 100%, for SAH and ICH, respectively) amongst cases diagnosed from outpatient clinics, and all of these were fatal cases that were autopsied [52]. With these findings, researchers should feel confident that most diagnoses of fatal stroke in administrative data correspond to true stroke deaths. However, our findings do suggest that, individually, these databases have suboptimal sensitivity for detecting fatal stroke. In one paper, 79% of deaths that were listed in the mortality register as being from unspecified cerebrovascular disease were re-classified by the investigating physician as deaths from acute stroke [53]. In another paper, only 21% of confirmed stroke deaths in the vital statistics database appeared in the hospitalization database [51]. Thus, when investigating fatal stroke, researchers could improve the ascertainment of cases by linking vital statistics data with hospitalization data, and attributing to stroke any deaths occurring within a certain period (i.e. 28 days, as used in the WHO MONICA Project [36]) of a hospitalization coded for stroke. Sensitivity analyses investigating the impact of longer periods of time (i.e. 90 days) on case ascertainment should be conducted alongside.

### Consequences of Using Broad Sets of Cerebrovascular Disease Codes

Many of the studies that reported on the validity of ICD-9 codes 430–438, or ICD-10 codes I60-69, as a group were examining how well these codes performed for detecting preexisting cerebrovascular disease as a comorbidity. Searching for any one of the codes in this group is appropriate when seeking to identify and adjust for preexisting cerebrovascular disease in the analyses of other clinical conditions. The high sensitivity we observed for these codes, and high PPV they had when compared to the reference diagnosis of ‘any cerebrovascular disease’, provides additional support for this use. However, when studying acute stroke as a primary outcome, this broad group of codes, which includes the codes for non-acute and ill-defined cerebrovascular disease, and the late effects of stroke, should not be used. It is far more difficult to identify risk factors for stroke from this mixture of recent-onset and prevalent cerebrovascular disease because it is unclear which characteristics may have increased the risk of *developing* stroke, versus *surviving* the stroke. Instead, in pharmacoepidemiologic studies and other analyses of risk factors where acute stroke is the primary outcome of interest, and diagnostic specificity is of upmost importance, only stroke-specific codes should be used.

### Limitations

We acknowledge some limitations to our systematic review. There is the potential for a language bias as we could not consider articles whose full-text was not available in English. We were also conservative in our definition of acute stroke, and excluded studies that only reported on the validity of diagnostic codes for TIA. Another potential limitation stems from the fact that, even though our database searches were conducted by an experienced librarian, administrative databases are not well catalogued in MEDLINE and EMBASE (e.g. no MeSH term pertaining to “administrative database”). Although the majority of the included studies were located through database searches, our subsequent hand search turned up other relevant articles that had not been indexed under terms relating to Administrative Data or Validation. As a result, despite our extensive hand search, we may have missed some relevant articles if they were not indexed in MEDLINE or EMBASE under a term relating to administrative data or validation. Our findings are also subject to publication bias, wherein reports of stroke codes having poor validity may have been differentially withheld from publication. We feel this is unlikely, however, given that we did locate reports of case definitions (i.e. ICD-9 432 or 433 individually) whose sensitivity and PPV for acute stroke were suboptimal.

### Conclusions and Recommendations

Following our analysis of the evidence, we conclude that the diagnostic codes for acute stroke in administrative databases are valid. In fact, advances in neuroimaging and the increased availability of CT scanners may have helped improve diagnosis and coding of acute stroke subtypes over time. However, it is apparent that researchers have been using a variety of codes to identify acute stroke, some of which have suboptimal validity. Based on current evidence, we provide researchers with several recommendations for the use of diagnostic codes to capture cases of stroke in administrative data. We believe the findings of our review will help guide researchers in their efforts to better understand and decrease the burden of stroke.

1. As a group, the range of codes for cerebrovascular disease (ICD-9 430-438/ICD-10 I60-I69) has good sensitivity (≥ 82%), specificity (≥ 95%), and PPV (≥ 81%) for identifying the aggregate diagnosis of acute or preexisting cerebrovascular disease.
2. Codes that pertain to the diagnosis of acute stroke (ICD-9 430/ICD-10 I60, ICD-9 431/ICD-10 I61, ICD-9 434/I63, and ICD-9 436/ICD-10 I64) are highly predictive of true cases of acute stroke of any type, and of the particular subtype.
  ⚬ These are the codes that should be used when identifying acute stroke as an outcome, especially in pharmacoepidemiologic and other analyses of risk factors where diagnostic specificity is essential.
3. When searching for cases of ischaemic stroke, including both the code for ischaemic stroke (ICD-9 434/ICD-10 I63) and the code for acute-but-ill-defined stroke (ICD-9 463/ICD-10 I64) in the case definition should help capture more cases of ischaemic stroke with little impact on the PPV.
4. Whether identified from hospitalization or vital statistics data, diagnoses of fatal stroke generally correspond to true deaths from stroke.
5. Hospitalization and vital statistics databases should be linked and searched together in order to maximize the capture of stroke deaths.

## Supporting Information

S1 Checklist PRISMA Checklist. (DOC)

S1 Text MEDLINE search strategy (inception to November 2010). (DOCX)

S2 Text EMBASE search strategy (inception to November 2010). (DOCX)

S3 Text MEDLINE search strategy (January 2010 to February 2015). (DOCX)

S4 Text EMBASE search strategy (January 2010 to February 2015). (DOCX)

S5 Text Data Collection Form. (DOC)

S1 Table Item-by-Item QUADAS Breakdown for Each Study. (DOCX)

S2 Table Results of Studies Validating Diagnoses of Stroke in Administrative Data. (DOC)

S3 Table Results of Studies Validating Sets of Diagnostic Codes for Stroke in Administrative Data. (DOC)

S4 Table Results of Studies Validating Diagnoses of Fatal Stroke in Administrative Data. (DOCX)
