# Supplementary material for: Validity of Diagnostic Codes for Acute Stroke in Administrative Databases: A Systematic Review
Source: PLoS One. 2015 Aug 20;10(8):e0135834. doi: 10.1371/journal.pone.0135834 (PMC4546158; doi:10.1371/journal.pone.0135834)
Supplement: S3 Text — (DOCX) [file pone.0135834.s004.docx]

**S3 Text. MEDLINE search strategy (January 2010 to February 2015).**

Database: Ovid MEDLINE(R) In-Process & Other Non-Indexed Citations and Ovid MEDLINE(R) <1946 to Present>

Search Strategy:

--------------------------------------------------------------------------------

1 administrative data.ti,ab. (4184)

2 administrative database:.ti,ab. (2403)

3 Databases, Factual/ (45491)

4 factual database/ (45491)

5 Databases as Topic/ (8092)

6 database/ (0)

7 Medical Record Linkage/ (3635)

8 administrative databank:.ti,ab. (2)

9 factual database:.ti,ab. (16)

10 factual databank:.ti,ab. (2)

11 factual data.ti,ab. (73)

12 exp medical records/ (87934)

13 exp medical record/ (87934)

14 exp medical records systems, computerized/ (26300)

15 (medical record or health record or medical records or health records).ti,ab. (70634)

16 medical transcription:.ti,ab. (82)

17 exp Registries/ (59413)

18 registry/ (55640)

19 (registry or registries).ti,ab. (66319)

20 (utilization data: or utilisation data: or claims data: or managed care data: or physician billing data: or hospitalization data: or linked data:).ti,ab. (9103)

21 (administrative healthcare data: or administrative health care data: or administrative health data: or administrative health data:).ti,ab. (346)

22 (medical records based index or claims based index).ti,ab. (6)

23 (register and (link or links or linked or linkage or linking)).ti,ab. (2753)

24 or/1-23 [ADMINISTRATIVE DATA (BROAD)] (294500)

25 Validation Studies/ (70184)

26 validation study/ (0)

27 Validation Studies as Topic/ (1472)

28 Validation Studies.pt. (70184)

29 (validat: or validity).ti,ab. (370819)

30 or/25-29 [VALIDATION STUDIES] (396612)

31 or/1-2,8,21 [ADMINISTRATIVE DATA (NARROWEST)] (6583)

32 Coronary Artery Disease/ (39402)

33 coronary artery disease/ (39402)

34 coronary artery disease:.ti,ab. (61991)

35 Myocardial Infarction/ (142413)

36 acute heart infarction/ (0)

37 acute myocardial infarction:.ti,ab. (47055)

38 exp Heart Failure/ (88785)

39 exp congestive heart failure/ (88785)

40 congestive heart failure.ti,ab. (31999)

41 exp Stroke/ (88693)

42 stroke/ (61871)

43 ((stroke or strokes) and (brain or cerebral or cerebrovascular)).ti,ab. (47750)

44 Brain Ischemia/ (37314)

45 brain ischemia/ (37314)

46 ((brain or cerebral or cerebrovascular) adj2 (vascular accident: or apoplex: or infarction: or ischemi:)).ti,ab. (44621)

47 (cerebrovascular event or cerebrovascular events).ti,ab. (3226)

48 or/32-47 [CARDIOVASCULAR (SPECIFIC)] (458844)

49 24 and 30 and 48 [ADMINISTRATIVE DATA (BROAD) + VALIDATION STUDIES + CARDIOVASCULAR (SPECIFIC)] (939)

50 limit 49 to yr="2010 - 2015" (473)

51 31 and 48 [ADMINISTRATIVE DATA (NARROWEST) + CARDIOVASCULAR (SPECIFIC)] (640)

52 limit 51 to yr="2010 - 2015" (295)

53 50 or 52 (724)

54 52 not 50 (251)

55 remove duplicates from 50 (464)

56 from 55 keep 1-464 (464) SAVED EN 5617-6018 (402 UNIQUE)

57 remove duplicates from 54 (245) SAVED EN 6019-6224 (211 UNIQUE)
