# Supplementary material for: Validity of Diagnostic Codes for Acute Stroke in Administrative Databases: A Systematic Review
Source: PLoS One. 2015 Aug 20;10(8):e0135834. doi: 10.1371/journal.pone.0135834 (PMC4546158; doi:10.1371/journal.pone.0135834)
Supplement: S4 Text — (DOCX) [file pone.0135834.s005.docx]

**S4 Text. EMBASE search strategy (January 2010 to February 2015).**

Database: Embase <1974 to 2015 Week 07>, Ovid MEDLINE(R) In-Process & Other Non-Indexed Citations, Ovid MEDLINE(R) Daily, Ovid MEDLINE(R) and Ovid OLDMEDLINE(R) <1946 to Present>

Search Strategy:

--------------------------------------------------------------------------------

1 administrative data.ti,ab. (9603)

2 administrative database:.ti,ab. (6028)

3 Databases, Factual/ (60785)

4 factual database/ (60785)

5 Databases as Topic/ (169022)

6 database/ (160929)

7 Medical Record Linkage/ (127918)

8 administrative databank:.ti,ab. (5)

9 factual database:.ti,ab. (40)

10 factual databank:.ti,ab. (6)

11 factual data.ti,ab. (174)

12 exp medical records/ (237313)

13 exp medical record/ (237313)

14 exp medical records systems, computerized/ (53313)

15 (medical record or health record or medical records or health records).ti,ab. (173375)

16 medical transcription:.ti,ab. (177)

17 exp Registries/ (131338)

18 registry/ (127562)

19 (registry or registries).ti,ab. (168466)

20 (utilization data: or utilisation data: or claims data: or managed care data: or physician billing data: or hospitalization data: or linked data:).ti,ab. (23844)

21 (administrative healthcare data: or administrative health care data: or administrative health data: or administrative health data:).ti,ab. (868)

22 (medical records based index or claims based index).ti,ab. (21)

23 (register and (link or links or linked or linkage or linking)).ti,ab. (6358)

24 or/1-23 [ADMINISTRATIVE DATA (BROAD)] (779392)

25 Validation Studies/ (116250)

26 validation study/ (45934)

27 Validation Studies as Topic/ (47409)

28 Validation Studies.pt. (70316)

29 (validat: or validity).ti,ab. (862654)

30 or/25-29 [VALIDATION STUDIES] (899313)

31 or/1-2,8,21 [ADMINISTRATIVE DATA (NARROWEST)] (15623)

32 Coronary Artery Disease/ (193667)

33 coronary artery disease/ (193667)

34 coronary artery disease:.ti,ab. (151169)

35 Myocardial Infarction/ (360348)

36 acute heart infarction/ (51789)

37 acute myocardial infarction:.ti,ab. (111067)

38 exp Heart Failure/ (410924)

39 exp congestive heart failure/ (167280)

40 congestive heart failure.ti,ab. (73765)

41 exp Stroke/ (185635)

42 stroke/ (186964)

43 ((stroke or strokes) and (brain or cerebral or cerebrovascular)).ti,ab. (118356)

44 Brain Ischemia/ (121466)

45 brain ischemia/ (121466)

46 ((brain or cerebral or cerebrovascular) adj2 (vascular accident: or apoplex: or infarction: or ischemi:)).ti,ab. (104015)

47 (cerebrovascular event or cerebrovascular events).ti,ab. (8304)

48 or/32-47 [CARDIOVASCULAR (SPECIFIC)] (1387709)

49 24 and 30 and 48 [ADMINISTRATIVE DATA (BROAD) + VALIDATION STUDIES + CARDIOVASCULAR (SPECIFIC)] (3362)

50 limit 49 to yr="2010 - 2015" (2187)

51 31 and 48 [ADMINISTRATIVE DATA (NARROWEST) + CARDIOVASCULAR (SPECIFIC)] (1976)

52 limit 51 to yr="2010 - 2015" (1142)

53 50 or 52 (3153)

54 52 not 50 (966)

55 remove duplicates from 50 (1862)

**56 from 55 keep 1-1397 (1397) SAVED EN 6230-7381 (1152 UNIQUE)**

57 remove duplicates from 54 (738)

58 from 56 keep 1001-1396 (396)

**59 from 57 keep 1-493 (493) SAVED EN 7382-7776 (395 UNIQUE)**
