# Supplementary material for: Validity of Diagnostic Codes for Acute Stroke in Administrative Databases: A Systematic Review
Source: PLoS One. 2015 Aug 20;10(8):e0135834. doi: 10.1371/journal.pone.0135834 (PMC4546158; doi:10.1371/journal.pone.0135834)
Supplement: S1 Table — (DOCX) [file pone.0135834.s007.docx]

**S1 Table. Item-by-Item QUADAS Breakdown for Each Study.**

| **QUADAS Question no.** | **Aboa-Eboule[67]** | **Agrawal[40]** | **Appelros[65]** | **Arnason[68]** | **Benesch[69]** | **Birman-Deych[70]** | **Borzecki[66]** | **Broderick[41]** | **Brown[54]** | **Chen[71]** |
| --- | --- | --- | --- | --- | --- | --- | --- | --- | --- | --- |
| **1** | Y | N | Y | Y | Y | N | N | Y | Y | Y |
| **2** | Y | Y | Y | Y | Y | Y | Y | Y | Y | Y |
| **3** | Y | Y | Y | U | Y | N | N | Y | Y | U |
| **4** | NA | NA | NA | NA | NA | NA | NA | NA | NA | NA |
| **5** | Y | Y | Y | Y | Y | Y | Y | Y | Y | Y |
| **6** | Y | Y | Y | Y | Y | Y | Y | Y | Y | Y |
| **7** | U | Y | N | Y | N | Y | Y | N | N | Y |
| **8** | Y | Y | Y | Y | Y | Y | Y | Y | Y | Y |
| **9** | N | Y | Y | Y | Y | U | Y | Y | Y | N |
| **10** | Y | Y | Y | Y | Y | Y | Y | Y | Y | Y |
| **11** | Y | U | NA | Y | U | Y | U | Y | Y | U |
| **12** | Y | Y | Y | Y | Y | Y | Y | Y | Y | Y |
| **13** | Y | Y | Y | Y | Y | Y | N | N | Y | NA |
| **14** | NA | NA | NA | NA | NA | NA | NA | NA | NA | NA |
| **Overall Quality Assessment** | High | Medium | High | High | High | Medium | Medium | High | High | Medium |

Y=Yes; N=No; U=Unclear; NA=Not Applicable

| **QUADAS Question no.** | **Cheng[72]** | **Davenport[73]** | **de Faire[63]** | **Derby[42]** | **Ellekjaer[74]** | **Gaist[43]** | **Ghia[75]** | **Goldstein[25]** | **Golomb[50]** | **Haesebaert[76]** |
| --- | --- | --- | --- | --- | --- | --- | --- | --- | --- | --- |
| **1** | N | Y | Y | Y | Y | Y | Y | N | N | Y |
| **2** | Y | Y | Y | Y | Y | Y | Y | Y | Y | Y |
| **3** | Y | Y | Y | U | Y | Y | Y | Y | Y | Y |
| **4** | NA | NA | NA | NA | NA | NA | NA | NA | NA | NA |
| **5** | Y | Y | Y | Y | Y | Y | Y | Y | Y | Y |
| **6** | Y | Y | Y | Y | Y | Y | Y | Y | Y | Y |
| **7** | N | N | Y | N | N | N | N | N | N | Y |
| **8** | Y | Y | Y | Y | Y | Y | Y | Y | Y | Y |
| **9** | Y | U | Y | Y | Y | Y | Y | Y | Y | Y |
| **10** | Y | Y | Y | Y | Y | Y | Y | Y | Y | Y |
| **11** | U | Y | Y | U | U | Y | U | U | U | Y |
| **12** | Y | U | Y | Y | Y | Y | Y | Y | Y | Y |
| **13** | Y | Y | Y | Y | Y | Y | Y | Y | Y | NA |
| **14** | NA | NA | NA | NA | NA | NA | NA | NA | NA | NA |
| **Overall Quality Assessment** | High | Medium | High | High | High | High | High | Medium | Medium | High |

Y=Yes; N=No; U=Unclear; NA=Not Applicable

| **QUADAS Question no.** | **Hasan[77]** | **Heckbert[78]** | **Henderson[79]** | **Hennessy[80]** | **Holick[44]** | **Hsieh[81]** | **Humphries[82]** | **Iso[57]** | **Ives[62]** | **Johnsen[83]** |
| --- | --- | --- | --- | --- | --- | --- | --- | --- | --- | --- |
| **1** | N | N | Y | Y | Y | Y | N | Y | N | Y |
| **2** | Y | Y | Y | Y | Y | Y | Y | Y | Y | Y |
| **3** | Y | Y | U | Y | Y | Y | N | Y | Y | Y |
| **4** | NA | NA | NA | NA | NA | NA | NA | NA | NA | NA |
| **5** | Y | Y | Y | Y | Y | Y | Y | Y | Y | Y |
| **6** | Y | Y | Y | Y | Y | Y | Y | Y | Y | Y |
| **7** | N | Y | Y | Y | N | Y | Y | N | Y | N |
| **8** | Y | Y | Y | N | Y | Y | Y | Y | Y | Y |
| **9** | Y | N | Y | Y | Y | Y | Y | Y | Y | Y |
| **10** | Y | Y | Y | Y | Y | U | Y | Y | Y | Y |
| **11** | Y | U | U | U | Y | NA | U | U | U | U |
| **12** | Y | Y | Y | Y | Y | Y | Y | Y | Y | Y |
| **13** | N | U | N | Y | Y | N | N | Y | NA | Y |
| **14** | NA | NA | NA | NA | NA | NA | NA | NA | NA | NA |
| **Overall Quality Assessment** | Medium | High | High | High | High | High | Medium | Medium | High | High |

Y=Yes; N=No; U=Unclear; NA=Not Applicable

| **QUADAS Question no.** | **Jones[84]** | **Kirkman[24]** | **Klatsky[45]** | **Kokotalio[85]** | **Koster[60]** | **Krarup[86]** | **Kumamaru[87]** | **Lakshminarayan, 2009[46]** | **Lakshminarayan, 2014[88]** | **Lambert[89]** |
| --- | --- | --- | --- | --- | --- | --- | --- | --- | --- | --- |
| **1** | Y | Y | Y | Y | Y | Y | N | Y | N | N |
| **2** | Y | Y | Y | Y | Y | Y | Y | Y | Y | Y |
| **3** | Y | N | Y | U | Y | Y | Y | Y | Y | U |
| **4** | NA | NA | NA | NA | NA | NA | NA | NA | NA | NA |
| **5** | Y | Y | Y | Y | Y | Y | Y | Y | Y | Y |
| **6** | Y | Y | Y | Y | Y | Y | Y | Y | Y | Y |
| **7** | Y | N | N | N | N | N | N | N | Y | Y |
| **8** | Y | Y | Y | Y | Y | Y | Y | Y | Y | N |
| **9** | Y | Y | Y | N | U | Y | Y | Y | Y | Y |
| **10** | Y | Y | Y | Y | U | Y | Y | Y | Y | Y |
| **11** | U | U | U | N | Y | U | U | U | Y | Y |
| **12** | Y | Y | Y | Y | Y | Y | Y | Y | Y | Y |
| **13** | Y | Y | Y | N | Y | Y | Y | N | Y | Y |
| **14** | NA | NA | NA | NA | NA | NA | NA | NA | NA | NA |
| **Overall Quality Assessment** | High | Medium | High | Medium | High | High | High | High | High | Medium |

Y=Yes; N=No; U=Unclear; NA=Not Applicable

| **QUADAS Question no.** | **Lee[90]** | **Leibson[91]** | **Lentine[92]** | **Leone[93]** | **Leppala[51]** | **Levy[94]** | **Lindblad[53]** | **Liu[26]** | **Mayo[95]** | **Olson[96]** |
| --- | --- | --- | --- | --- | --- | --- | --- | --- | --- | --- |
| **1** | Y | Y | N | Y | N | N | Y | Y | Y | Y |
| **2** | Y | Y | Y | Y | Y | Y | Y | Y | Y | Y |
| **3** | Y | Y | U | Y | Y | N | Y | Y | Y | Y |
| **4** | NA | NA | NA | NA | NA | NA | NA | NA | NA | NA |
| **5** | Y | Y | Y | Y | Y | Y | Y | Y | Y | Y |
| **6** | Y | Y | Y | Y | Y | Y | Y | Y | Y | Y |
| **7** | N | N | Y | Y | N | N | N | N | N | N |
| **8** | Y | Y | Y | Y | Y | Y | Y | Y | Y | Y |
| **9** | Y | Y | Y | Y | Y | Y | Y | Y | Y | N |
| **10** | Y | Y | Y | Y | Y | Y | Y | Y | Y | Y |
| **11** | U | U | NA | U |  | NA | U | U | Y | Y |
| **12** | Y | Y | N | Y | Y | Y | Y | Y | Y | Y |
| **13** | Y | U | NA | Y | Y | NA | Y | N | N | N |
| **14** | NA | NA | NA | NA | NA | NA | NA | NA | NA | NA |
| **Overall Quality Assessment** | High | High | Medium | High | High | Medium | High | High | High | High |

Y=Yes; N=No; U=Unclear; NA=Not Applicable

| **QUADAS Question no.** | **Newton[97]** | **Palmieri[47]** | **Phillips[55]** | **Piriyawat[98]** | **Ramalle-Gomara[99]** | **Rampatige[61]** | **Rao[56]** | **Reggio[64]** | **Reker[100]** | **Rinaldi[23]** |
| --- | --- | --- | --- | --- | --- | --- | --- | --- | --- | --- |
| **1** | N | Y | Y | Y | Y | N | N | Y | N | Y |
| **2** | Y | Y | Y | Y | Y | Y | Y | Y | Y | Y |
| **3** | N | Y | Y | Y | Y | Y | Y | Y | U | Y |
| **4** | NA | NA | NA | NA | NA | NA | NA | NA | NA | NA |
| **5** | Y | N | Y | Y | Y | Y | Y | Y | Y | Y |
| **6** | Y | Y | Y | Y | Y | Y | Y | Y | Y | Y |
| **7** | Y | N | N | U | NA | Y | Y | N | Y | N |
| **8** | Y | Y | Y | Y | Y | Y | Y | Y | Y | Y |
| **9** | Y | Y | N | Y | Y | Y | Y | Y | U | Y |
| **10** | Y | Y | Y | U | Y | Y | Y | Y | Y | Y |
| **11** | Y | U | U | Y | NA | Y | U | NA | U |  |
| **12** | Y | Y | Y | Y | Y | Y | Y | U | Y | Y |
| **13** | Y | N | Y | N | U | Y | Y | Y | Y | Y |
| **14** | NA | NA | NA | NA | NA | NA | NA | NA | NA | NA |
| **Overall Quality Assessment** | High | Medium | High | High | High | High | High | Medium | High | High |

Y=Yes; N=No; U=Unclear; NA=Not Applicable

| **QUADAS Question no.** | **Rosamond[49]** | **Roumie[101]** | **Shahar[48]** | **Singh[102]** | **Sinha[103]** | **So[104]** | **Soo[105]** | **Spolaore[106]** | **Stegmayr[58]** | **Szczesniewska[59]** |
| --- | --- | --- | --- | --- | --- | --- | --- | --- | --- | --- |
| **1** | Y | Y | Y | N | Y | N | N | Y | Y | Y |
| **2** | Y | Y | Y | Y | Y | Y | Y | Y | Y | Y |
| **3** | Y | Y | Y | Y | Y | U | Y | Y | Y | Y |
| **4** | NA | NA | NA | NA | NA | NA | NA | NA | NA | NA |
| **5** | Y | Y | Y | Y | Y | Y | Y | Y | Y | Y |
| **6** | Y | Y | Y | Y | Y | Y | Y | Y | Y | Y |
| **7** | N | N | N | Y | N | Y | Y | N | N | N |
| **8** | Y | Y | Y | N | Y | Y | Y | Y | N | Y |
| **9** | Y | Y | Y | Y | Y | N | Y | Y | Y | N |
| **10** | Y | Y | Y | Y | Y | Y | Y | Y | U | Y |
| **11** | Y | U | U | Y | U | U | Y | U | Y | N |
| **12** | Y | Y | Y | Y | Y | Y | Y | Y | Y | U |
| **13** | N | Y | N | NA | Y | NA | Y | N | Y | Y |
| **14** | NA | NA | NA | NA | NA | NA | NA | NA | NA | NA |
| **Overall Quality Assessment** | High | High | High | High | High | Medium | Medium | High | High | Medium |

Y=Yes; N=No; U=Unclear; NA=Not Applicable

| **QUADAS Question no.** | **Thigpen[107]** | **Tirschwell[108]** | **Tolonen[52]** | **Tu[109]** | **Wahl[110]** | **Wildenschild[111]** | **Wu[112]** |
| --- | --- | --- | --- | --- | --- | --- | --- |
| **1** | N | Y | Y | Y | Y | N | Y |
| **2** | Y | Y | Y | Y | Y | Y | Y |
| **3** | Y | Y | Y | U | Y | Y | N |
| **4** | NA | NA | NA | NA | NA | NA | NA |
| **5** | Y | Y | Y | Y | Y | Y | Y |
| **6** | Y | Y | Y | Y | Y | Y | Y |
| **7** | N | N | N | Y | N | N | Y |
| **8** | Y | Y | Y | Y | Y | N | Y |
| **9** | Y | Y | N | N | Y | Y | Y |
| **10** | Y | Y | Y | Y | Y | Y | Y |
| **11** | Y | Y | U | Y | U | U | Y |
| **12** | Y | Y | Y | U | Y | Y | NA |
| **13** | N | N | N | Y | U | Y | NA |
| **14** | NA | NA | NA | NA | NA | NA | NA |
| **Overall Quality Assessment** | Medium | High | High | High | High | Medium | Medium |

Y=Yes; N=No; U=Unclear; NA=Not Applicable
