# Supplementary material for: Validity of Diagnostic Codes for Acute Stroke in Administrative Databases: A Systematic Review
Source: PLoS One. 2015 Aug 20;10(8):e0135834. doi: 10.1371/journal.pone.0135834 (PMC4546158; doi:10.1371/journal.pone.0135834)
Supplement: S2 Table — (DOC) [file pone.0135834.s008.doc]

**S2 Table. Results of Studies Validating Diagnoses of Stroke** in Administrative Data.

| **First Author, Year** | **Diagnostic Codes** | **Parameter** | **Sensitivity (95% CI)** | **Specificity (95% CI)** | **PPV (95% CI)** | **NPV**  **(95% CI)** | **Kappa**  **(95% CI)** | **Quality** |
| --- | --- | --- | --- | --- | --- | --- | --- | --- |
| **ANY FORM OF CEREBROVASCULAR DISEASE** | | | | | | | | |
| **Borzecki**[1], 2004 | ICD-9-CM 430.x-438.x , ≥ 1 diagnosis, looking over past year | cerebrovascular disease | 64 | 95 | 48.28 (38.97-57.70) |  | 0.51 | Medium |
|  | ICD-9-CM 430.x-438.x , ≥ 1 diagnosis, looking over past two years |  |  |  |  |  | 0.49 |  |
|  | ICD-9-CM 430.x-438.x , ≥ 2 diagnosis, looking over past year |  |  |  |  |  | 0.53 |  |
|  | ICD-9-CM 430.x-438.x , ≥ 2 diagnosis, looking over past two years |  |  |  |  |  | 0.53 |  |
| **Chen**[2], 2009 | ICD-10 G45.x, G46.x, H34.0, I60.x-I69.x | cerebrovascular disease | 46.32 (40.83-51.90) | 99.16 (98.79-99.42) | 82.97 (74.54-87.97) | 95.43 (94.70-96.06) |  | Medium |
|  |  | cerebrovascular disease: regular kappa |  |  |  |  | 0.57 |  |
|  |  | cerebrovascular disease: PABAK |  |  |  |  | 0.90 |  |
| **de Faire**[3], 1976 | ICD-1965 390-458, 410-414, 420-429, 430-438 | cerebrovascular disease as a COD | 96.00 (89.49-98.71) | 99.72 (99.10-99.93) | 96.97 (90.76-99.21) | 99.62 (98.96-99.88) |  | High |
| **Hasan**[4], 1995 | ICD-9 430-438, primary diagnosis | cerebrovascular disease |  |  | 73.49 (65.99-79.89) |  |  | Medium |
| **Henderson**[5], 2006 | ICD-10 hospital discharge codes: G46, I60, I61, I62, I63, I64, I65, I66, I69, G450, G451, G452, G454, G458, G459, I670, I671, I672, I674, I675, I676, I677, I678, I679, I681, I682, I688 | cerebrovascular disease as comorbidity: 1998-1999 | 86.22 (80.41-90.57) | 99.81 (99.66-99.89) | 92.86 (87.83-95.98) | 99.60 (99.42-99.73) | 0.89 | High |
|  |  | cerebrovascular disease as comorbidity: 2000-2001 | 88.89 (83.46-92.75) | 99.83 (99.69-99.90) | 93.12 (88.27-96.13) | 99.70 (99.54-99.81) | 0.91 |  |
| **Hennessy**[6], 2010 | ICD-10 G45.x, G46.x, H34.0, I60.x-I69.x; low volume coders | cerebrovascular disease |  |  |  |  | 0.63 | High |
|  | ICD-10 G45.x, G46.x, H34.0, I60.x-I69.x; high-volume coders |  |  |  |  |  | 0.51 |  |
|  | ICD-10 G45.x, G46.x, H34.0, I60.x-I69.x; part-time coders |  |  |  |  |  | 0.52 |  |
|  | ICD-10 G45.x, G46.x, H34.0, I60.x-I69.x; full-time coders |  |  |  |  |  | 0.64 |  |
|  | ICD-10 G45.x, G46.x, H34.0, I60.x-I69.x; non-tertiary hospitals |  |  |  |  |  | 0.36 |  |
|  | ICD-10 G45.x, G46.x, H34.0, I60.x-I69.x; tertiary hospitals |  |  |  |  |  | 0.63 |  |
| **Humphries**[7], 2000 | ICD-9 430-438 | previous cerebrovascular disease: preadmission co-morbid diagnosis (previous history of stroke, transient ischemic attack or carotoid endarterectomy) | 42.86 (26.76-60.48) | 99.23 (98.25-99.69) | 71.43 (47.69-87.81) | 97.49 (96.08-98.42) | 0.52 | Medium |
| **Lambert**[8], 2012 | ICD-9 430-438, primary or secondary position | cerebrovascular disease, overall | 85.8 (77.7-91.9) | 97.8 (97.0-98.4) | 68.9 (62.4-75.5) | 99.2 (98.7-99.6) | 0.750 (0.687-0.813) | Medium |
|  |  | primary hospitals | 92.1 (77.5-97.9) | 96.3 (94.1-97.8) | 67.3 (52.8-79.3) | 99.3 (97.9-99.8) | 0.756 |  |
|  |  | secondary hospitals | 81.5 (61.3-93.0) | 98.3 (96.9-99.1) | 66.7 (48.1-81.4) | 99.2 (98.1-99.7) | 0.721 |  |
|  |  | tertiary hospitals | 82.9 (67.4-92.3) | 98.3 (97.0-99.0) | 72.3 (57.1-83.9) | 99.1 (98.0-99.6) | 0.760 |  |
| **Lee**[9], 2005 | ICD-9-CM 430-438 | cerebrovascular disease as a comorbidity, index admission | 24.23 (19.96-29.07) | 99.22 (98.52-99.60) | 89.69 (81.44-94.67) | 82.38 (80.37-84.23) |  | High |
|  |  | cerebrovascular disease as a comorbidity, enhanced definition (up to one year prior to index admission) | 31.75 (27.02-36.89) | 98.28 (97.37-98.90) | 83.82 (76.31-89.37) | 83.72 (81.74-85.53) |  |  |
| **Leone**[10], 2004 | ICD-9 430-438 as primary or secondary hospital discharge diagnosis | for any cerebrovascular disease overall | 89 (87-91) |  | 88 (86-90) |  |  | High |
|  |  | men | 91 |  | 86 |  |  |  |
|  |  | women | 87 |  | 90 |  |  |  |
|  | ICD-9 430-438 as primary hospital discharge diagnosis | for any cerebrovascular disease overall | 77 (75-80) |  | 93 (91-95) |  |  |  |
| **Levy**[11], 1999 | ICD-9 362.3, 430-436, 437.1, 438, 784.3, 997.0 as hospital discharge diagnosis | cerebrovascular disease as MI comorbidity | 57 (51-64) |  |  | 96 (93-98) |  | Medium |
| **Rampatige**[12], 2013 | ICD-10 I60-69 | cerebrovascular disease as underlying COD | 31.5 (21.8-39.9) |  | 60.7 (42.1-77.0) |  |  | High |
| **Rao**[13], 2007 | ICD-10 I60-I69 | cerebrovascular disease as underlying COD | 81.6 (78.0-84.8) |  | 88.4 (85.2-91.1) |  |  | High |
| **Singh**[14], 2012 | ICD-9 430-438 | cerebrovascular disease | 55 (39-70) | 90 (85-94) | 52 | 91 |  | High |
| **So**[15], 2006 | ICD-9 430.x-438.x; | cerebrovascular disease as an MI comorbidity | 100 (51.68-100) | 93.58 (88.79-96.49) | 33.33 (14.36-58.85) | 100 (97.32-100) |  | Medium |
|  | ICD-10 G45.x, G46.x, H34.0, I60.x-I69.x |  | 100 (51.68-100) | 95.72 (91.45-98.00) | 42.86 (18.81-70.35) | 100 (97.38-100) |  |  |
| **Soo**[16], 2014 | ICD-10 I60.x-I69.x, G45x, G46x, H34.0, I63, I64, I69.3, I69.4 | cerebrovascular disease, overall | 87.1 (82.4-90.7) | 97.8 (97.2-98.3) | 76.6 (71.3-81.2) | 98.9 (98.5-99.2) | 0.8 | Medium |
|  |  | males | 86.6 (79.3-91.6) | 97.8 (96.8-98.5) | 80.6 (73.0-86.5) | 98.6 (97.7-99.1) | 0.82 |  |
|  |  | females | 87.7 (79.9-92.9) | 97.8 (96.9-98.4) | 72.5 (64.1-80.0) | 99.2 (98.6-99.5) | 0.78 |  |
| **ACUTE STROKE** | | | | | | | | |
| **Broderick**[17], 1998 | ICD-9-CM 430-438, primary or secondary position | acute stroke |  |  | 45.98 (42.33-49.66) |  |  | High |
| **Brown**[18], 2006 | ICD-10 I60-I69 as underlying COD | acute stroke: neurologist 1 | 66.67 (51.97-78.85) |  | 45.95 (34.44-57.87) |  |  | High |
|  |  | acute stroke: neurologist 2 | 62.71 (49.11-74.66) |  | 50.00 (38.25-61.75) |  |  |  |
| **Ellekajer**[19], 1999 | ICD-9 430-438.9 (all positions) | acute stroke vs. all hospitalized register cases | 94.86 (92.04-96.75) |  | 48.62 (45.01-52.24) |  |  | High |
| **Iso**[20], 1990 | ICD-8 or ICD-9 430-438 | stroke as underlying COD on death certificate | 65.93 (60.39-71.08) |  | 97.66 (94.33-99.14) |  |  |  |
| **Ives**[21], 1995 | ICD-9-CM 430-438 | stroke as COD | 42.11 (20.25-66.50) |  | 61.54 (31.58-86.14) |  |  | High |
| **Johnsen**[22], 2002 | ICD-10 I60-69.8 (main and secondary diagnoses) | stroke overall (SAH, ICH, ischaemic, unspecified) | 92 (88-95) | 68 (61-73) | 79.30 (74.9-83.3) | 86 (80-91) |  | High |
| **Jone**s[23], 2014 | ICD-9-430-438, any position | any stroke, definite or probable |  |  | 34.59 (33.13-36.09) |  |  | High |
|  | ICD-9-CM, primary position |  |  |  | 48.79 (46.82-50.76) |  |  |  |
| **Klatsky**[24], 2005 | ICD-9 430-438 as primary discharge diagnosis | acute stroke |  |  | 77 (76-78) |  |  | High |
| **Krarup**[25], 2007 | ICD-10 hospital discharge code I60-I69, G45 | reviewer 1: stroke (SAH, ICH, ischaemic, unspecified) overall |  |  | 80.50 (73.6-86.3) |  |  | High |
|  |  | reviewer 2: stroke (SAH, ICH, ischaemic, unspecified) overall |  |  | 86.0 (79.7-90.9) |  |  |  |
| **Leibson**[26], 1999 | ICD-9 430-438.9, first diagnosis | incident |  |  | 46.95 (41.84-52.12) |  |  | High |
|  | ICD-9 430-438.9, first 3 diagnoses |  |  |  | 42.86 (38.32-47.52) |  |  |  |
|  | ICD-9 430-438.9, first diagnosis | incident or prevalent |  |  | 59.68 (54.52-64.64) |  |  |  |
|  | ICD-9 430-438.9, first 3 diagnoses |  |  |  | 53.90 (49.23-58.50) |  |  |  |
|  | ICD-9 430-438.9, first diagnosis |  | 76.36 (71.18-80.87) |  |  |  |  |  |
|  | ICD-9 430-438.9, 1st or 2nd diagnosis |  | 86.58 (82.18-90.06) |  |  |  |  |  |
|  | ICD-9 430-438.9, 1st-3rd diagnosis |  | 90.42 (86.47-93.34) |  |  |  |  |  |
|  | ICD-9 430-438.9, 1st-4th diagnosis |  | 91.37 (87.56-94.14) |  |  |  |  |  |
|  | ICD-9 430-438.9, 1st-5th diagnosis |  | 92.65 (89.03-95.18) |  |  |  |  |  |
| **Leone**[10], 2004 | ICD-9 430-438 | any stroke: SAH, ICH, ischaemic, or stroke unknown (primary and secondary diagnosis) | 87 (84-90) |  | 59.88 (57-63) |  |  | High |
|  |  | any stroke: SAH, ICH, ischaemic, or stroke unknown (primary diagnosis only) | 79 (76-82) |  | 66.02 (63-69) |  |  |  |
| **Liu**[27], 1999 | ICD-9 430-438, primary diagnosis | all hospitals combined, definite stroke | 96 (93-98) |  | 35 (31-38) |  |  | High |
|  |  | all hospitals combined, definite or highly-probable stroke | 96 (94-98) |  | 65 (62-69) |  |  |  |
|  | ICD-9 430-438, primary, secondary, or tertiary diagnosis | all hospitals combined, definite stroke | 96 (93-97) |  | 27 (25-30) |  |  |  |
|  |  | all hospitals combined, definite or highly-probable stroke | 92 (89-94) |  | 54 (51-57) |  |  |  |
| **Palmieri**[28], 2007 | ICD-9 430-438 | nonfatal total |  |  | 49.05 (47.52-50.58) |  |  | Medium |
|  |  | nonfatal women |  |  | 48.47 (46.05-50.90) |  |  |  |
|  |  | nonfatal men |  |  | 49.44 (47.46-51.42) |  |  |  |
| **Phillips**[29], 1993 | ICD-9 430-438 as primary or secondary discharge diagnosis | acute stroke |  |  | 34.88 (29.56-40.60) |  |  | High |
|  | ICD-9 430-438 as COD on death certificate | acute stroke | 82.35 (55.80-95.33) |  |  |  |  |  |
| **Ramalle-Gomara**[30], 2013 | ICD-9-CM 430-438, primary diagnosis | stroke | 96.1 (92.6-99.5) | 87.5 (83.2-91.8) | 82.5 (76.6-88.4) | 97.3 (95.0-99.7) |  | High |
|  | ICD-9-CM 430-438, primary or secondary diagnosis |  | 96.1 (92.6-99.5) | 38.3 (32.1-44.6) | 48.8 (43.0-54.7) | 94.1 (89.0-99.2) |  |  |
| **Reggio**[31], 1995 | ICD-9 430-438 | stroke as initial COD |  |  | 54.31 (44.81-63.59) |  |  | Medium |
|  |  | stroke as initial or intermediate COD |  |  | 52.43 (44.98-59.81) |  |  |  |
|  |  | stroke as initial, intermediate, or terminal COD | 60.00 (38.67-78.87) |  | 52.33 (45.04-59.55) |  |  |  |
| **Rosamond**[32], 1999 | ICD-9-CM 430-438 | definite or probable stroke |  |  | 27.76 (25.25-30.42) |  |  | High |
| **Sinha**[33], 2008 | ICD-10 I60-I69 as hospital admission, primary or contributing COD | definite stroke |  |  | 76 (71-81) |  |  | High |
|  |  | definite or probable stroke |  |  | 84 (79-89) |  |  |  |
|  |  | definite or probable or possible stroke |  |  | 89 (84-92) |  |  |  |
|  |  | true incident stroke (no previous history of  stroke) |  |  | 90 (85-93) |  |  |  |
| **Tolonen**[34], 2007 | first-ever main or additional hospital discharge diagnosis ICD-9 430-438, ICD-10 I60-69; deaths with stroke as direct or underlying cause ICD-9 430-434, 436-438 or ICD-10 I60-I69 | all cases vs. first-ever stroke | 85 (84-86) |  | 86 (85-87) |  |  | High |
|  |  | fatal cases | 86 (84-88) |  | 92 (90-94) |  |  |  |
|  |  | nonfatal cases | 85 (84-86) |  | 85 (84-86) |  |  |  |
|  |  | men | 84 (82-86) |  | 87 (85-89) |  |  |  |
|  |  | women | 86 (85-87) |  | 86 (85-87) |  |  |  |
|  | all ICD-9 stroke codes | first-ever stroke | 88 (86-90) |  | 87 (85-89) |  |  |  |
|  | all ICD-10 stroke codes | first-ever stroke | 83 (81-85) |  | 85 (83-87) |  |  |  |
| **Wildenschild**[35] | ICD-10 I60-I69, G00-G09, G40-G47, G81-G83, R25-R29, S062-S069, Z501. | acute stroke | 58.11 (46.08-69.29) |  | 93.48 (81.08-98.30) |  |  | Medium |
| **Wu**[36], 2014 | ICD-9-CM 430-438 inpatient or outpatient diagnosis, in any position | stroke | 66.84 (59.54-73.44) |  | 32.05 (27.49-36.97) |  |  | Medium |
| **SUBARACHNOID HAEMORRHAGE** | | | | | | | | |
| **Agrawal**[37] | ICD-9 430, inpatient diagnosis | ischaemic or haemorrhagic stroke |  |  | 82 (66-92) |  |  | Medium |
|  | ICD-9 430, outpatient diagnosis only |  |  |  | 100 (16-100) |  |  |  |
|  | ICD-9 430, inpatient or outpatient diagnosis |  |  |  | 83 (68-93) |  |  |  |
| **Broderick**[17], 1998 | ICD-9-CM 430, primary or secondary position | acute stroke |  |  | 64.29 (35.63-86.02) |  |  | High |
| **Ellekjaer**[19], 1999 | ICD-9 430 | any stroke, first-ever |  |  | 69.23 (38.88-89.64) |  |  | High |
|  |  | any stroke, first or recurrent |  |  | 69.23 (38.88-89.64) |  |  | High |
| **Gaist**[38], 2000 | ICD-8 430 or ICD-10 I60 | SAH, discharged from neurosurgery ward |  |  | 93 (85-98) |  |  |  |
|  |  | SAH, discharged from neurology ward |  |  | 75 (60-87) |  |  |  |
|  |  | SAH, discharged from non-specialty ward |  |  | 47 (36-59) |  |  |  |
|  |  | SAH, > 1 case of SAH in the family |  |  | 92 (78-98) |  |  |  |
| **Heckbert**[39], 2004 | ICD-9 430 | any stroke |  |  | 74 (58-86) |  |  | High |
| **Ives**[21], 1995 | ICD-9-CM 430 | incident stroke |  |  | 100 (15.81-100) |  |  | High |
| **Johnsen**[22], 2002 | ICD-10 I60 | SAH |  |  | 48.30 (29.4-67.5) |  |  | High |
| **Jones**[23], 2014 | ICD-9-CM 430, any position | any stroke, definite or probable |  |  | 79 (66-88) |  |  | High |
|  | ICD-9-CM 430, primary position |  |  |  | 86.27 (74-94) |  |  |  |
|  | ICD-9-CM 430, any position | SAH |  |  | 73 (60-84) |  |  |  |
| **Kirkman**[40], 2009 | ICD10 I60.x | SAH |  |  | 96.10 (94.8-97.0) |  |  | Medium |
| **Kokotailo**[41], 2005 | ICD-9 430.x | SAH |  |  | 98 (90-99) |  |  | Medium |
|  | ICD-10 I60.x | SAH |  |  | 91 (77-98) |  |  |  |
| **Krarup**[25], 2007 | ICD-10 I60 | SAH, reviewers 1&2 |  |  | 67 (13-98) |  |  | High |
| **Leibson**[26], 1999 | ICD-9 430 (up to 5 diagnostic positions) | SAH, incident |  |  | 100 (67.86-100) |  |  | High |
|  |  | SAH, incident or prevalent |  |  | 100 (67.86-100) |  |  |  |
| **Leone**[10], 2004 | ICD-9 430, primary or secondary position | SAH | 35 (23-50) |  | 42.86 (28.08-58.93) |  |  | High |
|  | ICD-9 430, primary position |  | 33 (21-48) |  | 45.95 (29.85-62.87) |  |  |  |
|  | ICD-9 430, primary or secondary position | any stroke |  |  | 76.19 (60.20-87.41) |  |  |  |
|  | ICD-9 430, primary position |  |  |  | 81.08 (64.29-91.44) |  |  |  |
| **Leppala**[42], 1999 | ICD-8 or ICD-9 430 | SAH, hospital discharge diagnosis |  |  | 78.6 (59-91) |  |  | High |
|  |  | SAH, COD |  |  | 95.2 (83-99) |  |  |  |
|  |  | SAH, hospital discharge diagnosis (diagnosis present in hospital and vital statistics data) |  |  | 100 |  |  |  |
|  |  | SAH, COD (diagnosis present in hospital and vital statistics data) |  |  | 100 |  |  |  |
| **Lindblad**[43], 1993 | ICD-8&9 430 | SAH |  |  | 78.26 (55.79-91.71) |  |  | High |
|  |  | acute stroke |  |  | 91.30 (70.49-98.48) |  |  |  |
| **Liu**[27], 1999 | ICD-9 430, primary | all hospitals combined, definite stroke |  |  | 87 (58-98) |  |  | High |
|  |  | all hospitals combined, definite or highly-probable stroke |  |  | 87 (58-98) |  |  |  |
|  | ICD-9 430, primary/secondary/tertiary | all hospitals combined, definite stroke |  |  | 82 (56-95) |  |  |  |
|  |  | all hospitals combined, definite or highly-probable stroke |  |  | 82 (56-95) |  |  |  |
| **Mayo**[44], 1993 | ICD-9 430 | acute stroke, neurologist #1 |  |  | 100 (5.46-100) |  |  |  |
|  |  | acute stroke, neurologist #2 |  |  | 100 (5.46-100) |  |  | High |
| **Olson**[45], 2014 | ICD-9 430.xx, inpatient diagnosis | any stroke |  |  | 60 (47-71) |  |  |  |
|  | ICD-9 430.xx, outpatient diagnosis | any stroke |  |  | 46 (38-54) |  |  | High |
|  | ICD-9 430.xx, inpatient and outpatient diagnosis | any stroke |  |  | 56 (40-72) |  |  |  |
|  | ICD-9 430.xx, inpatient diagnosis | SAH or ICH |  |  | 78 (62-90) |  |  |  |
|  | ICD-9 430.xx, outpatient diagnosis | SAH or ICH |  |  | 77 (65-86) |  |  |  |
|  | ICD-9 430.xx, inpatient and outpatient diagnosis | SAH or ICH |  |  | 83 (61-95) |  |  |  |
| **Palmieri**[28], 2007 | ICD-9 430 | non-fatal, primary diagnosis, men vs. nonfatal CV events (MONICA stroke categories definite or associated w/ definite MI) |  |  | 72 (60-82) |  |  | Medium |
|  |  | non-fatal, primary diagnosis, women, vs. nonfatal CV events (MONICA stroke categories definite or associated w/ definite MI) |  |  | 74 (64-82) |  |  |  |
|  |  | non-fatal total |  |  | 73 (66-80) |  |  |  |
|  |  | fatal men vs. fatal CV event (MONICA stroke categories definite, associate w/definite or possible MI, or unclassifiable) |  |  | 80 (56-93) |  |  |  |
|  |  | fatal women vs. fatal CV event (MONICA stroke categories definite, associate w/definite or possible MI, or unclassifiable) |  |  | 100 (80-100) |  |  |  |
|  |  | fatal total |  |  | 90 (75.40-96.75) |  |  |  |
| **Phillips**[29], 1993 | ICD-9 430 as primary or secondary discharge diagnosis | acute stroke |  |  | 33.33 (1.76-87.47) |  |  | High |
| **Rao**[13], 2007 | ICD-10 I60 | SAH as underlying COD | 60 |  |  |  |  | High |
| **Reker**[46], 2001 | ICD-9 430.x discharge diagnosis | new stroke |  |  | 33 (11-65) |  |  | High |
| **Rosamond**[32], 1999 | ICD-9-CM 430 | definite or probable stroke |  |  | 86.36 (64.04-96.41) |  |  | High |
|  |  | definite or probable SAH |  |  | 86.36 (64.04-96.41) |  |  |  |
| **Roumie**[47], 2008 | ICD-9 430 | SAH |  |  | 100 (19.79-100) |  |  | High |
| **Spolare**[48], 2005 | ICD-9 430 | hospital discharge primary position vs. stroke (MONICA criteria definite or definite stroke associated w/definite MI) |  |  | 76 (73-79) |  |  | High |
| **Tirschwell**[49], 2002 | ICD-9-CM 430, up to nine discharge positions | SAH |  |  | 86 (75-94) |  |  | High |
|  | ICD-9-CM 430, first two discharge positions | SAH |  |  | 89 (77-96) |  |  |  |
|  | ICD-9-CM 430, primary discharge position only | SAH |  |  | 94 (83-99) |  |  |  |
| **Tolonen**[34], 2007 | ICD-9 430 or ICD-10 I60 | all cases vs. SAH | 93 (90-96) |  | 87 (83-91) |  |  | High |
|  |  | fatal cases | 95 (91-99) |  | 97 (94-100) |  |  |  |
|  |  | non-fatal cases | 92 (88-96) |  | 81 (75-87) |  |  |  |
|  |  | men | 93 (88-98) |  | 87 (81-93) |  |  |  |
|  |  | women | 93 (89-97) |  | 87 (82-92) |  |  |  |
|  | ICD-9 430 | SAH | 92 (88-96) |  | 88 (83-93) |  |  |  |
|  | ICD-10 I60 | SAH | 95 (91-99) |  | 85 (79-91) |  |  |  |
| **HAEMORRHAGIC STROKE** | | | | | | | | |
| **Aboa-Eboule**[50], 2013 | ICD-10 I61 | ICH | 87.85 (79.77-93.12) |  | 64.8 |  |  | High |
| **Agrawal**[37], 2009 | ICD-9 431, inpatient diagnosis | ischaemic or haemorrhagic stroke |  |  | 79 (65-89) |  |  | Medium |
|  | ICD-9 431, outpatient diagnosis only |  |  |  | 49 (37-61) |  |  |  |
|  | ICD-9 431, inpatient or outpatient diagnosis |  |  |  | 62 (53-70) |  |  |  |
| **Arnason**[51], 2006 | ICD-9-CM 430-432 | intracranial bleed |  |  | 94 (86-97) |  |  | High |
| **Broderick**[17], 1998 | ICD-9-CM 431, primary or secondary position | acute stroke |  |  | 83.33 (65.55-93.70) |  |  | High |
|  | ICD-9-CM 432, primary or secondary position |  |  |  | 0.00 (0-48.32) |  |  |  |
|  | ICD-9-CM 431 or 432, primary or secondary position |  |  |  | 69.44 (51.73-83.08) |  |  |  |
| **Derby**[52], 2000 | ICD-9 431-432 | any stroke |  |  | 70.30 (65-75) |  |  | High |
| **Ellekajer**[19], 1999 | ICD-9 431 | any stroke, first-ever |  |  | 76.79 (63.27-86.60) |  |  | High |
|  |  | any stroke, first or recurrent |  |  | 87.50 (75.31-94.41) |  |  |  |
|  | ICD-9 432 | any stroke, first-ever |  |  | 1.50 (.656-53.32) |  |  |  |
|  |  | any stroke, first or recurrent |  |  | 25.00 (4.45-64.42) |  |  |  |
|  | ICD-9 431&432 | any stroke, first-ever |  |  | 68.75 (55.80-79.43) |  |  |  |
|  |  | any stroke, first or recurrent |  |  | 79.69 (67.42-88.33) |  |  |  |
| **Heckbert**[39], 2004 | ICD-9 431 | any stroke |  |  | 93 (84-97) |  |  | High |
|  | ICD-9 432.0-432.1 |  |  |  | 24 (9-48) |  |  |  |
|  | ICD-9 432.9 |  |  |  | 60 (17-93) |  |  |  |
| **Iso**[20], 1990 | ICD-8 431 or ICD-9 431-432 | stroke as underlying COD on death certificate |  |  | 98.82 (92.71-99.94) |  |  | Medium |
|  | ICD-8 431 or ICD-9 431-432 | intracranial haemorrhage as COD on death certificate | 71.08 (59.94-80.25) |  | 69.41 (58.34-78.70) |  |  |  |
| **Ives**[21], 1995 | ICD-9-CM 431 | incident stroke |  |  | 100 (39.76-100) |  |  | High |
|  | ICD-9-CM 432.0-1 |  |  |  | 0 (0-97.50) |  |  |  |
|  | ICD-9-CM 432.9 |  |  |  | 100 (2.50-100) |  |  |  |
| **Johnsen**[22], 2002 | ICD-10 I61 | ICH |  |  | 65.70 (47.80-80.90) |  |  | High |
| **Jones**[23], 2014 | ICD-9-CM 431, any position | any stroke, definite or probable |  |  | 69 (61-76) |  |  | High |
|  | ICD-9-CM 431, primary position |  |  |  | 79.23 (71-86) |  |  |  |
|  | ICD-9-CM 431, any position | ICH |  |  | 57 (49-65) |  |  |  |
|  |  | ICH, males |  |  | 49 (38-60) |  |  |  |
|  |  | ICH, females |  |  | 65 (54-76) |  |  |  |
|  |  | ICH, incident |  |  | 62 (52-71) |  |  |  |
|  | ICD-9-CM 431, primary position | ICH |  |  | 71 (62-78) |  |  |  |
|  | ICD-9-CM 432, any position | any stroke, definite or probable |  |  | 8 (3-15) |  |  |  |
|  | ICD-9-CM 432, primary position |  |  |  | 11.86 (5-23) |  |  |  |
|  | ICD-9-CM 431/432, any position |  |  |  | 46.59 (40.29-52.99) |  |  |  |
|  | ICD-9-CM 431/432, primary position |  |  |  | 58.20 (50.81-65.25) |  |  |  |
|  | ICD-9-CM 431/432, any position | ICH |  |  | 40 (34-46) |  |  |  |
| **Kirkman**[22], 2009 | ICD-10 I61.x | ICH |  |  | 95.9 (94.5-97.0) |  |  | Medium |
| **Kokotailo**[41], 2005 | ICD-9 431.x | ICH |  |  | 97 (91-99.7) |  |  | Medium |
|  | ICD-10 I61.x | ICH |  |  | 98 (92-99.0) |  |  |  |
| **Krarup**[25], 2007 | ICD-10 I61 | ICH, reviewers 1&2 |  |  | 74 (52-90) |  |  | High |
| **Kumamaru**[53], 2014 | ICD-9-CM 430 or 431, primary discharge diagnosis | ICH, first-ever or recurrent | 67.4 (54.8-79.9) | 100.0 (99.9-100) | 88.6 (78.9-98.3) | 98.9 (98.7-99.0) |  | High |
|  |  | ICH, first-ever | 59.9 (44.9-74.9) | 100.0 (99.9-100) | 84.8 (71.8-97.9) | 99.9 (99.8-99.9) |  |  |
|  |  | ICH, first-ever or recurrent, women | 77.8 (59.6-96.0) | 100.0 (99.9-100) | 86.7 (71.0-100) | 99.9 (99.9-100) |  |  |
|  |  | ICH, first-ever, women | 64.6 (56.5-72.6) | 99.9 (99.9-100) | 96.3 (92.4-100) | 99.3 (99.1-99.5) |  |  |
|  |  | ICH, first-ever or recurrent, men | 61.6 (45.3-78.0) | 100.0 (99.9-100) | 91.3 (79.8-100) | 99.8 (99.7-99.9) |  |  |
|  |  | ICH, first-ever, men | 55.2 (47.3-63.1) | 99.8 (99.7-99.9) | 89.9 (83.8-96.0) | 98.9 (98.6-99.2) |  |  |
| **Lakshminarayan**[54], 2014 | ICD-9 430.xx and 431.xx in any diagnostic position | haemorrhagic stroke, event-based analysis (7 day window) | 75.3 (65.0-83.4) | 99.9 (99.9-100) | 84.3 (74.3-91.1) | 99.9 (99.9-100) | 0.79 | High |
|  | ICD-9 430.xx and 431.xx in any diagnostic position | haemorrhagic stroke, person-based analysis (any time during follow-up) | 80.6 (70.9-87.8) | 99.9 (99.9-100) | 87.2 (77.9-93.1) | 99.9 (99.9-100) | 0.84 |  |
| **Leibson**[26], 1999 | ICD-9 431 (up to 5 diagnostic positions) | incident stroke |  |  | 73.91 (51-89) |  |  | High |
|  |  | incident or recurrent stroke |  |  | 86.96 (65-97) |  |  |  |
|  | ICD-9 432 (up to 5 diagnostic positions) | incident stroke |  |  | 0 (0-56) |  |  |  |
|  |  | incident or recurrent stroke |  |  | 0 (0-56) |  |  |  |
|  | ICD-9 431 and 432 (up to 5 diagnostic positions) | incident stroke |  |  | 56.67 (37.66-74.02) |  |  |  |
|  |  | incident or recurrent stroke |  |  | 66.67 (47.14-82.06) |  |  |  |
| **Leone**[10], 2004 | ICD-9 431, primary position | ICH | 56.52 (47.83-64.85) |  | 76.47 (66.84-84.06) |  |  | High |
|  | ICD-9 431, primary or secondary position |  | 59.42 (50.72-67.59) |  | 74.55 (65.19-82.16) |  |  |  |
|  | ICD-9 431 or 432, primary position |  | 57.97 (49.27-66.22) |  | 75.47 (65.98-83.08) |  |  |  |
|  | ICD-9 431 or 432, primary or secondary position |  | 60.87 (52.17-68.95) |  | 73.68 (64.46-81.28) |  |  |  |
|  | ICD-9 431 | for any stroke, primary position |  |  | 99.02 (93.88-99.95) |  |  |  |
|  | ICD-9 432 |  |  |  | 100 (39.58-100) |  |  |  |
|  | ICD-9 431 | for any stroke, primary or secondary position |  |  | 95.45 (89.20-98.31) |  |  |  |
|  | ICD-9 432 |  |  |  | 100 (39.58-100) |  |  |  |
|  | ICD-9 432 | for ICH, primary position | 1.45 (0.25-5.67) |  | 50 (9.19-90.81) |  |  |  |
|  |  | for ICH, primary or secondary position | 1.45 (0.25-5.67) |  | 50 (39.58-100) |  |  |  |
|  | ICD-9 431 and 432 | for any stroke, primary position |  |  | 99.06 (94.10-99.95) |  |  |  |
|  |  | for any stroke, primary or secondary position |  |  | 95.61 (89.56-98.37) |  |  |  |
| **Leppala**[42], 1999 | ICD-8 431.00,431.08,431.09,431.90,431.98,431.99 or ICD-9 431 | ICH, hospital discharge |  |  | 82.10 (62-93) |  |  | High |
|  |  | ICH. COD |  |  | 91.10 (80-97) |  |  |  |
|  |  | ICH, hospital discharge (diagnosis in hospital and vital statistics data) |  |  | 91.00 |  |  |  |
|  |  | ICH, COD (diagnosis in hospital discharge and vital statistics data) |  |  | 100 |  |  |  |
| **Lindblad**[43], 1993 | ICD-8&9 431-432 | ICH |  |  | 55.00 (32.05-76.17) |  |  | High |
|  |  | acute stroke |  |  | 95.00 (73.06-99.74) |  |  |  |
| **Liu**[27], 1999 | ICD-9 431, primary diagnosis | all hospitals combined, definite stroke |  |  | 73 (57-85) |  |  | High |
|  | ICD-9 432, primary diagnosis |  |  |  | 10 (2-32) |  |  |  |
|  | ICD-9 431, primary diagnosis | all hospitals combined, definite or highly-probable stroke |  |  | 88 (73-95) |  |  |  |
|  | ICD-9 432, primary diagnosis |  |  |  | 29 (12-52) |  |  |  |
|  | ICD-9 431 and 432, primary diagnosis | all hospitals combined, definite stroke |  |  | 51.61 (38.68-64.34) |  |  |  |
|  |  | all hospitals combined, definite or highly-probable stroke |  |  | 67.74 (54.54-78.73) |  |  |  |
|  | ICD-9 431, primary, secondary, or tertiary diagnosis | all hospitals combined, definite stroke |  |  | 73 (59-85) |  |  |  |
|  | ICD-9 432, primary, secondary, or tertiary diagnosis |  |  |  | 11 (3-30) |  |  |  |
|  | ICD-9 431, primary, secondary, or tertiary diagnosis | all hospitals combined, definite or highly-probable stroke |  |  | 86 (72-94) |  |  |  |
|  | ICD-9 432, primary, secondary, or tertiary diagnosis |  |  |  | 32 (17-52) |  |  |  |
|  | ICD-9 431 and 432, primary, secondary, or tertiary diagnosis | all hospitals combined, definite stroke |  |  | 54.05 (42.13-65.56) |  |  |  |
|  |  | all hospitals combined, definite or highly-probable stroke |  |  | 68.92 (56.96-78.89) |  |  |  |
| **Mayo**[44], 1993 | ICD-9 431 | acute stroke, neurologist #1 |  |  | 90.91 (57.12-99.52) |  |  | High |
|  |  | acute stroke, neurologist #2 |  |  | 100.00 (46.29-100) |  |  |  |
|  | ICD-9 432 | acute stroke, neurologist #1 |  |  | 100.00 (31.00-100) |  |  |  |
|  |  | acute stroke, neurologist #2 |  |  | 0.00 (0-69.00) |  |  |  |
|  | ICD-9 431, 432 | acute stroke, neurologist #1 |  |  | 92.86 (64.17-99.63) |  |  |  |
|  |  | acute stroke, neurologist #2 |  |  | 62.50 (25.89-89.76) |  |  |  |
| **Olson**[45], 2014 | ICD-9 431.xx, inpatient diagnosis | any stroke |  |  | 83 (71-91) |  |  | High |
|  | ICD-9 431.xx, outpatient diagnosis | any stroke |  |  | 84 (78-89) |  |  |  |
|  | ICD-9 431.xx, inpatient and outpatient diagnosis | any stroke |  |  | 91 (77-98) |  |  |  |
|  | ICD-9 432.xx, inpatient diagnosis | any stroke |  |  | 46 (32-59) |  |  |  |
|  | ICD-9 432.xx, outpatient diagnosis | any stroke |  |  | 37 (31-42) |  |  |  |
|  | ICD-9 432.xx, inpatient and outpatient diagnosis | any stroke |  |  | 41 (26-57) |  |  |  |
|  | ICD-9 431.xx, inpatient diagnosis | SAH or ICH |  |  | 79 (60-91) |  |  |  |
|  | ICD-9 431.xx, outpatient diagnosis | SAH or ICH |  |  | 45 (37-53) |  |  |  |
|  | ICD-9 431.xx, inpatient and outpatient diagnosis | SAH or ICH |  |  | 78 (60-90) |  |  |  |
|  | ICD-9 432.xx, inpatient diagnosis | SAH or ICH |  |  | 77 (56-91) |  |  |  |
|  | ICD-9 432.xx, outpatient diagnosis | SAH or ICH |  |  | 74 (65-82) |  |  |  |
|  | ICD-9 432.xx, inpatient and outpatient diagnosis | SAH or ICH |  |  | 71 (44-90) |  |  |  |
| **Palmieri**[28], 2007 | ICD-9 431 | non-fatal, primary diagnosis, men vs. nonfatal CV events (MONICA stroke categories definite or associated w/ definite MI) |  |  | 86 (81-90) |  |  | Medium |
|  |  | non-fatal, women, vs. nonfatal CV events (MONICA stroke categories definite or associated w/ definite MI) |  |  | 81 (73-87) |  |  |  |
|  |  | non-fatal total |  |  | 84 (80-88) |  |  |  |
|  |  | fatal men vs. fatal CV event (MONICA stroke categories definite, associate w/definite or possible MI, or unclassifiable) |  |  | 76 (66-84) |  |  |  |
|  |  | fatal women vs. fatal CV event (MONICA stroke categories definite, associate w/definite or possible MI, or unclassifiable) |  |  | 87 (75-94) |  |  |  |
|  |  | fatal total |  |  | 80 (73-86) |  |  |  |
|  | ICD-9 432 | non-fatal, primary diagnosis, men vs. nonfatal CV events (MONICA stroke categories definite or associated w/ definite MI) |  |  | 60 (48-71) |  |  |  |
|  |  | non-fatal, women, vs. nonfatal CV events (MONICA stroke categories definite or associated w/ definite MI) |  |  | 54 (34-73) |  |  |  |
|  |  | non-fatal total |  |  | 59 (48-68) |  |  |  |
|  |  | fatal men vs. fatal CV event (MONICA stroke categories definite, associate w/definite or possible MI, or unclassifiable) |  |  | 60 (17-93) |  |  |  |
|  |  | fatal women vs. fatal CV event (MONICA stroke categories definite, associate w/definite or possible MI, or unclassifiable) |  |  | 67 (13-98) |  |  |  |
|  |  | fatal total |  |  | 63 (26-90) |  |  |  |
|  | ICD-9 431, 432 | non-fatal, primary diagnosis, men vs. nonfatal CV events (MONICA stroke categories definite or associated w/ definite MI) |  |  | 80 (75-85) |  |  |  |
|  |  | non-fatal, women, vs. nonfatal CV events (MONICA stroke categories definite or associated w/ definite MI) |  |  | 77 (69-83) |  |  |  |
|  |  | non-fatal total |  |  | 79 (75-83) |  |  |  |
|  |  | fatal men vs. fatal CV event (MONICA stroke categories definite, associate w/definite or possible MI, or unclassifiable) |  |  | 75 (65-83) |  |  |  |
|  |  | fatal women vs. fatal CV event (MONICA stroke categories definite, associate w/definite or possible MI, or unclassifiable) |  |  | 86 (74-93) |  |  |  |
|  |  | fatal total |  |  | 79 (72-85) |  |  |  |
| **Phillips**[29], 1993 | ICD-9 431 as primary or secondary discharge diagnosis | acute stroke |  |  | 100.00 (5.46-100) |  |  | High |
|  | ICD-9 432 as primary or secondary discharge diagnosis |  |  |  | 0.00 (0-80.21) |  |  |  |
|  | ICD-9 431, 432 as primary or secondary discharge diagnosis |  |  |  | 33.33 (1.77-87.47) |  |  |  |
| **Reker**[46], 2001 | ICD-9 431.x discharge diagnosis | new stroke |  |  | 80 (59-92) |  |  | High |
|  | ICD-9 432.x discharge diagnosis | new stroke |  |  | 21 (9-40) |  |  |  |
| **Ramalle-Gomara**[30], 2013 | ICD-9-CM 430-432 | haemorrhagic stroke, primary diagnosis | 91.3 (77.6-100.0) | 98.1 (96.7-99.6) | 75.0 (57.2-92.9) | 99.5 (98.6-100.0) |  | High |
|  |  | haemorrhagic stroke, primary or secondary diagnosis | 91.3 (77.6-100.0) | 97.6 (96.0-99.3) | 70.0 (51.9-88.1) | 99.5 (98.6-100.0) |  |  |
| **Rao**[13], 2007 | ICD-10 I61 | ICH as underlying COD | 69 |  |  |  |  | High |
| **Rosamond**[32], 1999 | ICD-9-CM 431 | definite or probable stroke |  |  | 82.93 (67.35-92.30) |  |  | High |
|  | ICD-9-CM 432 |  |  |  | 9.09 (0.48-42.88) |  |  |  |
|  | ICD-9-CM 431, 432 |  |  |  | 67.31 (52.78-79.28) |  |  |  |
|  | ICD-9-CM 431 | definite or probable ICH |  |  | 63.41 (46.92-77.43) |  |  |  |
|  | ICD-9-CM 432 |  |  |  | 9.09 (0.48-42.88) |  |  |  |
|  | ICD-9-CM 431, 432 |  |  |  | 51.92 (37.78-65.78) |  |  |  |
| **Roumie**[47], 2008 | ICD-9 431 | ICH |  |  | 100 (69.87-100) |  |  | High |
| **Spolaore**[48], 2005 | ICD-9 431 | hospital discharge primary position vs. stroke (MONICA criteria definite or definite stroke associated w/definite MI) |  |  | 78 (74-83) |  |  | High |
|  | ICD-9 432 |  |  |  | 54 (52-55) |  |  |  |
| **Thigpen**[55], 2015 | ICD-9 430-432 | intracranial haemorrhage |  |  | 94.71 (92.13-96.51) |  |  | Medium |
| **Tirschwell**[49], 2002 | ICD-9-CM 431, up to nine discharge positions | ICH |  |  | 80 (64-91) |  |  | High |
|  | ICD-9-CM 431, first two discharge positions |  |  |  | 83 (67-93) |  |  |  |
|  | ICD-9-CM 431, primary discharge position only |  |  |  | 89 (75-97) |  |  |  |
| **Tolonen**[34], 2007 | ICD-9 431 or ICD-10 I61 | ICH, all cases | 95 (93-97) |  | 87 (84-90) |  |  | High |
|  |  | fatal | 95 (92-98) |  | 92 (88-96) |  |  |  |
|  |  | nonfatal | 94 (91-97) |  | 84 (80-88) |  |  |  |
|  |  | men | 94 (91-97) |  | 84 (79-89) |  |  |  |
|  |  | women | 95 (92-98) |  | 89 (85-93) |  |  |  |
|  | ICD-9 431 | ICH | 95 (92-98) |  | 87 (83-91) |  |  |  |
|  | ICD-10 I61.x |  | 94 (91-97) |  | 86 (82-90) |  |  |  |
| **ISCHAEMIC STROKE** | | | | | | | | |
| **Agrawal**[37], 2009 | ICD-9 433.x, inpatient diagnosis | ischaemic or haemorrhagic stroke |  |  | 38 (9-76) |  |  | Medium |
|  | ICD-9 433.x, outpatient diagnosis only |  |  |  | 0 (0-71) |  |  |  |
|  | ICD-9 433.x, inpatient or outpatient diagnosis |  |  |  | 27 (6-61) |  |  |  |
|  | ICD-9 434.x, inpatient diagnosis |  |  |  | 74 (55-88) |  |  |  |
|  | ICD-9 434.x, outpatient diagnosis only |  |  |  | 40 (5-85) |  |  |  |
|  | ICD-9 434.x, inpatient or outpatient diagnosis |  |  |  | 69 (52-84) |  |  |  |
|  | ICD-9 433.x or 434.x, inpatient diagnosis |  |  |  | 67 (50-80) |  |  |  |
|  | ICD-9 433.x or 434.x, outpatient diagnosis only |  |  |  | 25 (4-64) |  |  |  |
|  | ICD-9 433.x or 434.x, inpatient or outpatient diagnosis |  |  |  | 60 (44-73) |  |  |  |
|  | ICD-9 436, inpatient diagnosis |  |  |  | 46 (19-75) |  |  |  |
|  | ICD-9 436, outpatient diagnosis only |  |  |  | 19 (13-26) |  |  |  |
|  | ICD-9 436, inpatient or outpatient diagnosis |  |  |  | 21 (15-28) |  |  |  |
|  | ICD-9 434.x or 436.x, inpatient diagnosis |  |  |  | 66 (50-79) |  |  |  |
|  | ICD-9 434.x or 436.x, outpatient diagnosis only |  |  |  | 20 (14-26) |  |  |  |
|  | ICD-9 434.x or 436.x, inpatient or outpatient diagnosis |  |  |  | 29 (23-36) |  |  |  |
|  | ICD-9 433.x, 434.x, or 436 inpatient diagnosis |  |  |  | 62 (47-74) |  |  |  |
|  | ICD-9 433.x, 434.x, or 436 outpatient diagnosis only |  |  |  | 19 (14-26) |  |  |  |
|  | ICD-9 433.x, 434.x, or 436 inpatient or outpatient diagnosis |  |  |  | 29 (23-36) |  |  |  |
| **Benesch**[56], 1997 | ICD-9 433, primary discharge diagnosis | acute stroke |  |  | 9 (5-15) |  |  | High |
|  | ICD-9 434, primary discharge diagnosis |  |  |  | 90 (85-94) |  |  |  |
|  | ICD-9 436, primary discharge diagnosis |  |  |  | 89 (64-98) |  |  |  |
|  | ICD-9 433 and 434, primary discharge diagnosis |  |  |  | 59 (53.72-64.09) |  |  |  |
|  | ICD-9 434 and 436, primary discharge diagnosis |  |  |  | 90.15 (84.99-93.73) |  |  |  |
|  | ICD-9 433, 434, and 436, primary discharge diagnosis |  |  |  | 52.51 (47.35-57.61) |  |  |  |
|  | ICD-9 433, primary or secondary discharge diagnosis |  |  |  | 6 (4-10) |  |  |  |
|  | ICD-9 434, primary or secondary discharge diagnosis |  |  |  | 85 (79-89) |  |  |  |
|  | ICD-9 436, primary or secondary discharge diagnosis |  |  |  | 83 (64-93) |  |  |  |
|  | ICD-9 433 and 434, primary or secondary discharge diagnosis |  |  |  | 40.31 (36.09-44.67) |  |  |  |
|  | ICD-9 434 and 436, primary or secondary discharge diagnosis |  |  |  | 84.71 (79.56-88.78) |  |  |  |
|  | ICD-9 433, 434, and 436, primary or secondary discharge diagnosis |  |  |  | 42.55 (38.39-46.81) |  |  |  |
| **Broderick**[17], 1998 | ICD-9-CM 433, primary or secondary position | acute stroke |  |  | 34.62 (17.94-55.64) |  |  | High |
|  | ICD-9-CM 434, primary or secondary position |  |  |  | 80.21 (73.63-85.52) |  |  |  |
|  | ICD-9-CM 436, primary or secondary position |  |  |  | 64.10 (54.65-72.61) |  |  |  |
|  | ICD-9-CM 433 or 434, primary or secondary position |  |  |  | 74.65 (68.16-80.23) |  |  |  |
|  | ICD-9-CM 434 or 436, primary or secondary position |  |  |  | 74.01 (68.63-78.77) |  |  |  |
|  | ICD-9-CM 433, 434, or 436 primary or secondary position |  |  |  | 70.91 (65.63-75.69) |  |  |  |
| **Cheng**[57], 2011 | ICD-9-CM 433.xx, 434.xx | ischaemic stroke |  |  | 97.85 (95.64-99.00) |  |  | High |
| **Derby**[52], 2000 | ICD-9 434 | definite or probable stroke |  |  | 84 (82-86) |  |  | High |
| **Ellekjaer**[19], 1999 | ICD-9 433 | any stroke, first-ever |  |  | 40 (13.69-72.63) |  |  | High |
|  |  | any stroke, first or recurrent |  |  | 40 (13.69-72.63) |  |  |  |
|  | ICD-9 434 | any stroke, first-ever |  |  | 68.37 (62.86-73.42) |  |  |  |
|  |  | any stroke, first or recurrent |  |  | 85.94 (81.48-89.50) |  |  |  |
|  | ICD-9 436 | any stroke, first-ever |  |  | 67.42 (56.56-76.75) |  |  |  |
|  |  | any stroke, first or recurrent |  |  | 79.78 (69.66-87.26) |  |  |  |
|  | ICD-9 433 and 434 | any stroke, first-ever |  |  | 67.49 (62.05-72.52) |  |  |  |
|  |  | any stroke, first or recurrent |  |  | 84.52 (80.00-88.19) |  |  |  |
|  | ICD-9 434 and 436 | any stroke, first-ever |  |  | 68.16 (63.32-72.64) |  |  |  |
|  |  | any stroke, first or recurrent |  |  | 84.58 (80.59-87.89) |  |  |  |
|  | ICD-9 433, 434, 436 | any stroke, first-ever |  |  | 67.48 (62.68-71.94) |  |  |  |
|  |  | any stroke, first or recurrent |  |  | 83.50 (79.47-86.88) |  |  |  |
| **Goldstein**[58], 1998 | ICD-9-CM 433 | ischaemic stroke |  |  | 4.17 (0.73-15.43) |  |  | Medium |
|  | ICD-9-CM 434 |  |  |  | 82 (74-88) |  |  |  |
|  | ICD-9-CM 436 |  |  |  | 79 (54-93) |  |  |  |
|  | ICD-9-CM 434.11 |  |  |  | 85 (65-95) |  |  |  |
|  | ICD-9-CM 434.91 |  |  |  | 82 (72-90) |  |  |  |
|  | ICD-9-CM 43x.x1 (with cerebral infarction) |  |  |  | 79.20 (71.1-85.6) |  |  |  |
|  | ICD-9-CM 43x.x0 (without cerebral infarction) |  |  |  | 6.67 (1.74-19.31) |  |  |  |
|  | ICD-9-CM 433.x0 |  |  |  | 2.33 (0.12-13.80) |  |  |  |
|  | ICD-9-CM 433.x1 |  |  |  | 20.00 (1.05-70.12) |  |  |  |
|  | ICD-9-CM 434.x0 |  |  |  | 100 (19.79-100) |  |  |  |
|  | ICD-9-CM 434.x1 |  |  |  | 82.08 (73.17-88.60) |  |  |  |
|  | ICD-9-CM 433, 434, or 436 as primary hospital discharge diagnosis |  |  |  | 61 (53-68) |  |  |  |
| **Golomb**[59], 2006 | ICD-9 433 | any stroke, any position |  |  | 79 (49-94) |  |  | Medium |
|  | ICD-9 434 |  |  |  | 62 (55-68) |  |  |  |
|  | ICD-9 436 | any stroke, any position |  |  | 88.46 (75.87-95.22) |  |  |  |
|  |  | any stroke, primary position only |  |  | 92.11 (77.52-97.94) |  |  |  |
|  | ICD-9 433, 436 | any stroke, any position |  |  | 85.71 (74.10-92.86) |  |  |  |
|  | ICD-9 433, 434 |  |  |  | 62.50 (56.42-68.22) |  |  |  |
|  | ICD-9 434, 436 |  |  |  | 66.13 (60.53-71.32) |  |  |  |
|  | ICD-9 433, 434, 436 |  |  |  | 66.67 (61.21-71.72) |  |  |  |
|  | ICD-9 433 | arterial ischaemic stroke, any position |  |  | 79 (49-94) |  |  |  |
|  | ICD-9 434 | arterial ischaemic stroke, any position |  |  | 52 (46-58) |  |  |  |
|  | ICD-9 436 | arterial ischaemic stroke, any position |  |  | 83 (69-91) |  |  |  |
|  |  | arterial ischaemic stroke, primary position only |  |  | 86.84 (71.11-95.05) |  |  |  |
|  | ICD-9 433, 436 | arterial ischaemic stroke, any position |  |  | 80.95 (68.71-89.36) |  |  |  |
|  | ICD-9 433, 434 |  |  |  | 53.31 (47.19-59.33) |  |  |  |
|  | ICD-9 434, 436 |  |  |  | 57.10 (51.37-62.64) |  |  |  |
|  | ICD-9 433, 434, 436 |  |  |  | 58.02 (52.43-63.42) |  |  |  |
| **Haesebaert**[60], 2013 | ICD-10 I63, primary position | ischaemic stroke | 67.31 (62.81-71.52) |  | 95.14 (92.06-97.10) |  |  | High |
|  |  | ischaemic stroke, among patients at stroke unit | 80.33 (71.95-86.75) |  | 96.08 (89.69-98.74) |  |  |  |
| **Heckbert**[39], 2004 | ICD-9 434 | any stroke |  |  | 85 (82-89) |  |  | High |
|  | ICD-9 436 | any stroke |  |  | 70 (62-78) |  |  |  |
|  | ICD-9 434 and 436 | any stroke |  |  | 81.93 (  78.47-84.95  ) |  |  |  |
| **Hsieh**[61], 2013 | ICD-9-CM 433.xx or 434.xx | ischaemic stroke | 97.34 (96.38-98.05) |  | 88.36 (86.74-89.82) |  |  | High |
| **Iso**[20], 1990 | ICD-8 432-434, ICD-9 433-434 | stroke as underlying COD on death certificate |  |  | 100 (92.26-100) |  |  | Medium |
|  |  | non-haemorrhagic/occlusive stroke as COD on death certificate | 43.24 (31.94-55.25) |  | 91.47 (84.91-95.45) |  |  |  |
| **Ives**[21], 1995 | ICD-9-CM 434 | incident stroke |  |  | 91.94 (82.17-97.33) |  |  | High |
|  | ICD-9-CM-436 |  |  |  | 77.78 (39.99-97.19) |  |  |  |
|  | ICD-9-CM 434 and 436 |  |  |  | 90.14 (80.74-95.94) |  |  |  |
| **Johnsen**[22], 2002 | ICD-10 I63 | ischaemic stroke |  |  | 87.60 (80.1-93.1) |  |  | High |
|  | ICD-10 I64 |  |  |  | 76 (69-82) |  |  |  |
| **Jones**[23], 2014 | ICD-9-CM 433, any position | any stroke, definite or probable |  |  | 10 (8-12) |  |  | High |
|  | ICD-9-CM 433, primary position |  |  |  | 15.82 (13-19) |  |  |  |
|  | ICD-9-CM 434, any position |  |  |  | 77 (74-79) |  |  |  |
|  | ICD-9-CM 434, primary position |  |  |  | 83.22 (81-86) |  |  |  |
|  | ICD-9-CM 433 and 434, any position |  |  |  | 41.56 (39.53-43.62) |  |  |  |
|  | ICD-9-CM 433 and 434, primary position |  |  |  | 54.86 (52.30-57.39) |  |  |  |
|  | ICD-9-CM 436, any position |  |  |  | 72 (66-77) |  |  |  |
|  | ICD-9-CM 436, primary position |  |  |  | 82.14 (76-87) |  |  |  |
|  | ICD-9-CM 434 and 436, any position |  |  |  | 75.60 (73.22-77.83) |  |  |  |
|  | ICD-9-CM, 434 and 436, primary position |  |  |  | 83.00 (80.61-85.15) |  |  |  |
|  | ICD-9-CM 433/434/436, any position |  |  |  | 44.94 (43.01-46.89) |  |  |  |
|  | ICD-9-CM 433/434/436, primary position |  |  |  | 58.40 (56.03-60.73) |  |  |  |
|  | ICD-9-CM 433.01, 433.11, 433.21, 433.31, 433.81, 433.91, 434.01, 434.11, 434.91 (AHA/ASA code group), any position | ischaemic stroke |  |  | 76 (74-79) |  |  |  |
|  |  | ischaemic stroke, males |  |  | 78 (74-81) |  |  |  |
|  |  | ischaemic stroke, females |  |  | 75 (71-79) |  |  |  |
|  |  | ischaemic stroke, incident |  |  | 80 (76-83) |  |  |  |
|  | ICD-9-CM 433.01, 433.11, 433.21, 433.31, 433.81, 433.91, 434.01, 434.11, 434.91 (AHA/ASA code group), primary position only | ischaemic stroke |  |  | 82 (79-84) |  |  |  |
|  | ICD-9-CM 433.01, 433.11, 433.21, 433.31, 433.81, 433.91, 434, 434.01, 434.11, 434.91, 436 (alternative code group), any position | ischaemic stroke |  |  | 75 (73-77) |  |  |  |
|  |  | ischaemic stroke, males |  |  | 77 (73-80) |  |  |  |
|  |  | ischaemic stroke, females |  |  | 74 (70-77) |  |  |  |
|  |  | ischaemic stroke, incident |  |  | 79 (76-81) |  |  |  |
|  | ICD-9-CM 433.01, 433.11, 433.21, 433.31, 433.81, 433.91, 434, 434.01, 434.11, 434.91, 436 (alternative code group), primary position only | ischaemic stroke |  |  | 81 (79-84) |  |  |  |
| **Kokotailo**[41], 2005 | ICD-9 362.3, 433.x1, 434.x1, 436 | arterial ischaemic stroke |  |  | 85 (78-90) |  |  | Medium |
|  | ICD-10 H34.1, I63.x, I64.x |  |  |  | 85 (76-92) |  |  |  |
| **Krarup**[25], 2007 | ICD-10 I63 | ischaemic stroke, reviewer #1 |  |  | 97 (84-100) |  |  | High |
|  |  | ischaemic stroke, reviewer #2 |  |  | 100 (89-100) |  |  |  |
| **Kumamaru**[53], 2014 | ICD-9-CM 433.x1, 434.x1, or 436, primary discharge diagnosis | ischaemic stroke, first-ever or recurrent | 58.6 (53.6-63.6) | 99.8 (99.7-99.9) | 88.6 (84.7-92.6) | 99.0 (98.8-99.1) |  | High |
|  |  | ischaemic stroke, first-ever | 58.6 (52.4-64.7) | 99.9 (99.8-100) | 91.1 (86.6-95.5) | 99.2 (99.1-99.4) |  |  |
|  |  | ischaemic stroke, first-ever or recurrent, women | 62.6 (55.4-69.8) | 99.9 (99.8-100) | 92.0 (87.1-96.9) | 99.2 (98.9-99.4) |  |  |
|  |  | ischaemic stroke, first-ever, women | 74.2 (50.3-98.2) | 100.0 (99.9-100) | 79.2 (56.2-100) | 100.0 (99.9-100) |  |  |
|  |  | ischaemic stroke, first-ever or recurrent, men | 55.2 (48.3-62.2) | 99.7 (99.6-99.9) | 85.8 (87.1-96.9) | 98.8 (98.5-99.0) |  |  |
|  |  | ischaemic stroke, first-ever, men | 53.3 (34.8-71.7) | 100.0 (99.9-100) | 88.2 (72.9-100) | 99.8 (99.7-99.9) |  |  |
| **Lakshminarayan**[54], 2014 | ICD-9 433.x1, 434.x1, 436.xx in any diagnostic position | ischaemic stroke, event-based analysis (7 day window) | 82.2 (78.3-85.5) | 99.7 (99.6-99.7) | 79.5 (75.5-82.9) | 99.7 (99.7-99.8) | 0.81 | High |
|  | ICD-9 433.x1, 434.x1, 436.xx in any diagnostic position | ischaemic stroke, person-based analysis (any time during follow-up) | 88.4 (85.1-91.1) | 99.6 (99.6-99.7) | 82.7 (79.1-85.9) | 99.8 (99.7-99.8) | 0.85 |  |
| **Leibson**[26], 1999 | ICD-9 433 (up to 5 diagnostic positions) | incident stroke |  |  | 15 (4-39) |  |  | High |
|  |  | incident or recurrent stroke |  |  | 15 (4-39) |  |  |  |
|  | ICD-9 434 (up to 5 diagnostic positions) | incident stroke |  |  | 68.63 (59-77) |  |  |  |
|  |  | incident or recurrent stroke |  |  | 85.29 (77-91) |  |  |  |
|  | ICD-9 433, 434 (up to 5 diagnostic positions) | incident stroke |  |  | 59.84 (50.55-68.49) |  |  |  |
|  |  | incident or recurrent stroke |  |  | 73.77 (64.89-81.12) |  |  |  |
|  | ICD-9 436 (up to 5 diagnostic positions) | incident stroke |  |  | 67 (54-78) |  |  |  |
|  |  | incident or recurrent stroke |  |  | 86 (74-93) |  |  |  |
|  | ICD-9 434, 436 (up to 5 diagnostic positions) | incident stroke |  |  | 68.07 (60.33-74.96) |  |  |  |
|  |  | incident or recurrent stroke |  |  | 85.54 (79.05-90.34) |  |  |  |
|  | ICD-9 433, 434, 436 (up to 5 diagnostic positions) | incident stroke |  |  | 62.37 (54.95-69.27) |  |  |  |
|  |  | incident or recurrent stroke |  |  | 77.96 (71.18-83.55) |  |  |  |
| **Leone**[10], 2004 | ICD-9 433 | ischaemic stroke, primary or secondary diagnosis | 2 (0.8-3) |  | 5.97 (2.80-11.81) |  |  | High |
|  | ICD-9 434 |  | 37 (33-41) |  | 87.00 (82-91) |  |  |  |
|  | ICD-9 433, 434 |  | 38 (34-43) |  | 54.76 (49.27-60.15) |  |  |  |
|  | ICD-9 434, 436 |  | 45 (41-50) |  | 83.40 (78.17-87.60) |  |  |  |
|  | ICD-9 433, 434, 436 |  | 47 (42-52) |  | 57.00 (51.93-61.93) |  |  |  |
|  | ICD-9 433 | any stroke (SAH, CH, ischaemic, or stroke unknown), primary or secondary position |  |  | 7.46 (3.84-13.65) |  |  |  |
|  | ICD-9 434 |  |  |  | 91.09 (86.07-94.48) |  |  |  |
|  | ICD-9 433, 434 |  |  |  | 57.74 (52.25-63.05) |  |  |  |
|  | ICD-9 434, 436 |  |  |  | 90.35 (85.92-93.54) |  |  |  |
|  | ICD-9 433, 434, 436 |  |  |  | 62.09 (57.07-66.87) |  |  |  |
|  | ICD-9 433 | ischaemic stroke, primary diagnosis | 1 (0.64-3) |  | 7.87 (3.49-16.05) |  |  |  |
|  | ICD-9 434 |  | 35 (31-40) |  | 89.89 (84.45-93.65) |  |  |  |
|  | ICD-9 433, 434 |  | 37 (33-41) |  | 63.54 (57.54-69.16) |  |  |  |
|  | ICD-9 434, 436 |  | 43 (38-47) |  | 86.86 (81.72-90.77) |  |  |  |
|  | ICD-9 433, 434, 436 |  | 44 (40-49) |  | 65.23 (59.75-70.35) |  |  |  |
|  | ICD-9 433 | any stroke (SAH, CH, ischaemic, or stroke unknown), primary diagnosis |  |  | 8.99 (4.24-17.43) |  |  |  |
|  | ICD-9 434 |  |  |  | 94.15 (89.50-96.89) |  |  |  |
|  | ICD-9 436 |  |  |  | 88 (76-95) |  |  |  |
|  | ICD-9 433, 434 |  |  |  | 66.79 (60.86-72.24) |  |  |  |
|  | ICD-9 434, 436 |  |  |  | 94.07 (90.03-96.59) |  |  |  |
|  | ICD-9 433, 434, 436 |  |  |  | 70.77 (65.45-75.59) |  |  |  |
| **Leppala**[42], 1999 | ICD-8 432,433,434 or ICD-9 433,434 (excluding ICD-9 4330X, 4331X,4339X,4349X) | ischaemic stroke, hospital discharge |  |  | 90.10 (86-93) |  |  | High |
|  |  | ischaemic stroke, COD |  |  | 92.40 (84-97) |  |  |  |
|  |  | ischaemic stroke, hospital discharge (diagnosis in hospital and vital statistics data) |  |  | 90.90 |  |  |  |
|  |  | ischaemic stroke, COD (diagnosis in hospital and vital statistics data) |  |  | 100 |  |  |  |
| **Lindblad**[43], 1993 | ICD-8&9 433 | any stroke |  |  | 100 (31.00-100) |  |  | High |
|  |  | ischaemic stroke |  |  | 33.33 (1.77-87.47) |  |  |  |
|  | ICD-8&9 434 | any stroke |  |  | 92.77 (84.35-97.03) |  |  |  |
|  |  | ischaemic stroke |  |  | 75.90 (65.04-84.33) |  |  |  |
|  | ICD-8&9 436 | any stroke |  |  | 95.08 (89.15-97.99) |  |  |  |
|  |  | ischaemic stroke |  |  | 19.67 (13.25-28.05) |  |  |  |
|  | ICD-8&9 433, 434 | any stroke |  |  | 93.02 (84.84-97.13) |  |  |  |
|  |  | ischaemic stroke |  |  | 74.42 (63.67-82.95) |  |  |  |
|  | ICD-8&9 434, 436 | any stroke |  |  | 94.15 (89.75-96.80) |  |  |  |
|  |  | ischaemic stroke |  |  | 42.44 (35.64-49.53) |  |  |  |
|  | ICD-8&9 433, 434, 436 | any stroke |  |  | 94.23 (89.89-95.85) |  |  |  |
|  |  | ischaemic stroke |  |  | 42.31 (36.56-49.34) |  |  |  |
| **Liu**[27], 1999 | ICD-9 433, primary | definite stroke |  |  | 14 (5-31) |  |  | High |
|  | ICD-9 434, primary |  |  |  | 70 (63-76) |  |  |  |
|  | ICD-9 434.1, primary |  |  |  | 43 (19-70) |  |  |  |
|  | ICD-9 436, primary |  |  |  | 29 (24-35) |  |  |  |
|  | ICD-9 433, 434, primary |  |  |  | 60.41 (53.96-66.52) |  |  |  |
|  | ICD-9 434, 436, primary |  |  |  | 45.71 (41.25-50.24) |  |  |  |
|  | ICD-9 433, 434, 436, primary |  |  |  | 43.62 (39.35-47.99) |  |  |  |
|  | ICD-9 433, primary | definite or highly-probable stroke |  |  | 17 (7-34) |  |  |  |
|  | ICD-9 434, primary |  |  |  | 85 (79-90) |  |  |  |
|  | ICD-9 434.1, primary |  |  |  | 79 (49-94) |  |  |  |
|  | ICD-9 436, primary |  |  |  | 85 (81-89) |  |  |  |
|  | ICD-9 433, 434, primary |  |  |  | 75.10 (69.11-80.29) |  |  |  |
|  | ICD-9 434, 436, primary |  |  |  | 85.10 (81.57-88.07) |  |  |  |
|  | ICD-9 433, 434, 436, primary |  |  |  | 80.57 (76.87-83.82) |  |  |  |
|  | ICD-9 433, primary/secondary/tertiary | definite stroke |  |  | 9 (4-21) |  |  |  |
|  | ICD-9 434, primary/secondary/tertiary |  |  |  | 68 (61-74) |  |  |  |
|  | ICD-9 434.1, primary/secondary/tertiary |  |  |  | 32 (14-57) |  |  |  |
|  | ICD-9 436, primary/secondary/tertiary |  |  |  | 26 (21-31) |  |  |  |
|  | ICD-9 433, 434, primary/secondary/tertiary |  |  |  | 54.88 (49.03-60.61) |  |  |  |
|  | ICD-9 434, 436, primary/secondary/tertiary |  |  |  | 41.79 (37.80-45.90) |  |  |  |
|  | ICD-9 433, 434, 436, primary/secondary/tertiary |  |  |  | 39.13 (35.36-43.03) |  |  |  |
|  | ICD-9 433, primary/secondary/tertiary | definite or highly-probable stroke |  |  | 15 (7-28) |  |  |  |
|  | ICD-9 434, primary/secondary/tertiary |  |  |  | 82 (76-87) |  |  |  |
|  | ICD-9 434.1, primary/secondary/tertiary |  |  |  | 58 (34-79) |  |  |  |
|  | ICD-9 436, primary/secondary/tertiary |  |  |  | 81 (76-85) |  |  |  |
|  | ICD-9 433, 434, primary/secondary/tertiary |  |  |  | 68.69 (63.03-73.85) |  |  |  |
|  | ICD-9 434, 436, primary/secondary/tertiary |  |  |  | 80.54 (77.07-83.61) |  |  |  |
|  | ICD-9 433, 434, 436, primary/secondary/tertiary |  |  |  | 75.16 (71.59-78.41) |  |  |  |
| **Mayo**[44], 1993 | ICD-9 433 | acute stroke, neurologist #1 |  |  | 100.00 (62.88-100) |  |  | High |
|  |  | acute stroke, neurologist #2 |  |  | 50.00 (9.19-90.81) |  |  |  |
|  | ICD-9 434 | acute stroke, neurologist #1 |  |  | 100.00 (80.76-100) |  |  |  |
|  |  | acute stroke, neurologist #2 |  |  | 95.00 (73.06-99.74) |  |  |  |
|  | ICD-9 436 | acute stroke, neurologist #1 |  |  | 83.33 (68.04-92.49) |  |  |  |
|  |  | acute stroke, neurologist #2 |  |  | 61.54 (40.73-79.09) |  |  |  |
|  | ICD-9 433, 434 | acute stroke, neurologist #1 |  |  | 100.00 (85.87-100) |  |  |  |
|  |  | acute stroke, neurologist #2 |  |  | 87.50 (66.54-96.71) |  |  |  |
|  | ICD-9 434, 436 | acute stroke, neurologist #1 |  |  | 85.71 (73.22-93.20) |  |  |  |
|  |  | acute stroke, neurologist #2 |  |  | 76.09 (60.90-86.92) |  |  |  |
|  | ICD-9 433, 434, 436 | acute stroke, neurologist #1 |  |  | 90.28 (80.42-95.67) |  |  |  |
|  |  | acute stroke, neurologist #2 |  |  | 74.00 (59.39-84.92) |  |  |  |
| **Olson**[45], 2014 | ICD-9 433.xx, inpatient diagnosis | any stroke |  |  | 62 (53-70) |  |  | High |
|  | ICD-9 433.xx, outpatient diagnosis |  |  |  | 37 (34-41) |  |  |  |
|  | ICD-9 433.xx, inpatient and outpatient diagnosis |  |  |  | 54 (44-62) |  |  |  |
|  | ICD-9 433.x1, inpatient diagnosis |  |  |  | 94 (83-99) |  |  |  |
|  | ICD-9 433.x1, outpatient diagnosis |  |  |  | 92 (74-99) |  |  |  |
|  | ICD-9 433.x1, inpatient and outpatient diagnosis |  |  |  | 100 (54-100) |  |  |  |
|  | ICD-9 433.x0, inpatient diagnosis |  |  |  | 47 (36-57) |  |  |  |
|  | ICD-9 433.x0, outpatient diagnosis |  |  |  | 36 (33-40) |  |  |  |
|  | ICD-9 433.x0, inpatient and outpatient diagnosis |  |  |  | 44 (33-54) |  |  |  |
|  | ICD-9 434.xx, inpatient diagnosis |  |  |  | 94 (90-96) |  |  |  |
|  | ICD-9 434.xx, outpatient diagnosis |  |  |  | 89 (87-90) |  |  |  |
|  | ICD-9 434.xx, inpatient and outpatient diagnosis |  |  |  | 97 (94-90) |  |  |  |
|  | ICD-9 434.x1, inpatient diagnosis |  |  |  | 94 (91-96) |  |  |  |
|  | ICD-9 434.x1, outpatient diagnosis |  |  |  | 88 (86-90) |  |  |  |
|  | ICD-9 434.x1, inpatient and outpatient diagnosis |  |  |  | 97 (94-90) |  |  |  |
|  | ICD-9 434.x0, inpatient diagnosis |  |  |  | 50 (7-93) |  |  |  |
|  | ICD-9 434.x0, outpatient diagnosis |  |  |  | 92 (74-99) |  |  |  |
|  | ICD-9 434.x0, inpatient and outpatient diagnosis |  |  |  | 0 |  |  |  |
|  | ICD-9 436.xx, inpatient diagnosis |  |  |  | 89 (73-97) |  |  |  |
|  | ICD-9 436.xx, outpatient diagnosis |  |  |  | 84 (81-86) |  |  |  |
|  | ICD-9 436.xx, inpatient and outpatient diagnosis |  |  |  | 93 (77-99) |  |  |  |
|  | ICD-9 433.xx, inpatient diagnosis | ischaemic stroke |  |  | 87 (78-93) |  |  |  |
|  | ICD-9 433.xx, outpatient diagnosis |  |  |  | 83 (79-88) |  |  |  |
|  | ICD-9 433.xx, inpatient and outpatient diagnosis |  |  |  | 86 (75-94) |  |  |  |
|  | ICD-9 433.x1, inpatient diagnosis |  |  |  | 93 (82-99) |  |  |  |
|  | ICD-9 433.x1, outpatient diagnosis |  |  |  | 87 (66-97) |  |  |  |
|  | ICD-9 433.x1, inpatient and outpatient diagnosis |  |  |  | 94 (75-98) |  |  |  |
|  | ICD-9 433.x0, inpatient diagnosis |  |  |  | 81 (70-93) |  |  |  |
|  | ICD-9 433.x0, outpatient diagnosis |  |  |  | 84 (79-88) |  |  |  |
|  | ICD-9 433.x0, inpatient and outpatient diagnosis |  |  |  | 81 (68-94) |  |  |  |
|  | ICD-9 434.xx, inpatient diagnosis |  |  |  | 84 (80-88) |  |  |  |
|  | ICD-9 434.xx, outpatient diagnosis |  |  |  | 74 (72-77) |  |  |  |
|  | ICD-9 434.xx, inpatient and outpatient diagnosis |  |  |  | 84 (79-89) |  |  |  |
|  | ICD-9 434.x1, inpatient diagnosis |  |  |  | 84 (80-88) |  |  |  |
|  | ICD-9 434.x1, outpatient diagnosis |  |  |  | 74 (72-77) |  |  |  |
|  | ICD-9 434.x1, inpatient and outpatient diagnosis |  |  |  | 84 (79-89) |  |  |  |
|  | ICD-9 434.x0, inpatient diagnosis |  |  |  | 100 (16-100) |  |  |  |
|  | ICD-9 434.x0, outpatient diagnosis |  |  |  | 65 (46-85) |  |  |  |
|  | ICD-9 434.x0, inpatient and outpatient diagnosis |  |  |  | 0 |  |  |  |
|  | ICD-9 436.xx, inpatient diagnosis |  |  |  | 87 (70-96) |  |  |  |
|  | ICD-9 436.xx, outpatient diagnosis |  |  |  | 68 (64-71) |  |  |  |
|  | ICD-9 436.xx, inpatient and outpatient diagnosis |  |  |  | 85 (66-96) |  |  |  |
| **Palmieri**[28], 2007 | 433, primary discharge diagnosis | nonfatal CV event, men (MONICA stroke categories definite or associated w/ definite MI) |  |  | 12 (8-16) |  |  | Medium |
|  | 434, primary discharge diagnosis |  |  |  | 81 (77-83) |  |  |  |
|  | 436, primary discharge diagnosis |  |  |  | 61 (56-66) |  |  |  |
|  | 433, primary discharge diagnosis | nonfatal CV event, women (MONICA stroke categories definite or associated w/ definite MI) |  |  | 9 (6-14) |  |  |  |
|  | 434, primary discharge diagnosis |  |  |  | 78 (74-81) |  |  |  |
|  | 436, primary discharge diagnosis |  |  |  | 67 (61-72) |  |  |  |
|  | 433, primary discharge diagnosis | nonfatal CV event, men and women (MONICA stroke categories definite or associated w/ definite MI) |  |  | 10.69 (8.26-13.71) |  |  |  |
|  | 434, primary discharge diagnosis |  |  |  | 79.51 (77.07-81.75) |  |  |  |
|  | 436, primary discharge diagnosis |  |  |  | 63.43 (59.52-67.18) |  |  |  |
|  | 433 | fatal CV event, men (MONICA stroke categories definite, associate w/definite or possible MI, or unclassifiable) |  |  | 100 (20-100) |  |  |  |
|  | 434 |  |  |  | 83 (69-92) |  |  |  |
|  | 436 |  |  |  | 74 (65-82) |  |  |  |
|  | 433 | fatal CV event, women (MONICA stroke categories definite, associate w/definite or possible MI, or unclassifiable) |  |  | 100 (31-100) |  |  |  |
|  | 434 |  |  |  | 89 (71-97) |  |  |  |
|  | 436 |  |  |  | 71 (60-80) |  |  |  |
|  | 433 | fatal CV event, men and women (MONICA stroke categories definite, associate w/definite or possible MI, or unclassifiable) |  |  | 100 (46.29-100) |  |  |  |
|  | 434 |  |  |  | 85.33 (74.85-92.10) |  |  |  |
|  | 436 |  |  |  | 72.82 (65.91-78.81) |  |  |  |
|  | 433 and 434 | nonfatal CV event, men |  |  | 58.65 (55.61-61.62) |  |  |  |
|  |  | nonfatal CV event, women |  |  | 57.23 (38.94-46.68) |  |  |  |
|  |  | nonfatal CV event, men and women (MONICA stroke categories definite or associated w/ definite MI) |  |  | 58.11 (55.73-60.45) |  |  |  |
|  | 433 and 434 | fatal CV event, men |  |  | 83.67 (69.80-92.20) |  |  |  |
|  |  | fatal CV event, women |  |  | 90.32 (73.01-97.47) |  |  |  |
|  |  | fatal CV event, men and women (MONICA stroke categories definite, associate w/definite or possible MI, or unclassifiable) |  |  | 86.25 (76.31-92.61) |  |  |  |
|  | 434 and 436 | nonfatal CV event, men |  |  | 74.03 (71.30-76.60) |  |  |  |
|  |  | nonfatal CV event, women |  |  | 73.76 (70.36-76.90) |  |  |  |
|  |  | nonfatal, men and women |  |  | 73.92 (71.82-75.92) |  |  |  |
|  | 434 and 436 | fatal CV event, men |  |  | 76.73 (69.24-82.90) |  |  |  |
|  |  | fatal CV event, women |  |  | 75.68 (66.44-83.10) |  |  |  |
|  |  | fatal, men and women |  |  | 76.30 (70.68-81.15) |  |  |  |
|  | 433, 434, and 436 | nonfatal CV event, men |  |  | 59.20 (56.59-61.76) |  |  |  |
|  |  | nonfatal CV event, women |  |  | 60.07 (56.81-63.24) |  |  |  |
|  |  | nonfatal CV event, men and women (MONICA stroke categories definite or associated w/ definite MI) |  |  | 59.54 (57.52-61.53) |  |  |  |
|  | 433, 434, and 436 | fatal CV event, men |  |  | 77.02 (69.60-83.12) |  |  |  |
|  |  | fatal CV event, women |  |  | 76.32 (67.26-83.56) |  |  |  |
|  |  | fatal CV event, men and women (MONICA stroke categories definite, associate w/definite or possible MI, or unclassifiable) |  |  | 76.73 (71.19-81.50) |  |  |  |
| **Phillips**[29], 1993 | ICD-9 433 as primary or secondary discharge diagnosis | acute stroke |  |  | 11.54 (4.78-24.13) |  |  | High |
|  | ICD-9 434 as primary or secondary discharge diagnosis |  |  |  | 64.29 (35.63-86.02) |  |  |  |
|  | ICD-9 436 as primary or secondary discharge diagnosis |  |  |  | 66.10 (56.74-74.40) |  |  |  |
|  | ICD-9 433, 434 as primary or secondary discharge diagnosis |  |  |  | 22.73 (13.68-34.99) |  |  |  |
|  | ICD-9 434, 436 as primary or secondary discharge diagnosis |  |  |  | 65.91 (57.09-73.79) |  |  |  |
|  | ICD-9 433, 434, 436 as primary or secondary discharge diagnosis |  |  |  | 50.54 (43.12-57.95) |  |  |  |
| **Ramalle-Gomara**[30], 2013 | ICD-9-CM 433&434, primary diagnosis | ischaemic stroke | 84.1 (76.7-91.5) | 92.8 (89.7-96.0) | 81.1 (73.3-88.8) | 94.1 (91.2-97.0) |  | High |
|  | ICD-9-CM 433&434, primary or secondary diagnosis |  | 84.1 (76.7-91.5) | 84.3 (80.0-88.6) | 66.2 (57.9-74.5) | 93.6 (90.4-96.7) |  |  |
| **Rao**[13], 2007 | ICD-10 I63 | cerebral infarction as underlying COD | 66 |  |  |  |  | High |
| **Reker**[46], 2001 | ICD-9 433.x0 discharge diagnosis | new stroke |  |  | 13 (2-40) |  |  | High |
|  | ICD-9 433.x1 discharge diagnosis |  |  |  | 71 (30-95) |  |  |  |
|  | ICD-9 434.x0 discharge diagnosis |  |  |  | 33 (6-76) |  |  |  |
|  | ICD-9 434.x1 discharge diagnosis |  |  |  | 72 (64-79) |  |  |  |
|  | ICD-9 436.x discharge diagnosis |  |  |  | 48 (39-58) |  |  |  |
| **Rinaldi**[62], 2003 | ICD-9 434, 1st diagnostic level | ischaemic stroke |  |  | 100 (31-100) |  |  | High |
|  | ICD-9 434, all diagnostic levels |  |  |  | 100 (31-100) |  |  |  |
|  | ICD-9 436, 1st diagnostic level |  |  |  | 75 (68-82) |  |  |  |
|  | ICD-9 436, all diagnostic levels |  |  |  | 71 (63-77) |  |  |  |
|  | ICD-9 434 and 436, 1st diagnostic level |  |  |  | 76 (68-82) |  |  |  |
|  | ICD-9 434 and 436, all diagnostic levels |  |  |  | 71 (64-77) |  |  |  |
| **Rosamond**[32],1999 | ICD-9-CM 433 | definite or probable stroke |  |  | 13.53 (9.78-18.38) |  |  | High |
|  | ICD-9-CM 434 |  |  |  | 77.42 (70.60-83.08) |  |  |  |
|  | ICD-9-CM 436 |  |  |  | 70.37 (60.70-78.57) |  |  |  |
|  | ICD-9-CM 433, 434 |  |  |  | 39.82 (35.31-44.52) |  |  |  |
|  | ICD-9-CM 434, 436 |  |  |  | 74.83 (69.39-79.61) |  |  |  |
|  | ICD-9-CM 433, 434, 436 |  |  |  | 45.71 (41.54-49.94) |  |  |  |
|  | ICD-9-CM 433 | definite or probable ischaemic stroke |  |  | 13.53 (9.78-18.38) |  |  |  |
|  | ICD-9-CM 434 |  |  |  | 76.88 (70.03-82.60) |  |  |  |
|  | ICD-9-CM 436 |  |  |  | 67.59 (57.82-76.10) |  |  |  |
|  | ICD-9-CM 433, 434 |  |  |  | 39.60 (35.09-44.29) |  |  |  |
|  | ICD-9-CM 434, 436 |  |  |  | 73.47 (67.97-78.35) |  |  |  |
|  | ICD-9-CM 433, 434, 436 |  |  |  | 45.00 (40.84-49.23) |  |  |  |
| **Roumie**[47], 2008 | ICD-9 433.x1, 434 (not 434.x0), 436 | ischaemic stroke |  |  | 84.67 (77.67-89.84) |  |  | High |
| **Spolaore**[48], 2005 | ICD-9 433 | acute stroke |  |  | 9 (6-13) |  |  | High |
|  | ICD-9 434 |  |  |  | 77 (73-82) |  |  |  |
|  | ICD-9 436 |  |  |  | 61 (56-67) |  |  |  |
| **Thigpen**[55], 2015 | ICD-9 433 | ischaemic stroke |  |  | 85.25 (73.33-92.62) |  |  | Medium |
|  | ICD-9 434 |  |  |  | 94.39 (92.94-95.56) |  |  |  |
|  | ICD-9 436 |  |  |  | 50.00 (2.67-97.33) |  |  |  |
|  | ICD-9 433, 434 |  |  |  | 93.97 (92.51-95.16) |  |  |  |
|  | ICD-9 434, 436 |  |  |  | 94.32 (92.86-95.50) |  |  |  |
|  | ICD-9 433, 434, 436 |  |  |  | 93.96 (92.53-95.14) |  |  |  |
| **Tirschwell**[49], 2002 | ICD-9-CM 433.x1, 434 (excluding 434.x0), 436, up to nine discharge positions | ischaemic stroke |  |  | 90 (77-97) |  | 0.82 | High |
|  | ICD-9-CM 433.x1, 434 (excluding 434.x0), 436, first two discharge positions |  |  |  | 91 (78-97) |  | 0.78 |  |
|  | ICD-9-CM 433.x1, 434 (excluding 434.x0), 436, primary discharge position only |  |  |  | 88 (74-96) |  | 0.72 |  |
| **Tolonen**[34], 2007 | ICD-9 433, 434, 436, or ICD-10 I63,I64 | cerebral infarction, all cases | 81 (80-82) |  | 83 (82-84) |  |  | High |
|  | ICD-9 433, 434 or ICD-10 I63 |  | 80 (78-82) |  | 82 (81-83) |  |  |  |
|  | ICD-9 433, 434, 436, or ICD-10 I63,I64 | fatal | 79 (75-83) |  | 83 (80-86) |  |  |  |
|  | ICD-9 433, 434 or ICD-10 I63 |  | 76 (72-80) |  | 77 (73-81) |  |  |  |
|  | ICD-9 433, 434, 436, or ICD-10 I63,I64 | nonfatal | 82 (80-84) |  | 84 (83-85) |  |  |  |
|  | ICD-9 433, 434 or ICD-10 I63 |  | 81 (79-83) |  | 83 (81-85) |  |  |  |
|  | ICD-9 433, 434, 436, or ICD-10 I63,I64 | men | 80 (78-82) |  | 85 (83-87) |  |  |  |
|  | ICD-9 433, 434 or ICD-10 I63 |  | 79 (77-81) |  | 84 (82-86) |  |  |  |
|  | ICD-9 433, 434, 436, or ICD-10 I63,I64 | women | 82 (80-84) |  | 82 (80-84) |  |  |  |
|  | ICD-9 433, 434 or ICD-10 I63 |  | 81 (79-83) |  | 80 (78-82) |  |  |  |
|  | ICD-9 433,434, 436 | cerebral infarction | 84 (82-86) |  | 84 (82-86) |  |  |  |
|  | ICD-9 433,434 |  | 82 (80-84) |  | 82 (80-84) |  |  |  |
|  | ICD-10 I63,I64 |  | 79 (77-81) |  | 83 (81-85) |  |  |  |
|  | ICD-10 I63 |  | 78 (76-80) |  | 82 (80-84) |  |  |  |
| **Wahl**[63], 2010 | ICD-9 433.x1,434.x1 | ischaemic stroke w/o TIA |  |  | 95.50 (91.0-98.2) |  |  | High |
|  | ICD-9 433.x1,434.x1, 436.xx, 437.1x, 437.9x |  |  |  | 87.4 (82.0-91.7) |  |  |  |

95% CI=95% confidence interval; COD=cause-of-death; CV=cardiovascular; ICD=International Classification of diseases; ICH=intracerebral haemorrhage; MI=myocardial infarction; NPV=negative predictive value; PABAK=prevalence-adjusted bias-adjusted kappa; PPV=positive predictive value; SAH=subsrachnoid haemorrhage; TIA=transient ischaemic attack

1. Borzecki AM, Wong AT, Hickey EC, Ash AS, Berlowitz DR. Identifying hypertension-related comorbidities from administrative data: what’s the optimal approach? Am J Med Qual Off J Am Coll Med Qual. 2004;19: 201–206.

2. Chen G, Faris P, Hemmelgarn B, Walker RL, Quan H. Measuring agreement of administrative data with chart data using prevalence unadjusted and adjusted kappa. BMC Med Res Methodol. 2009;9: 5–2288–9–5.

3. de Faire U, Friberg L, Lorich U, Lundman T. A validation of cause-of-death certification in 1,156 deaths. Acta Med Scand. 1976;200: 223–228.

4. Hasan M, Meara RJ, Bhowmick BK. The quality of diagnostic coding in cerebrovascular disease. Int J Qual Health Care J Int Soc Qual Health Care ISQua. 1995;7: 407–410.

5. Henderson T, Shepheard J, Sundararajan V. Quality of diagnosis and procedure coding in ICD-10 administrative data. Med Care. 2006;44: 1011–1019.

6. Hennessy DA, Quan H, Faris PD, Beck CA. Do coder characteristics influence validity of ICD-10 hospital discharge data? BMC Health Serv Res. 2010;10: 99. doi:10.1186/1472-6963-10-99

7. Humphries KH, Rankin JM, Carere RG, Buller CE, Kiely FM, Spinelli JJ. Co-morbidity data in outcomes research: are clinical data derived from administrative databases a reliable alternative to chart review? J Clin Epidemiol. 2000;53: 343–349.

8. Lambert L, Blais C, Hamel D, Brown K, Rinfret S, Cartier R, et al. Evaluation of care and surveillance of cardiovascular disease: can we trust medico-administrative hospital data? Can J Cardiol. 2012;28: 162–168. doi:10.1016/j.cjca.2011.10.005

9. Lee DS, Donovan L, Austin PC, Gong Y, Liu PP, Rouleau JL, et al. Comparison of coding of heart failure and comorbidities in administrative and clinical data for use in outcomes research. Med Care. 2005;43: 182–188.

10. Leone MA, Capponi A, Varrasi C, Tarletti R, Monaco F. Accuracy of the ICD-9 codes for identifying TIA and stroke in an Italian automated database. Neurol Sci Off J Ital Neurol Soc Ital Soc Clin Neurophysiol. 2004;25: 281–288. doi:10.1007/s10072-004-0355-8

11. Levy AR, Tamblyn RM, Fitchett D, McLeod PJ, Hanley JA. Coding accuracy of hospital discharge data for elderly survivors of myocardial infarction. Can J Cardiol. 1999;15: 1277–1282.

12. Rampatige R, Gamage S, Peiris S, Lopez AD. Assessing the reliability of causes of death reported by the Vital Registration System in Sri Lanka: medical records review in Colombo. HIM J. 2013;42: 20–28.

13. Rao C, Yang G, Hu J, Ma J, Xia W, Lopez AD. Validation of cause-of-death statistics in urban China. Int J Epidemiol. 2007;36: 642–651. doi:10.1093/ije/dym003

14. Singh B, Singh A, Ahmed A, Wilson GA, Pickering BW, Herasevich V, et al. Derivation and validation of automated electronic search strategies to extract Charlson comorbidities from electronic medical records. Mayo Clin Proc. 2012;87: 817–824. doi:10.1016/j.mayocp.2012.04.015

15. So L, Evans D, Quan H. ICD-10 coding algorithms for defining comorbidities of acute myocardial infarction. BMC Health Serv Res. 2006;6: 161.

16. Soo M, Robertson LM, Ali T, Clark LE, Fluck N, Johnston M, et al. Approaches to ascertaining comorbidity information: validation of routine hospital episode data with clinician-based case note review. BMC Res Notes. 2014;7: 253. doi:10.1186/1756-0500-7-253

17. Broderick J, Brott T, Kothari R, Miller R, Khoury J, Pancioli A, et al. The Greater Cincinnati/Northern Kentucky Stroke Study: preliminary first-ever and total incidence rates of stroke among blacks. Stroke J Cereb Circ. 1998;29: 415–421.

18. Brown DL, Senani F Al-, Lisabeth LD, Farnie MA, Colletti LA, Langa KM, et al. Defining cause of death in stroke patients: The Brain Attack Surveillance in Corpus Christi Project. Am J Epidemiol. 2007;165: 591–596. doi:10.1093/aje/kwk042

19. Ellekjaer H, Holmen J, Krüger O, Terent A. Identification of incident stroke in Norway: hospital discharge data compared with a population-based stroke register. Stroke J Cereb Circ. 1999;30: 56–60.

20. Iso H, Jacobs DR Jr, Goldman L. Accuracy of death certificate diagnosis of intracranial hemorrhage and nonhemorrhagic stroke. The Minnesota Heart Survey. Am J Epidemiol. 1990;132: 993–998.

21. Ives DG, Fitzpatrick AL, Bild DE, Psaty BM, Kuller LH, Crowley PM, et al. Surveillance and ascertainment of cardiovascular events. The Cardiovascular Health Study. Ann Epidemiol. 1995;5: 278–285.

22. Johnsen SP, Overvad K, Sørensen HT, Tjønneland A, Husted SE. Predictive value of stroke and transient ischemic attack discharge diagnoses in The Danish National Registry of Patients. J Clin Epidemiol. 2002;55: 602–607.

23. Jones SA, Gottesman RF, Shahar E, Wruck L, Rosamond WD. Validity of hospital discharge diagnosis codes for stroke: the Atherosclerosis Risk in Communities Study. Stroke J Cereb Circ. 2014;45: 3219–3225. doi:10.1161/STROKEAHA.114.006316

24. Klatsky AL, Friedman GD, Sidney S, Kipp H, Kubo A, Armstrong MA. Risk of hemorrhagic stroke in Asian American ethnic groups. Neuroepidemiology. 2005;25: 26–31. doi:10.1159/000085310

25. Krarup L-H, Boysen G, Janjua H, Prescott E, Truelsen T. Validity of stroke diagnoses in a National Register of Patients. Neuroepidemiology. 2007;28: 150–154. doi:10.1159/000102143

26. Leibson CL, Naessens JM, Brown RD, Whisnant JP. Accuracy of hospital discharge abstracts for identifying stroke. Stroke J Cereb Circ. 1994;25: 2348–2355.

27. Liu L, Reeder B, Shuaib A, Mazagri R. Validity of stroke diagnosis on hospital discharge records in Saskatchewan, Canada: implications for stroke surveillance. Cerebrovasc Dis Basel Switz. 1999;9: 224–230. doi:15960

28. Palmieri L, Barchielli A, Cesana G, de Campora E, Goldoni CA, Spolaore P, et al. The Italian register of cardiovascular diseases: attack rates and case fatality for cerebrovascular events. Cerebrovasc Dis Basel Switz. 2007;24: 530–539. doi:10.1159/000110423

29. Phillips S, Cameron K, Chung C. Stroke surveillance revisited. Can J Cardiol. 1993;9: 124D.

30. Ramalle-Gomara E, Ruiz E, Serrano M, Bartulos M, Gonzalez M-A, Matute B. Validity of Discharge Diagnoses in the Surveillance of Stroke. Neuroepidemiology. 2013;41: 185–188. doi:10.1159/000354626

31. Reggio A, Failla G, Patti F. Reliability of death certificates in the study of stroke mortality. A retrospective study in a Sicilian municipality. Ital J Neurol Sci. 1995;16: 567–570.

32. Rosamond WD, Folsom AR, Chambless LE, Wang CH, McGovern PG, Howard G, et al. Stroke incidence and survival among middle-aged adults: 9-year follow-up of the Atherosclerosis Risk in Communities (ARIC) cohort. Stroke J Cereb Circ. 1999;30: 736–743.

33. Sinha S, Myint PK, Luben RN, Khaw K-T. Accuracy of death certification and hospital record linkage for identification of incident stroke. BMC Med Res Methodol. 2008;8: 74. doi:10.1186/1471-2288-8-74

34. Tolonen H, Salomaa V, Torppa J, Sivenius J, Immonen-Räihä P, Lehtonen A, et al. The validation of the Finnish Hospital Discharge Register and Causes of Death Register data on stroke diagnoses. Eur J Cardiovasc Prev Rehabil Off J Eur Soc Cardiol Work Groups Epidemiol Prev Card Rehabil Exerc Physiol. 2007;14: 380–385. doi:10.1097/01.hjr.0000239466.26132.f2

35. Wildenschild C, Mehnert, Frank, W. Thomsen R, Iversen H, Vestergaard K, Ingeman, Annette A, et al. Registration of acute stroke: validity in the Danish Stroke Registry and the Danish National Registry of Patients. Clin Epidemiol. 2013; 27. doi:10.2147/CLEP.S50449

36. Wu C-S, Lai M-S, Gau SS-F, Wang S-C, Tsai H-J. Concordance between patient self-reports and claims data on clinical diagnoses, medication use, and health system utilization in Taiwan. PloS One. 2014;9: e112257. doi:10.1371/journal.pone.0112257

37. Agrawal N, Johnston SC, Wu YW, Sidney S, Fullerton HJ. Imaging data reveal a higher pediatric stroke incidence than prior US estimates. Stroke J Cereb Circ. 2009;40: 3415–3421. doi:10.1161/STROKEAHA.109.564633

38. Gaist D, Vaeth M, Tsiropoulos I, Christensen K, Corder E, Olsen J, et al. Risk of subarachnoid haemorrhage in first degree relatives of patients with subarachnoid haemorrhage: follow up study based on national registries in Denmark. BMJ. 2000;320: 141–145.

39. Heckbert SR, Kooperberg C, Safford MM, Psaty BM, Hsia J, McTiernan A, et al. Comparison of self-report, hospital discharge codes, and adjudication of cardiovascular events in the Women’s Health Initiative. Am J Epidemiol. 2004;160: 1152–1158. doi:10.1093/aje/

40. Kirkman MA, Mahattanakul W, Gregson BA, Mendelow AD. The accuracy of hospital discharge coding for hemorrhagic stroke. Acta Neurol Belg. 2009;109: 114–119.

41. Kokotailo RA, Hill MD. Coding of stroke and stroke risk factors using international classification of diseases, revisions 9 and 10. Stroke J Cereb Circ. 2005;36: 1776–1781. doi:10.1161/01.STR.0000174293.17959.a1

42. Leppälä JM, Virtamo J, Heinonen OP. Validation of stroke diagnosis in the National Hospital Discharge Register and the Register of Causes of Death in Finland. Eur J Epidemiol. 1999;15: 155–160.

43. Lindblad U, Råstam L, Ranstam J, Peterson M. Validity of register data on acute myocardial infarction and acute stroke: the Skaraborg Hypertension Project. Scand J Soc Med. 1993;21: 3–9.

44. Mayo N, Danys I, Carlton J, Scott S. Accuracy of hospital discharge coding for stroke. Can J Cardiol. 1993;9: 121D.

45. Olson KL, Wood MD, Delate T, Lash LJ, Rasmussen J, Denham AM, et al. Positive predictive values of ICD-9 codes to identify patients with stroke or TIA. Am J Manag Care. 2014;20: e27–34.

46. Reker DM, Hamilton BB, Duncan PW, Yeh SC, Rosen A. Stroke: who’s counting what? J Rehabil Res Dev. 2001;38: 281–289.

47. Roumie CL, Mitchel E, Gideon PS, Varas-Lorenzo C, Castellsague J, Griffin MR. Validation of ICD-9 codes with a high positive predictive value for incident strokes resulting in hospitalization using Medicaid health data. Pharmacoepidemiol Drug Saf. 2008;17: 20–26. doi:10.1002/pds.1518

48. Spolaore P, Brocco S, Fedeli U, Visentin C, Schievano E, Avossa F, et al. Measuring accuracy of discharge diagnoses for a region-wide surveillance of hospitalized strokes. Stroke J Cereb Circ. 2005;36: 1031–1034. doi:10.1161/01.STR.0000160755.94884.4a

49. Tirschwell DL, Longstreth WT Jr. Validating administrative data in stroke research. Stroke J Cereb Circ. 2002;33: 2465–2470.

50. Aboa-Eboulé C, Mengue D, Benzenine E, Hommel M, Giroud M, Béjot Y, et al. How accurate is the reporting of stroke in hospital discharge data? A pilot validation study using a population-based stroke registry as control. J Neurol. 2013;260: 605–613. doi:10.1007/s00415-012-6686-0

51. Arnason T, Wells PS, van Walraven C, Forster AJ. Accuracy of coding for possible warfarin complications in hospital discharge abstracts. Thromb Res. 2006;118: 253–262. doi:10.1016/j.thromres.2005.06.015

52. Derby CA, Lapane KL, Feldman HA, Carleton RA. Trends in Validated Cases of Fatal and Nonfatal Stroke, Stroke Classification, and Risk Factors in Southeastern New England, 1980 to 1991 : Data From the Pawtucket Heart Health Program. Stroke. 2000;31: 875–881. doi:10.1161/01.STR.31.4.875

53. Kumamaru H, Judd SE, Curtis JR, Ramachandran R, Hardy NC, Rhodes JD, et al. Validity of claims-based stroke algorithms in contemporary Medicare data: reasons for geographic and racial differences in stroke (REGARDS) study linked with medicare claims. Circ Cardiovasc Qual Outcomes. 2014;7: 611–619. doi:10.1161/CIRCOUTCOMES.113.000743

54. Lakshminarayan K, Larson JC, Virnig B, Fuller C, Allen NB, Limacher M, et al. Comparison of Medicare Claims Versus Physician Adjudication for Identifying Stroke Outcomes in the Women’s Health Initiative. Stroke. 2014;45: 815–821. doi:10.1161/STROKEAHA.113.003408

55. Thigpen JL, Dillon C, Forster KB, Henault L, Quinn EK, Tripodis Y, et al. Validity of international classification of disease codes to identify ischemic stroke and intracranial hemorrhage among individuals with associated diagnosis of atrial fibrillation. Circ Cardiovasc Qual Outcomes. 2015;8: 8–14. doi:10.1161/CIRCOUTCOMES.113.000371

56. Benesch C, Witter DM Jr, Wilder AL, Duncan PW, Samsa GP, Matchar DB. Inaccuracy of the International Classification of Diseases (ICD-9-CM) in identifying the diagnosis of ischemic cerebrovascular disease. Neurology. 1997;49: 660–664.

57. Cheng C-L, Kao Y-HY, Lin S-J, Lee C-H, Lai ML. Validation of the National Health Insurance Research Database with ischemic stroke cases in Taiwan. Pharmacoepidemiol Drug Saf. 2011;20: 236–242. doi:10.1002/pds.2087

58. Goldstein LB. Accuracy of ICD-9-CM coding for the identification of patients with acute ischemic stroke: effect of modifier codes. Stroke J Cereb Circ. 1998;29: 1602–1604.

59. Golomb MR, Garg BP, Saha C, Williams LS. Accuracy and yield of ICD-9 codes for identifying children with ischemic stroke. Neurology. 2006;67: 2053–2055. doi:10.1212/01.wnl.0000247281.98094.e2

60. Haesebaert J, Termoz A, Polazzi S, Mouchoux C, Mechtouff L, Derex L, et al. Can hospital discharge databases be used to follow ischemic stroke incidence? Stroke J Cereb Circ. 2013;44: 1770–1774. doi:10.1161/STROKEAHA.113.001300

61. Hsieh C-Y, Chen C-H, Li C-Y, Lai M-L. Validating the diagnosis of acute ischemic stroke in a National Health Insurance claims database. J Formos Med Assoc Taiwan Yi Zhi. 2013; doi:10.1016/j.jfma.2013.09.009

62. Rinaldi R, Vignatelli L, Galeotti M, Azzimondi G, de Carolis P. Accuracy of ICD-9 codes in identifying ischemic stroke in the General Hospital of Lugo di Romagna (Italy). Neurol Sci Off J Ital Neurol Soc Ital Soc Clin Neurophysiol. 2003;24: 65–69. doi:10.1007/s100720300074

63. Wahl PM, Rodgers K, Schneeweiss S, Gage BF, Butler J, Wilmer C, et al. Validation of claims-based diagnostic and procedure codes for cardiovascular and gastrointestinal serious adverse events in a commercially-insured population. Pharmacoepidemiol Drug Saf. 2010;19: 596–603. doi:10.1002/pds.1924
