# Supplementary material for: Validity of Diagnostic Codes for Acute Stroke in Administrative Databases: A Systematic Review
Source: PLoS One. 2015 Aug 20;10(8):e0135834. doi: 10.1371/journal.pone.0135834 (PMC4546158; doi:10.1371/journal.pone.0135834)
Supplement: S3 Table — (DOC) [file pone.0135834.s009.doc]

**S3 Table. Results of Studies Validating Sets of Diagnostic Codes for Stroke** in Administrative Data.

| **First Author, Year** | **Diagnostic Codes** | **Parameter** | **Sensitivity**  **(95% CI)** | **Specificity**  **(95% CI)** | **PPV**  **(95% CI)** | | **NPV**  **(95% CI)** | | **Kappa**  **(95% CI)** | **Quality** |
| --- | --- | --- | --- | --- | --- | --- | --- | --- | --- | --- |
| **Aboa-Eboule**[1], 2013 | ICD-10 I61, I63, I64, and G46 | acute stroke | 77.07 (73.98-79.89) |  | 69.21 (66.07-72.19) | |  |  | | High |
| **Agrawal**[2], 2009 | ICD-9 433.xx, 434.xx, 435.xx, 436, 437.x, 438.x; inpatient or outpatient diagnosis | ischaemic stroke | 34 (26-42) |  | 29 (23-34) | |  |  | | Medium |
|  | ICD-9 433.xx, 434.xx, 435.xx, 436, 437.x, 438.x; inpatient diagnosis |  | 21 (14-29) |  | 77 (65-86) | |  |  | |  |
|  | ICD-9 433.xx, 434.xx, 435.xx, 436, 437.x, 438.x; outpatient diagnosis only |  | 13 (5-15) |  | 14 (9.8-20) | |  |  | |  |
|  | ICD-9 430, 431, 433.x, 434.x, 435.x, 436, 437; inpatient diagnosis | ischaemic or haemorrhagic stroke |  |  | 73 (65-80) | |  |  | |  |
|  | ICD-9 430, 431, 433.x, 434.x, 435.x, 436, 437; outpatient diagnosis only |  |  |  | 26 (21-31) | |  |  | |  |
|  | ICD-9 430, 431, 433.x, 434.x, 435.x, 436, 437; inpatient or outpatient diagnosis |  |  |  | 43 (38-48) | |  |  | |  |
| **Appelros**[3], 2011 | ICD-10 I61, I63, I64 | hospitalization or vital statistics data | 88.33 (84.55-91.31) |  | 91.74 (88.29-94.27) | |  |  | | High |
|  |  | vital statistics data |  |  | 79.59 (70.00-86.68) | |  |  | |  |
| **Arnason**[4], 2006 | ICD-9-CM 433-436 | stroke or TIA |  |  | 57 (50-64) | |  |  | | High |
| **Birman-Deych**[5], 2005 | ICD-9-CM 433.x1, 434.x1, 435.x, 436, 437.1x, 437.9x, 438.x for ischaemic stroke/TIA | any position in hospital claims, as a current or past event | 35.00 (33.85-36.17) | 99.00 (98.84-99.14) | 93.05 (91.96-94.00) | | 79.93 (79.39-80.47) |  | | Medium |
|  |  | primary position, during baseline hospitalization (current event) | 18 | 99 |  | |  |  | |  |
|  |  | any position, during baseline hospitalization (current event) | 35 | 99.5 |  | |  |  | |  |
|  |  | any position, during baseline hospitalization (current event), excluding patients with stroke code in a prior hospitalization | 30 | 99.5 |  | |  |  | |  |
|  |  | any position, during 12 months prior to baseline hospitalization and including baseline | 58 | 96 |  | |  |  | |  |
| **Broderick**[6], 1998 | ICD-9-CM 430-436, primary or secondary position | acute stroke |  |  | 72 | |  |  | | High |
|  | ICD-9-CM 430-436, primary position only |  |  |  | 83 | |  |  | |  |
| **Davenport**[7], 1996 | ICD-9 431-434 (excluding 432.0/1), 436-438 in the principal diagnostic position | acute stroke (excluding SAH) | 86.30 (83.26-88.87) |  | 94.97 (92.73-96.57) | |  |  | | Medium |
| **Ellekjaer**[8], 1999 | ICD-9 430, 431, 434, 436 (all positions) | acute stroke vs. all hospitalized register cases | 89.20 (85.58-92.02) |  | 68.31 (64.04-72.30) | |  |  | | High |
| **Ghia**[9], 2010 | ICD-10 163.0-9, I64, I61.8, I61.9, I62.9 | stroke including cerebral haemorrhage or infarction | 85 (77-90) | 95 (93-97) | 85.93 (79-91) | | 95 (92-97) |  | | High |
|  | ICD-10 163.0-9, I64, I61.8, I61.9, I62.9, I65, I66 | stroke including cerebral haemorrhage or infarction, occlusion and stenosis of precerebral and/or cerebral arteries not resulting in cerebral infarction | 86 (79-91) | 91 (88-94) | 78.00 (70-84) | | 95 (92-97) |  | |  |
| **Heckbert**[10], 2014 | ICD-9 430, 431, 432.0-432.1, 432.9, 434, 436 | any stroke | 82 (79-85) | 99.6 (99.5-99.7) | 81 (78-84) | | 99.6 (99.5-99.7) | 0.81 (0.79-0.83) | | High |
| **Holick**[11], 2009 | ICD-9 430.xx-432.xx, 434.xx, 436.xx | acute stroke |  |  | 31.82 (24.14-40.57) | |  |  | | High |
| **Ives**[12], 1995 | ICD-9-CM 430, 431, 432, 434, 436 (identified from Medicare database only) | incident stroke | 81.03 (68.59-90.13) |  |  | |  |  | | High |
|  | ICD-9-CM 430, 431, 432, 434, 436 (identified from any source) |  |  |  | 89.87 (81.02-95.53) | |  |  | |  |
| **Kokotailo**[13], 2005 | ICD-9: 430.x, 431.x, 433.x, 434.x1, 436, 362.3 (all hospital sites) | stroke overall (not including TIA) |  |  | 90.60 (86-94) | |  |  | | Medium |
|  | ICD-10: I60.x, I61.x, I63.x, I64.x, H34.1 (all hospital sites) |  |  |  | 91.10 (86-95) | |  |  | |  |
| **Koster**[14], 2013 | ICD-10 I61, I63, I64 as primary or secondary hospital diagnosis | definite or possible stroke, incident or recurrent |  |  | 80.51 (78.71-82.20) | |  |  | | High |
|  |  | definite or possible stroke, incident |  |  | 85.83 (83.89-87.58) | |  |  | |  |
|  |  | definite or possible stroke or unclassifiable, incident or recurrent |  |  | 88.14 (86.64-89.50) | |  |  | |  |
|  |  | definite or possible stroke or unclassifiable, incident |  |  | 94.04 (92.65-95.19) | |  |  | |  |
|  | ICD-10 I61, I63, I64 as COD | definite or possible stroke, incident or recurrent |  |  | 25.81 (20.86-31.43) | |  |  | |  |
|  |  | definite or possible stroke, incident |  |  | 39.22 (29.85-49.41) | |  |  | |  |
|  |  | definite or possible stroke or unclassifiable, incident or recurrent |  |  | 62.00 (56.00-67.67) | |  |  | |  |
|  |  | definite or possible stroke or unclassifiable, incident |  |  | 87.25 (78.84-92.77) | |  |  | |  |
|  | ICD-10 I61, I63, I64 as primary or secondary hospital diagnosis or COD | definite or possible stroke, incident or recurrent |  |  | 73.91 (72.06-75.68) | |  |  | |  |
|  |  | definite or possible stroke, incident |  |  | 82.72 (80.71-84.57) | |  |  | |  |
|  |  | definite or possible stroke or unclassifiable, incident or recurrent |  |  | 84.98 (83.45-86.40) | |  |  | |  |
|  |  | definite or possible stroke or unclassifiable, incident |  |  | 93.59 (92.21-94.74) | |  |  | |  |
| **Kumamaru**[15], 2014 | ICD-9-CM 430, 431, 433.x1, 434.x1, or 436, primary discharge diagnosis | ICH or ischaemic stroke, first-ever or recurrent | 60.4 (55.8-65.1) | 99.8 (99.6-99.9) | 90.5 (87.1-94.0) | | 98.9 (98.7-99.0) |  | | High |
|  |  | ICH or ischaemic stroke, first-ever | 59.5 (53.8-65.1) | 99.9 (99.8-100) | 92.6 (88.8-96.4) | | 99.1 (98.9-99.3) |  | |  |
|  |  | ICH or ischaemic stroke, first-ever or recurrent, women | 64.2 (57.5-71.0) | 99.9 (99.8-100) | 93.1 (88.8-97.4) | | 99.1 (98.9-99.3) |  | |  |
|  |  | ICH or ischaemic stroke, first-ever, women | 74.2 (50.3-98.2) | 100 (99.9-100) | 79.2 (56.2-100) | | 100 (99.9-100) |  | |  |
|  |  | ICH or ischaemic stroke, first-ever or recurrent, men | 57.3 (50.9-63.7) | 99.8 (99.6-99.9) | 88.3 (83.1-93.5) | | 98.6 (98.4-98.9) |  | |  |
|  |  | ICH or ischaemic stroke, first-ever, men | 53.3 (34.8-71.7) | 100 (99.9-100) | 88.2 (72.9-100) | | 99.8 (99.7-99.9) |  | |  |
| **Lakshminarayan**[16], 2009 | ICD-9 431, 432, 434, 436, 437; any diagnostic position | acute stroke, WHO definition |  |  | 61.89 (60.65-63.11) | |  |  | | High |
|  |  | acute stroke, MSS definition |  |  | 42.52 (41.27-43.78) | |  |  | |  |
|  |  | acute stroke, confirmed through neuroimaging |  |  | 52.68 (51.13-54.21) | |  |  | |  |
|  | ICD-9 431, 432, 434, 436, 437; primary diagnostic position | acute stroke, WHO definition |  |  | 98.34 (97.87-98.71) | |  |  | |  |
|  |  | acute stroke, MSS definition |  |  | 67.57 (66.05-69.05) | |  |  | |  |
| **Lakshminarayan**[17], 2014 | ICD-9 430.xx, 431.xx, 433.x1, 434.x1, 436.xx in any diagnostic position | acute stroke, event-based analysis (7 day window) | 82.0 (78.6-85.0) | 99.7 (99.7-99.8) | 84.6 (81.3-87.4) | | 99.7 (99.6-99.7) | 0.83 | | High |
|  | ICD-9 430.xx, 431.xx, 433.x1, 434.x1, 436.xx in any diagnostic position | acute stroke, person-based analysis (any time during follow-up) | 88.0 (85.0-90.4) | 99.7 (99.6-99.8) | 87.5 (84.5-90.1) | | 99.7 (99.6-99.8) | 0.87 | |  |
|  | ICD-9 430.xx, 431.xx, 433.x1, 434.x1, 436.xx primary position only | acute stroke, event-based analysis (7 day window) | 75.3 (71.6-78.7) | 99.8 (99.7-99.8) | 87.5 (84.2-90.2) | | 99.5 (99.4-99.6) | 0.81 | |  |
| **Leibson**[18], 1999 | ICD-9 CM 430,431,433,434,436,437 (first three diagnostic codes) | stroke (hospitalized cases only) | 87.88 (81.67-92.26) |  |  | |  |  | | High |
|  |  | incident stroke |  |  | 63.51 (56.77-69.78) | |  |  | |  |
|  |  | incident or recurrent stroke |  |  | 79.28 (73.23-84.29) | |  |  | |  |
|  | ICD-9 430.xx, 431.xx, 433.x1, 434.x1, 436.xx primary position only | acute stroke, person-based analysis (any time during follow-up) | 81.0 (77.5-84.0) | 99.8 (99.7-99.8) | 89.6 (86.6-92.0) | | 99.5 (99.4-99.6) | 0.85 | |  |
| **Lentine**[19], 2009 | ICD-9-CM 430, 431, 432, 433.x1, 434.x1, 435.x, 997.02 in Medicare Part A claims (institutional) | stroke or TIA | 75.0 (53.8-96.2) |  |  | |  |  | | Medium |
|  | ICD-9-CM 430, 431, 432, 433.x1, 434.x1, 435.x, 997.02 in Medicare Part B claims (physician/supplier) | stroke or TIA | 81.3 (62.1-100) |  |  | |  |  | |  |
|  | ICD-9-CM 430, 431, 432, 433.x1, 434.x1, 435.x, 997.02 in Medicare Part A or Part B claims (institutional or physician/supplier) | stroke or TIA | 87.5 (71.3-100) |  |  | |  |  | |  |
|  | ICD-9-CM 430, 431, 432, 433.x1, 434.x1, 435.x, 997.02 one Part A or two Part B claims at least one day (but no more than 365 days) apart | stroke or TIA | 87.5 (71.3-100) |  |  | |  |  | |  |
| **Leone**[20], 2004 | ICD-9 430,431,434,436 | any stroke | 53 (49-57) |  | 90.00 (87-93) | |  |  | | High |
| **Leppala**[21], 1999 | ICD-8 or ICD-9 430; ICD-8 431.00,431.08,431.09,431.90,431.98,431.99 or ICD-9 431; ICD-8 432,433,434 or ICD-9 433,434 (excluding ICD-9 4330X, 4331X,4339X,4349X) | any stroke (SAH, ICH, ischameic, unspecified), definite, as hospital discharge diagnosis |  |  | 54.91 (49.33-60.37) | |  |  | | High |
|  |  | any stroke (SAH, ICH, ischaemic, unspecified), definite or probable, as hospital discharge diagnosis |  |  | 89.88 (85.95-92.83) | |  |  | |  |
|  |  | incident stroke, definite, as hospital discharge diagnosis |  |  | 80.98 (76.21-85.01) | |  |  | |  |
|  |  | any stroke (SAH, ICH, ischaemic, unspecified), definite, as COD |  |  | 77.00 (70.43-82.51) | |  |  | |  |
|  |  | any stroke (SAH, ICH, ischaemic, unspecified), definite or probable, as COD |  |  | 97.00 (93.28-98.77) | |  |  | |  |
|  |  | any stroke (SAH, ICH, ischaemic unspecified) in hospital and vital statistics data, as hospital discharge diagnosis |  |  | 97.56 (85.59-99.87) | |  |  | |  |
|  |  | any stroke (SAH, ICH, ischaemic, unspecified) in hospital and vital statistics data, definite or probable, as COD |  |  | 97.5 | |  |  | |  |
| **Lindblad**[22], 1993 | ICD-8&9 430-434, 436 | hospital diagnosis |  |  | 94.02 (90.14-96.50) | |  |  | | High |
|  |  | COD |  |  | 92.31 (78.03-97.99) | |  |  | |  |
| **Mayo**[23], 1993 | ICD-9 430-434, 436, 437 | acute stroke, neurologist #1 |  |  | 80.46 (70.28-87.89) | |  |  | | High |
|  |  | acute stroke, neurologist #2 |  |  | 71.88 (59.05-82.06) | |  |  | |  |
| **Newton**[24], 1999 | ICD-9 430, 431, 432.0-1, 432.9, 434, 436 | stroke first confirmed within 60 days | 91 (80-97) | 84 (79-87) | 45.22 (36-55) | 98 (96-99) | | |  | High |
|  |  | stroke first mentioned/confirmed within 60 days | 86 (74-92) | 85 (81-88) | 51.30 (42-61) | 97 (94-98) | | |  |  |
|  |  | stroke confirmed any time | 92 (82-97) | 85 (81-89) | 52.17 (43-62) | 98 (96-99) | | |  |  |
| **Palmieri**[25], 2007 | ICD-9 430-434, 436-438 | fatal cerebrovascular event (definite stroke, stroke associated with definite or possible MI, or unclassifiable), men |  |  | 74.47 (69.33-79.02) |  | | |  | Medium |
|  |  | fatal cerebrovascular event (definite stroke, stroke associated with definite or possible MI, or unclassifiable), women |  |  | 78.17 (72.14-83.22) |  | | |  |  |
|  |  | fatal cerebrovascular event (definite stroke, stroke associated with definite or possible MI, or unclassifiable), total |  |  | 75.99  (72.18-79.43) |  | | |  |  |
| **Piriyawat**[26], 2002 | ICD-9 430, 431, 432.9, 433, 434, 435, 436, 437.1, 437.6; primary or secondary position | cerebrovascular disease | 89.04 (86.36-91.26) |  | 72.76 (69.54-75.76) |  | | |  | High |
| **Ramalle-Gomara**[27], 2013 | ICD-9-CM 430-435, primary diagnosis | stroke | 90.1 (85.2-95.0) | 89.1 (85.0-93.3) | 84.9 (79.2-90.5) | 93.0 (89.5-96.5) | | |  | High |
|  | ICD-9-CM 430-435, primary or secondary diagnosis |  | 88.8 (83.5-94.2) | 79.4 (74.2-84.7) | 72.6 (65.9-79.3) | 92.1 (88.2-95.9) | | |  |  |
|  | ICD-9-CM 430-434, primary diagnosis | stroke | 86.2 (79.8-92.5) | 90.0 (86.2-93.8) | 80.6 (73.6-87.5) | 93.1 (89.8-96.4) | | |  |  |
|  | ICD-9-CM 430-434, primary or secondary diagnosis |  | 85.4 (79.0-91.9) | 79.6 (74.6-84.6) | 66.9 (59.4-74.3) | 91.9 (88.2-95.6) | | |  |  |
|  | ICD-9-CM 430, 431, 434, 436, primary diagnosis | stroke | 73.7 (66.3-81.0) | 97.6 (95.5-99.7) | 94.9 (90.5-99.3) | 85.8 (81.6-90.1) | | |  |  |
|  | ICD-9-CM 430, 431, 434, 436, primary or secondary diagnosis |  | 75.0 (67.8-82.2) | 94.3 (91.3-97.4) | 89.1 (83.3-94.9) | 86.0 (81.7-90.3) | | |  |  |
| **Reker**[28], 2001 | high specificity algorithm: ICD-9 431.x, 433.x1, 434.x1 as admission diagnosis only | new stroke | 53.76 (47.72-59.70) | 87.24 (83.44-90.30) | 75.00 (68.30-80.72) | 72.61 (68.30-76.54) | | |  | High |
|  | high-specificity algorithm: ICD-9 431.x, 433.x1, 434.xx in any diagnostic (admission or discharge or secondary) field |  | 59.14 (53.10-64.92) | 83.93 (79.83-87.35) | 72.37 (66.00-77.97) | 74.27 (69.88-78.22) | | |  |  |
|  | high-sensitivity algorithm: ICD-9 430.x, 431.x, 432.x, 434.xx, 436.x in any diagnostic (admission or discharge or secondary) field |  | 91.04 (86.90-94.00) | 39.80 (34.95-44.85) | 51.84 (41.31-56.33) | 86.19 (80.09-90.70) | | |  |  |
| **Roumie**[29], 2008 | ICD-9 430, 431, 433.x1, 434 (not 434.x0), 436 | new outpatient stroke (no inpatient diagnosis in prior year) as primary diagnosis |  |  | 96.55 (92.73-98.48) |  | | |  | High |
|  |  | new outpatient stroke (no inpatient diagnosis in prior year) as primary or secondary diagnosis |  |  | 88.74 (83.78-92.38) |  | | |  |  |
|  |  | true incident stroke (no inpatient diagnosis in prior year, no history of remote stroke) in primary position |  |  | 73.89 (67.19-79.67) |  | | |  |  |
|  |  | true incident stroke (no prior inpatient or outpatient diagnosis) in primary position |  |  | 80.12 (73.18-85.66) |  | | |  |  |
|  |  | true incident stroke (either primary position and no prior inpatient outpatient diagnosis, or secondary diagnosis or with prior outpatient stroke diagnosis) |  |  | 67.97 (61.47-73.85) |  | | |  |  |
| **Shahar**[30], 1995 | ICD-9 hospital discharge code in any position: 431, 432, 434, 436, 437 | overall (all years), against WHO stroke criteria for acute stroke |  |  | 63.00 (61-65) |  | | |  | High |
|  |  | overall (all years), against MSS stroke criteria for acute stroke |  |  | 42.10 (40-44) |  | | |  |  |
| **Stegmayr**[31], 1992 | ICD 430-434, 436 as hospital discharge diagnosis | acute stroke, nonfatal |  |  | 68.46 (67.16-69.73) |  | | |  | High |
|  | ICD 430-434, 436 as immediate or underlying COD | acute stroke, fatal |  |  | 90.32 (88.15-92.14) |  | | |  |  |
| **Szczesniewska**[32], 1990 | ICD-9 430-434, 436-438 as initial COD | stroke as definite or possible COD | 63.19 (57.67-68.39) |  | 96.71 (93.07-98.55) |  | | |  | Medium |
| **Thigpen**[33], 2015 | ICD-9 430-434, 436; all diagnostic positions | acute stroke (amongst all potential AF cases) |  |  | 94.15 (92.94-95.17) |  | | |  | Medium |
|  | ICD-9 430-434, 436; primary position only |  |  |  | 97.15 (96.08-97.94) |  | | |  |  |
|  | ICD-9 430-434, 436; all diagnostic positions | acute stroke (only amongst true-positive AF cases) |  |  | 82.2 (80.3-83.9) |  | | |  |  |
|  | ICD-9 430-434, 436; all diagnostic positions | acute stroke (excluding trauma-related ICH) |  |  | 72.8 (70.7-74.9) |  | | |  |  |
| **Tirschwell**[34], 2002 | ICD-9-CM 430, 431, 433.x1, 434 (excluding 434.x0), 436, up to nine discharge positions | stroke overall |  |  |  |  | | | 0.79 (0.68-0.90) | High |
|  | ICD-9-CM 430, 431, 433.x1, 434 (excluding 434.x0), 436, first two discharge positions |  |  |  |  |  | | | 0.77 (0.66-0.87) |  |
|  | ICD-9-CM 430, 431, 433.x1, 434 (excluding 434.x0), 436, primary discharge position only |  |  |  |  |  | | | 0.74 (0.64-0.84) |  |
| **Tu**[35], 2013 | ICD-9 362.3, 430, 431, 434.x, 436, 435.x or ICD-10 160.x, I61.x, I63.x (excluding 163.6), I64, H34.1, G45.x (excluding G45.4), H34.0 as inpatient hospitalization diagnosis |  | 36.9 (27.6-46.2) | 99.8 (99.7-99.9) | 80.9 (69.6-92.1) | 98.7 (98.4-99.0) | | |  | High |
|  | ICD-9 362.3, 430, 431, 434.x, 436, 435.x or ICD-10 160.x, I61.x, I63.x (excluding 163.6), I64, H34.1, G45.x (excluding G45.4), H34.0 as ER, or inpatient or day-case hospitalization diagnosis |  | 45.6 (36.0-55.3) | 99.7 (99.5-99.8) | 73.4 (62.6-84.3) | 98.9 (98.6-99.2) | | |  |  |
|  | ICD-9 362.3, 430, 431, 434.x, 436, 435.x or ICD-10 160.x, I61.x, I63.x (excluding 163.6), I64, H34.1, G45.x (excluding G45.4), H34.0 as inpatient hospitalization diagnosis or OHIP codes 432 435, or 436 as an outpatient diagnosis |  | 74.8 (66.4-83.1) | 97.6 (97.2-98.1) | 39.9 (33.0-46.8) | 99.5 (99.3-99.7) | | |  |  |
|  | ICD-9 362.3, 430, 431, 434.x, 436, 435.x or ICD-10 160.x, I61.x, I63.x (excluding 163.6), I64, H34.1, G45.x (excluding G45.4), H34.0 as ER, or inpatient or day-case hospitalization diagnosis; or OHIP codes 432 435, or 436 as an outpatient diagnosis |  | 75.7 (67.4-84.0) | 97.5 (97.1-98.0) | 39.2 (32.4-46.0) | 99.5 (99.3-99.7) | | |  |  |
|  | ≥ 1 ICD-9 362.3, 430, 431, 434.x, 436, 435.x or ICD-10 160.x, I61.x, I63.x (excluding 163.6), I64, H34.1, G45.x (excluding G45.4), H34.0 as inpatient hospitalization diagnosis or, ≥ 2 OHIP codes 432 435, or 436 as an outpatient diagnosis within one year |  | 60.2 (50.7-69.6) | 99.2 (99.0-99.5) | 62.0 (52.5-71.5) | 99.2 (98.9-99.4) | | |  |  |
|  | ≥ 1 ICD-9 362.3, 430, 431, 434.x, 436, 435.x or ICD-10 160.x, I61.x, I63.x (excluding 163.6), I64, H34.1, G45.x (excluding G45.4), H34.0 as ER, or inpatient or day-case hospitalization diagnosis or, ≥ 2 OHIP codes 432 435, or 436 as an outpatient diagnosis within one year |  | 62.1 (52.8-71.5) | 99.1 (98.8-99.4) | 59.3 (50.0-68.5) | 99.2 (99.0-99.5) | | |  |  |
|  | ≥ 1 ICD-9 362.3, 430, 431, 434.x, 436, 435.x or ICD-10 160.x, I61.x, I63.x (excluding 163.6), I64, H34.1, G45.x (excluding G45.4), H34.0 as inpatient hospitalization diagnosis or, ≥ 2 OHIP codes 432 435, or 436 as an outpatient diagnosis within two years |  | 61.2 (51.8-70.6) | 99.2 (99.0-99.5) | 61.8 (52.3-71.2) | 99.2 (98.9-99.4) | | |  |  |
|  | ≥ 1 ICD-9 362.3, 430, 431, 434.x, 436, 435.x or ICD-10 160.x, I61.x, I63.x (excluding 163.6), I64, H34.1, G45.x (excluding G45.4), H34.0 as inpatient hospitalization diagnosis or, ≥ 2 OHIP codes 432 435, or 436 as an outpatient diagnosis within three years |  | 61.2 (51.8-70.6) | 99.2 (99.0-99.5) | 61.8 (52.3-71.2) | 99.2 (98.9-99.4) | | |  |  |
|  | ≥ 1 ICD-9 362.3, 430, 431, 434.x, 436, 435.x or ICD-10 160.x, I61.x, I63.x (excluding 163.6), I64, H34.1, G45.x (excluding G45.4), H34.0 as inpatient hospitalization diagnosis or, ≥ 3 OHIP codes 432 435, or 436 as an outpatient diagnosis within one year |  | 49.5 (39.9-59.2) | 99.4 (99.2-99.7) | 65.4 (54.8-75.9) | 98.9 (98.7-99.2) | | |  |  |
|  | ≥ 1 ICD-9 362.3, 430, 431, 434.x, 436, 435.x or ICD-10 160.x, I61.x, I63.x (excluding 163.6), I64, H34.1, G45.x (excluding G45.4), H34.0 as inpatient hospitalization diagnosis or, ≥ 3 OHIP codes 432 435, or 436 as an outpatient diagnosis within two years |  | 50.5 (40.8-60.1) | 99.4 (99.2-99.7) | 65.8 (55.4-76.3) | 99.0 (98.7-99.2) | | |  |  |
|  | ≥ 1 ICD-9 362.3, 430, 431, 434.x, 436, 435.x or ICD-10 160.x, I61.x, I63.x (excluding 163.6), I64, H34.1, G45.x (excluding G45.4), H34.0 as inpatient hospitalization diagnosis or, ≥ 3 OHIP codes 432 435, or 436 as an outpatient diagnosis within three years |  | 50.5 (40.8-60.1) | 99.4 (99.2-99.6) | 65.0 (54.5-75.5) | 99.0 (98.7-99.2) | | |  |  |
| **Wildenschild**[36], 2013 | ICD-10 I61, I63, I64 | acute stroke | 55.32 (40.24-69.54) |  | 54.17 (39.31-68.36) |  | | |  | Medium |

95% CI=95% confidence interval; AF=atrial fibrillation; CM=Clinical Modification; COD=cause-of-death; ICD=International Classification of Diseases; ICH=intracerebral haemorrhage; MI=myocardial infarction; MSS=Minnesota Stroke Survey; NPV=negative predictive value; OHIP=Ontario Health Insurance Plan Physician Claims Database; PPV=positive predictive value; SAH=subarachnoid haemorrhage; TIA=transient ischaemic attack; WHO=World Health Organization

1. Aboa-Eboulé C, Mengue D, Benzenine E, Hommel M, Giroud M, Béjot Y, et al. How accurate is the reporting of stroke in hospital discharge data? A pilot validation study using a population-based stroke registry as control. J Neurol. 2013;260: 605–613. doi:10.1007/s00415-012-6686-0

2. Agrawal N, Johnston SC, Wu YW, Sidney S, Fullerton HJ. Imaging data reveal a higher pediatric stroke incidence than prior US estimates. Stroke J Cereb Circ. 2009;40: 3415–3421. doi:10.1161/STROKEAHA.109.564633

3. Appelros P, Terént A. Validation of the Swedish inpatient and cause-of-death registers in the context of stroke. Acta Neurol Scand. 2011;123: 289–293. doi:10.1111/j.1600-0404.2010.01402.x

4. Arnason T, Wells PS, van Walraven C, Forster AJ. Accuracy of coding for possible warfarin complications in hospital discharge abstracts. Thromb Res. 2006;118: 253–262. doi:10.1016/j.thromres.2005.06.015

5. Birman-Deych E, Waterman AD, Yan Y, Nilasena DS, Radford MJ, Gage BF. Accuracy of ICD-9-CM codes for identifying cardiovascular and stroke risk factors. Med Care. 2005;43: 480–485.

6. Broderick J, Brott T, Kothari R, Miller R, Khoury J, Pancioli A, et al. The Greater Cincinnati/Northern Kentucky Stroke Study: preliminary first-ever and total incidence rates of stroke among blacks. Stroke J Cereb Circ. 1998;29: 415–421.

7. Davenport RJ, Dennis MS, Warlow CP. The accuracy of Scottish Morbidity Record (SMR1) data for identifying hospitalised stroke patients. Health Bull (Edinb). 1996;54: 402–405.

8. Ellekjaer H, Holmen J, Krüger O, Terent A. Identification of incident stroke in Norway: hospital discharge data compared with a population-based stroke register. Stroke J Cereb Circ. 1999;30: 56–60.

9. Ghia D, Thomas PR, Cordato DJ, Worthington JM, Cappelen-Smith C, Griffith N, et al. Validation of emergency and final diagnosis coding in transient ischemic attack: South Western Sydney transient ischemic attack study. Neuroepidemiology. 2010;35: 53–58. doi:10.1159/000310338

10. Heckbert SR, Kooperberg C, Safford MM, Psaty BM, Hsia J, McTiernan A, et al. Comparison of self-report, hospital discharge codes, and adjudication of cardiovascular events in the Women’s Health Initiative. Am J Epidemiol. 2004;160: 1152–1158. doi:10.1093/aje/

11. Holick CN, Turnbull BR, Jones ME, Chaudhry S, Bangs ME, Seeger JD. Atomoxetine and cerebrovascular outcomes in adults. J Clin Psychopharmacol. 2009;29: 453–460. doi:10.1097/JCP.0b013e3181b2b828

12. Ives DG, Fitzpatrick AL, Bild DE, Psaty BM, Kuller LH, Crowley PM, et al. Surveillance and ascertainment of cardiovascular events. The Cardiovascular Health Study. Ann Epidemiol. 1995;5: 278–285.

13. Kokotailo RA, Hill MD. Coding of stroke and stroke risk factors using international classification of diseases, revisions 9 and 10. Stroke J Cereb Circ. 2005;36: 1776–1781. doi:10.1161/01.STR.0000174293.17959.a1

14. Köster M, Asplund K, Johansson Å, Stegmayr B. Refinement of Swedish administrative registers to monitor stroke events on the national level. Neuroepidemiology. 2013;40: 240–246. doi:10.1159/000345953

15. Kumamaru H, Judd SE, Curtis JR, Ramachandran R, Hardy NC, Rhodes JD, et al. Validity of claims-based stroke algorithms in contemporary Medicare data: reasons for geographic and racial differences in stroke (REGARDS) study linked with medicare claims. Circ Cardiovasc Qual Outcomes. 2014;7: 611–619. doi:10.1161/CIRCOUTCOMES.113.000743

16. Lakshminarayan K, Anderson DC, Jacobs DR Jr, Barber CA, Luepker RV. Stroke rates: 1980-2000: the Minnesota Stroke Survey. Am J Epidemiol. 2009;169: 1070–1078. doi:10.1093/aje/kwp029

17. Lakshminarayan K, Larson JC, Virnig B, Fuller C, Allen NB, Limacher M, et al. Comparison of Medicare claims versus physician adjudication for identifying stroke outcomes in the Women’s Health Initiative. Stroke J Cereb Circ. 2014;45: 815–821. doi:10.1161/STROKEAHA.113.003408

18. Leibson CL, Naessens JM, Brown RD, Whisnant JP. Accuracy of hospital discharge abstracts for identifying stroke. Stroke J Cereb Circ. 1994;25: 2348–2355.

19. Lentine KL, Schnitzler MA, Abbott KC, Bramesfeld K, Buchanan PM, Brennan DC. Sensitivity of billing claims for cardiovascular disease events among kidney transplant recipients. Clin J Am Soc Nephrol CJASN. 2009;4: 1213–1221. doi:10.2215/CJN.00670109

20. Leone MA, Capponi A, Varrasi C, Tarletti R, Monaco F. Accuracy of the ICD-9 codes for identifying TIA and stroke in an Italian automated database. Neurol Sci Off J Ital Neurol Soc Ital Soc Clin Neurophysiol. 2004;25: 281–288. doi:10.1007/s10072-004-0355-8

21. Leppälä JM, Virtamo J, Heinonen OP. Validation of stroke diagnosis in the National Hospital Discharge Register and the Register of Causes of Death in Finland. Eur J Epidemiol. 1999;15: 155–160.

22. Lindblad U, Råstam L, Ranstam J, Peterson M. Validity of register data on acute myocardial infarction and acute stroke: the Skaraborg Hypertension Project. Scand J Soc Med. 1993;21: 3–9.

23. Mayo N, Danys I, Carlton J, Scott S. Accuracy of hospital discharge coding for stroke. Can J Cardiol. 1993;9: 121D.

24. Newton KM, Wagner EH, Ramsey SD, McCulloch D, Evans R, Sandhu N, et al. The use of automated data to identify complications and comorbidities of diabetes: a validation study. J Clin Epidemiol. 1999;52: 199–207.

25. Palmieri L, Barchielli A, Cesana G, de Campora E, Goldoni CA, Spolaore P, et al. The Italian register of cardiovascular diseases: attack rates and case fatality for cerebrovascular events. Cerebrovasc Dis Basel Switz. 2007;24: 530–539. doi:10.1159/000110423

26. Piriyawat P, Smajsová M, Smith MA, Pallegar S, Wabil A Al-, Garcia NM, et al. Comparison of active and passive surveillance for cerebrovascular disease: The Brain Attack Surveillance in Corpus Christi (BASIC) Project. Am J Epidemiol. 2002;156: 1062–1069.

27. Ramalle-Gomara E, Ruiz E, Serrano M, Bartulos M, Gonzalez M-A, Matute B. Validity of Discharge Diagnoses in the Surveillance of Stroke. Neuroepidemiology. 2013;41: 185–188. doi:10.1159/000354626

28. Reker DM, Hamilton BB, Duncan PW, Yeh SC, Rosen A. Stroke: who’s counting what? J Rehabil Res Dev. 2001;38: 281–289.

29. Roumie CL, Mitchel E, Gideon PS, Varas-Lorenzo C, Castellsague J, Griffin MR. Validation of ICD-9 codes with a high positive predictive value for incident strokes resulting in hospitalization using Medicaid health data. Pharmacoepidemiol Drug Saf. 2008;17: 20–26. doi:10.1002/pds.1518

30. Shahar E, McGovern PG, Sprafka JM, Pankow JS, Doliszny KM, Luepker RV, et al. Improved survival of stroke patients during the 1980s. The Minnesota Stroke Survey. Stroke J Cereb Circ. 1995;26: 1–6.

31. Stegmayr B, Asplund K. Measuring stroke in the population: quality of routine statistics in comparison with a population-based stroke registry. Neuroepidemiology. 1992;11: 204–213.

32. Szczesniewska D, Kurjata P, Broda G, Polakowska M, Kupsc W. Comparison of official mortality statistics with data obtained from myocardial infarction and stroke registers. Rev Dépidémiologie Santé Publique. 1990;38: 435–439.

33. Thigpen JL, Dillon C, Forster KB, Henault L, Quinn EK, Tripodis Y, et al. Validity of international classification of disease codes to identify ischemic stroke and intracranial hemorrhage among individuals with associated diagnosis of atrial fibrillation. Circ Cardiovasc Qual Outcomes. 2015;8: 8–14. doi:10.1161/CIRCOUTCOMES.113.000371

34. Tirschwell DL, Longstreth WT Jr. Validating administrative data in stroke research. Stroke J Cereb Circ. 2002;33: 2465–2470.

35. Tu K, Wang M, Young J, Green D, Ivers NM, Butt D, et al. Validity of administrative data for identifying patients who have had a stroke or transient ischemic attack using EMRALD as a reference standard. Can J Cardiol. 2013;29: 1388–1394. doi:10.1016/j.cjca.2013.07.676

36. Wildenschild C, Mehnert, Frank, W. Thomsen R, Iversen H, Vestergaard K, Ingeman, Annette A, et al. Registration of acute stroke: validity in the Danish Stroke Registry and the Danish National Registry of Patients. Clin Epidemiol. 2013; 27. doi:10.2147/CLEP.S50449
