# Supplementary material for: Validity of Diagnostic Codes for Acute Stroke in Administrative Databases: A Systematic Review
Source: PLoS One. 2015 Aug 20;10(8):e0135834. doi: 10.1371/journal.pone.0135834 (PMC4546158; doi:10.1371/journal.pone.0135834)
Supplement: S4 Table — (DOCX) [file pone.0135834.s010.docx]

**S4 Table. Results of Studies Validating Diagnoses of Fatal Stroke in Administrative Data.**

| First Author, Year | Diagnostic Codes | Parameter | Sensitivity (95% CI) | Specificity (95% CI) | PPV (95% CI) | NPV (95% CI) | Quality |
| --- | --- | --- | --- | --- | --- | --- | --- |
| Appelros[1], 2011 | ICD-10 I61, I63, I64 | stroke as main or contributing COD |  |  | 79.59 (70.00-86.68) |  | High |
| Brown[2], 2006 | ICD-10 I60-I69 as underlying COD on death certificate | acute stroke: neurologist 1 | 66.67 (51.97-78.85) |  | 45.95 (34.44-57.87) |  | High |
|  |  | acute stroke: neurologist 2 | 62.71 (49.11-74.66) |  | 50.00 (38.25-61.75) |  |  |
| de Faire[3], 1976 | ICD-1965 430-438 | cerebrovascular disease as COD on death certificate | 96.00 (89.49-98.71) | 99.72 (99.10-99.93) | 96.97 (90.76-99.21) | 99.62 (98.96-99.88) | High |
| Iso[4], 1990 | ICD-8 or ICD-9 430-438 | stroke as underlying COD | 65.93 (60.39-71.08) |  | 97.66 (94.33-99.14) |  | Medium |
|  | ICD-8 431 or ICD-9 431-432 |  |  |  | 98.82 (92.71-99.94) |  |  |
|  | ICD-8 432-434, ICD-9 433-434 |  |  |  | 100 (92.26-100) |  |  |
|  | ICD-8 431 or ICD-9 431-432 | intracranial haemorrhage as COD | 71.08 (59.94-80.25) |  | 69.41 (58.34-78.70) |  |  |
|  | ICD-8 432-434, ICD-9 433-434 | non-haemorrhagic/occlusive stroke as COD | 43.24 (31.94-55.25) |  | 91.47 (84.91-95.45) |  |  |
| Ives[5], 1995 | ICD-9-CM 430-438 | stroke as COD | 42.11 (20.25-66.50) |  | 61.54 (31.58-86.14) |  | High |
| Koster[6], 2013 | ICD-10 I61, I63, I64 as COD | definite or possible stroke, incident or recurrent |  |  | 25.81 (20.86-31.43) |  | High |
|  |  | definite or possible stroke, incident |  |  | 39.22 (29.85-49.41) |  |  |
|  |  | definite or possible stroke or unclassifiable, incident or recurrent |  |  | 62.00 (56.00-67.67) |  |  |
|  |  | definite or possible stroke or unclassifiable, incident |  |  | 87.25 (78.84-92.77) |  |  |
| Leibson[7], 1999 | ICD-9 430-438.9, first diagnosis | in-hospital stroke death | 55 (43-66) |  |  |  | High |
| Leppala[8], 1999 |  | COD on death certificate (all deaths) |  |  |  |  | High |
|  | ICD-8 or ICD-9 430 | SAH |  |  | 95.2 (83-99) |  |  |
|  | ICD-8 431.00,431.08,431.09,431.90,431.98,431.99 or ICD-9 431 | ICH |  |  | 91 (80-97) |  |  |
|  | ICD-8 432,433,434 or ICD-9 433,434 (excluding ICD-9 4330X, 4331X,4339X,4349X) | ischaemic stroke |  |  | 92.4 (94-97) |  |  |
|  | ICD-8 or ICD-9 430; ICD-8 431.00,431.08,431.09,431.90,431.98,431.99 or ICD-9 431; ICD-8 432,433,434 or ICD-9 433,434 (excluding ICD-9 4330X, 4331X,4339X,4349X) | any stroke, confirmed |  |  | 77 (70-83) |  |  |
|  |  | any stroke, confirmed or probable |  |  | 97 (93-99) |  |  |
|  |  | COD on death certificate (with a match in hospital database) |  |  |  |  |  |
|  | ICD-8 or ICD-9 430 | SAH |  |  | 100 |  |  |
|  | ICD-8 431.00,431.08,431.09,431.90,431.98,431.99 or ICD-9 431 | ICH |  |  | 100 |  |  |
|  | ICD-8 432,433,434 or ICD-9 433,434 (excluding ICD-9 4330X, 4331X,4339X,4349X) | ischaemic stroke |  |  | 89.5 |  |  |
|  | ICD-8 or ICD-9 430; ICD-8 431.00,431.08,431.09,431.90,431.98,431.99 or ICD-9 431; ICD-8 432,433,434 or ICD-9 433,434 (excluding ICD-9 4330X, 4331X,4339X,4349X) | any stroke, confirmed |  |  | 97.5 |  |  |
| Lindblad[9], 1993 | ICD 430-438 | acute stroke as COD on death certificate |  |  | 92 (78-98) |  | High |
| Palmieri[10], 2007 |  | definite stroke, definite stroke associated with MI, or unclassifiable as COD on death certificate |  |  |  |  | Medium |
|  | ICD-9 430-434, 436-438 |  |  |  | 75.99 (72.18-79.43) |  |  |
|  | ICD-9 430 |  |  |  | 90.00 (75.40-96.75) |  |  |
|  | ICD-9 431 |  |  |  | 80.25 (72.99-86.00) |  |  |
|  | ICD-9 434 |  |  |  | 85.33 (74.85-92.10) |  |  |
|  | ICD-9 436 |  |  |  | 72.82 (65.91-78.81) |  |  |
|  | ICD-9 433 or 434 |  |  |  | 86.25 (76.31-92.61) |  |  |
|  | ICD-9 434 or 436 |  |  |  | 76.30  (70.68-81.15) |  |  |
|  | ICD-9 433, 434, or 436 |  |  |  | 76.73 (71.19-81.50) |  |  |
|  | ICD-9 430, 431, or 434 |  |  |  | 83.09 (77.98-87.24) |  |  |
|  | ICD-9 430, 431, 434, or 436 |  |  |  | 78.80 (74.75-82.36) |  |  |
| Phillips[11], 1993 | ICD-9 430-438 | acute stroke as COD on death certificate | 82.35 (55.80-95.33) |  |  |  | High |
| Rampitage[12], 2013 | ICD-10 I60-69 | cerebrovascular disease as underlying COD | 31.5 (21.8-39.9) |  | 60.7 (42.1-77.0) |  | High |
| Rao[13], 2007 | ICD-10 I60-I69 | cerebrovascular disease as underlying COD | 81.6 (78.0-84.8) |  | 88.4 (85.2-91.1) |  | High |
|  | ICD-10 I60 | SAH as underlying COD | 60 |  |  |  |  |
|  | ICD-10 I61 | ICH as underlying COD | 69 |  |  |  |  |
|  | ICD-10 I63 | cerebral infarction as underlying COD | 66 |  |  |  |  |
| Reggio[14], 1995 | ICD-9 430-438 | stroke as initial COD |  |  | 54.31 (44.81-63.59) |  | Medium |
|  |  | stroke as initial or intermediate COD |  |  | 52.43 (44.98-59.81) |  |  |
|  |  | stroke as initial, intermediate, or terminal COD | 60.00 (38.67-78.87) |  | 52.33 (45.04-59.55) |  |  |
| Stegmayr[15], 1992 | ICD 430-434, 436 | acute stroke as immediate or underlying COD |  |  | 90.32 (88.15-92.14) |  | High |
| Szczesniewska[16], 1990 | ICD-9 430-434, 436-438 as initial COD | stroke as definite or possible COD | 63.19 (57.67-68.39) |  | 96.71 (93.07-98.55) |  | Medium |
| Tolonen[17], 2007 | ICD-9 430-434, 436-438 or ICD-10 I60-I69 | any stroke as COD on death certificate | 86 (84-88) |  | 92 (90-94) |  | High |
|  | ICD-9 430 or ICD-10 I60 | SAH | 95 (91-99) |  | 97 (94-100) |  |  |
|  | ICD-9 431 or ICD-10 I61 | ICH | 95 (92-98) |  | 92 (88-96) |  |  |
|  | ICD-9 433, 434, 436, or ICD-10 I63,I64 | ischaemic stroke | 79 (75-83) |  | 83 (80-86) |  |  |
|  | ICD-9 433, 434 or ICD-10 I63 | ischaemic stroke | 76 (72-80) |  | 77 (73-81) |  |  |

95% CI=95% confidence interval; CM=Clinical Modification; COD=cause-of-death; ICD=International Classification of Diseases; ICH=intracerebral haemorrhage; MI=myocardial infarction; NPV=negative predictive value; PPV=positive predictive value; SAH=subarachnoid haemorrhage

1. Appelros P, Terént A. Validation of the Swedish inpatient and cause-of-death registers in the context of stroke. Acta Neurol Scand. 2011;123: 289–293. doi:10.1111/j.1600-0404.2010.01402.x

2. Brown DL, Senani F Al-, Lisabeth LD, Farnie MA, Colletti LA, Langa KM, et al. Defining cause of death in stroke patients: The Brain Attack Surveillance in Corpus Christi Project. Am J Epidemiol. 2007;165: 591–596. doi:10.1093/aje/kwk042

3. de Faire U, Friberg L, Lorich U, Lundman T. A validation of cause-of-death certification in 1,156 deaths. Acta Med Scand. 1976;200: 223–228.

4. Iso H, Jacobs DR Jr, Goldman L. Accuracy of death certificate diagnosis of intracranial hemorrhage and nonhemorrhagic stroke. The Minnesota Heart Survey. Am J Epidemiol. 1990;132: 993–998.

5. Ives DG, Fitzpatrick AL, Bild DE, Psaty BM, Kuller LH, Crowley PM, et al. Surveillance and ascertainment of cardiovascular events. The Cardiovascular Health Study. Ann Epidemiol. 1995;5: 278–285.

6. Köster M, Asplund K, Johansson Å, Stegmayr B. Refinement of Swedish administrative registers to monitor stroke events on the national level. Neuroepidemiology. 2013;40: 240–246. doi:10.1159/000345953

7. Leibson CL, Naessens JM, Brown RD, Whisnant JP. Accuracy of hospital discharge abstracts for identifying stroke. Stroke J Cereb Circ. 1994;25: 2348–2355.

8. Leppälä JM, Virtamo J, Heinonen OP. Validation of stroke diagnosis in the National Hospital Discharge Register and the Register of Causes of Death in Finland. Eur J Epidemiol. 1999;15: 155–160.

9. Lindblad U, Råstam L, Ranstam J, Peterson M. Validity of register data on acute myocardial infarction and acute stroke: the Skaraborg Hypertension Project. Scand J Soc Med. 1993;21: 3–9.

10. Palmieri L, Barchielli A, Cesana G, de Campora E, Goldoni CA, Spolaore P, et al. The Italian register of cardiovascular diseases: attack rates and case fatality for cerebrovascular events. Cerebrovasc Dis Basel Switz. 2007;24: 530–539. doi:10.1159/000110423

11. Phillips S, Cameron K, Chung C. Stroke surveillance revisited. Can J Cardiol. 1993;9: 124D.

12. Rampatige R, Gamage S, Peiris S, Lopez AD. Assessing the reliability of causes of death reported by the Vital Registration System in Sri Lanka: medical records review in Colombo. HIM J. 2013;42: 20–28.

13. Rao C, Yang G, Hu J, Ma J, Xia W, Lopez AD. Validation of cause-of-death statistics in urban China. Int J Epidemiol. 2007;36: 642–651. doi:10.1093/ije/dym003

14. Reggio A, Failla G, Patti F. Reliability of death certificates in the study of stroke mortality. A retrospective study in a Sicilian municipality. Ital J Neurol Sci. 1995;16: 567–570.

15. Stegmayr B, Asplund K. Measuring stroke in the population: quality of routine statistics in comparison with a population-based stroke registry. Neuroepidemiology. 1992;11: 204–213.

16. Szczesniewska D, Kurjata P, Broda G, Polakowska M, Kupsc W. Comparison of official mortality statistics with data obtained from myocardial infarction and stroke registers. Rev Dépidémiologie Santé Publique. 1990;38: 435–439.

17. Tolonen H, Salomaa V, Torppa J, Sivenius J, Immonen-Räihä P, Lehtonen A, et al. The validation of the Finnish Hospital Discharge Register and Causes of Death Register data on stroke diagnoses. Eur J Cardiovasc Prev Rehabil Off J Eur Soc Cardiol Work Groups Epidemiol Prev Card Rehabil Exerc Physiol. 2007;14: 380–385. doi:10.1097/01.hjr.0000239466.26132.f2
